# Synthesis and biological evaluation of substituted acetamide derivatives as potential butyrylcholinestrase inhibitors

**DOI:** 10.1038/s41598-023-31849-5

**Published:** 2023-03-25

**Authors:** Dehong Yu, Can Yang, Yi Liu, Tao Lu, Lizi Li, Gang Chen, Zerong Liu, Yanfang Li

**Affiliations:** 1 School of Chemical Engineering, Sichuan University, Chengdu, 610065 China; 2 Central Nervous System Drug Key Laboratory of Sichuan Province, Luzhou, 646106 China; 3 Sichuan Credit Pharmaceutical CO., Ltd., Luzhou, 646106 China

**Keywords:** Drug discovery, Medicinal chemistry, Structure-based drug design

## Abstract

Alzheimer’s disease (AD) is the most common type of age-related dementia. Inhibition of butyrylcholinesterase (BChE) emerge as an effective therapeutic target for AD. A series of new substituted acetamide derivatives were designed, synthesized and evaluated for their ability to inhibit BChE. The bioassay results revealed that several compounds displayed attractive inhibition against BChE). Among them, compound **8c** exhibited the highest BChE inhibition with IC_50_ values of 3.94 μM. Lineweaver Burk plot indicated that **8c** acted as a mixed-type BChE inhibitor. In addition, docking studies confirmed the results obtained through in vitro experiments, and showed that **8c** bound to the catalytic anionic site (CAS) and peripheral anionic site (PAS) of BChE active site. Meanwhile, its ADME parameters were approximated using in silico method. Molecular dynamics simulation studies on the complex of **8c**-BChE were performed, RMSD, RMSF, Rg, SASA, and the number of hydrogen bonds were calculated as well. These results implied that **8c** could serve as appropriate lead molecule for the development of BChE inhibitor.

## Introduction

As a progressive neurodegenerative disease characterized by cognitive loss, more than 47 million persons suffer Alzheimer’s disease (AD) worldwide. Accordingly, it is also the most common form of dementia, accounting for 50 ~ 60% of the total patients of dementia in individuals over 65 years old^1^. Unfortunately, the etiology of AD is not fully understood now, researchers know only that the decrease of acetylcholine (ACh) in cerebral cortex and hippocampus is the most common neurotransmitter deficiency in AD^2^. In addition, several factor, such as tau protein aggregation oxidative stress, and metal ion disorder are considered to related to this disease in the pathophysiology of AD^3,4^.

Currently, the classic "cholinergic hypothesis" has been widely accepted by researchers^5,6^. In which, acetylcholinesterase (AChE) and butyrylcholinesterase (BChE), as two important enzymes in the nervous system, can hydrolyze ACh and thus play a key role by regulating the level of ACh in the brain. Noticeably, their kinetics and selectivity for substrate ACh are different^7,8^. In healthy brain and early stage of AD, AChE and BChE exist in a ratio of 4:1, thus, AChE is selectively responsible for hydrolysis of the neurotransmitter ACh, while BChE seems to only play a supporting role^9^. However, In the process of AD, AChE activity in AD brain gradually decreased by up to 45%, while BChE activity in cortex and hippocampus enhanced as much as twice^9^. Owing to its broader substrate specificity than AChE, BChE becomes the main ACh degrading enzyme in advanced AD. Additionally, selective BChE inhibition can avoid the common side effects (classical cholinergic toxicity) of AChE inhibitors^10^. No physiological defects in BChE knockout mice could be observed^11^. Similarly, BuChE silent people can also live healthily to an old age^12^. In this regard, selective inhibition against BChE represents an effective strategy for AD treatment, especially in late AD. Therefore, in recent years, the focus of researcher switched from AChE to BChE.

Human BChE (hBChE), consists of 574 amino acids, has three disulfide bridges and a number of glycosylation sites. Its hydrophobic active site is composed of 55 residues that form a 20 Å deep gorge^13^. Near the entrance to the active site, residues Phe329, Asp70, and Tyr332 direct cationic substrates deeper into the active site. While the catalytic triad of Ser198, His438, and Glu325 is located at the bottom of the gorge^14^. Owing to the substitutions of six amino acids, the active site of hBChE is about 200 Å larger than that of human AChE (hAChE). In particular, two aromatic residues Phe295 and Phe297 in acyl-binding site of hAChE are replaced by Leu286 and Val288 in hBChE^8,15^. Thus, the substrate specificity of BChE increase due to the accommodation of larger side chains into its acyl-binding pocket, which provides the possibility of designing of selective inhibitors against BChE^16^.

In our previous study, a new BChE inhibitor **970180** with good activity was found using the strategy of virtual screening from commercial database Enamine. As a mixed-type BChE inhibitor, **970180** acted and displayed good ADME properties, low toxicity, and attractive neuroprotective activity^17^. Since this molecule is chemically unrelated to other potent BChE inhibitors reported previously. So, in current study, we aimed to design and synthesize a new series of selective BChE inhibitors based on the skeleton of 970180. Furthermore, preliminary kinetic and modeling studies were also preformed to investigate the inhibition mechanism of derivatives.

## Results and discussion

### Design

**Figure 1:**
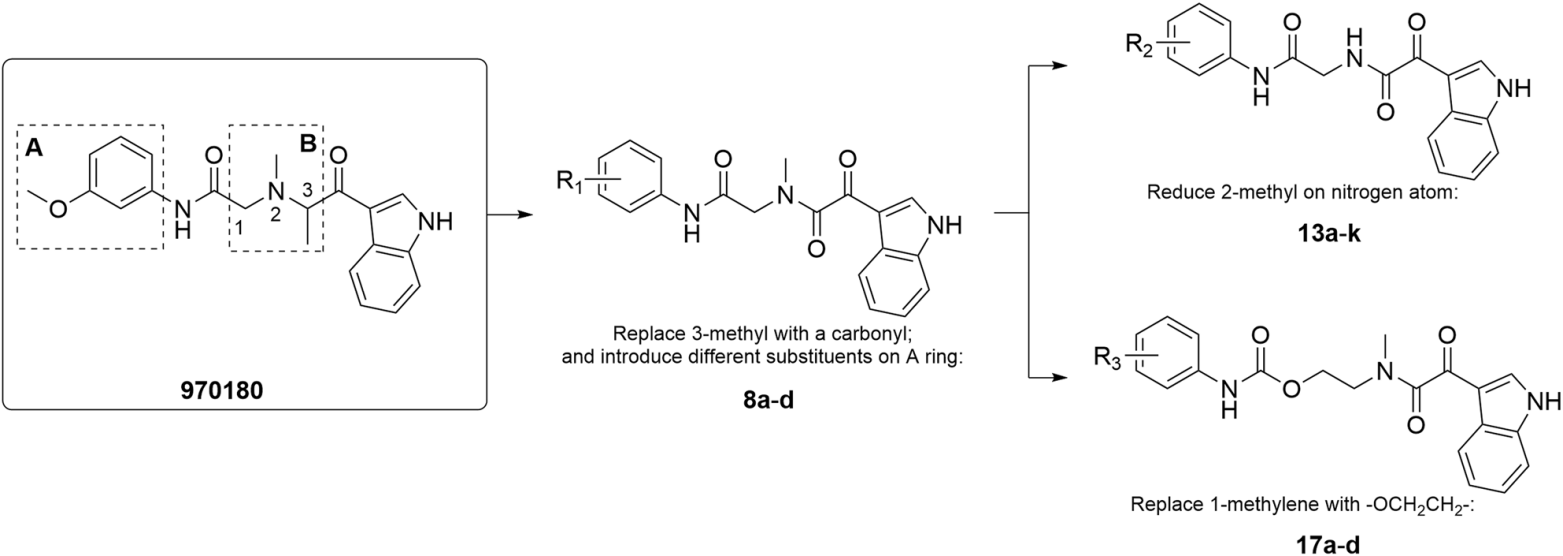
Design strategy carried out starting from **970180** molecule.

The specific modification strategy was shown in Fig. 1, which was performed on the basis of **970180** molecule, with fragment A being a phenyl ring bearing a *meta-*methoxy and fragment B being a nitrogen-containing linker group. According to the binding mode of **970180** with BChE^17^, its indole moiety extended in the choline binding pocket of BChE and exhibited two significant interactions, one was the π–π stacking interaction between the benzene ring with key amino acid Trp82, another was the hydrogen bond between nitrogen atom and His 438. Meanwhile, the phenyl ring in fragment A presented same π–π stacking interaction with Trp231 of acyl binding pocket, besides, the oxygen in fragment B formed another hydrogen bond with Phe329. Chierrito et al. reported the synthesis of a series of indolylpiperidines hybrids, these hybrid molecules can occupy the catalytic region of BChE in a folded conformation and therefore showed very potent and selective hBChE inhibition, low cellular toxicity, and in vivo target engagement. In which, the aromatic ring of indole moiety involves the aromatic interaction with Trp82 of the BuChE^18^. Further revealed by Meden et al., oxidation to oxindole or reduction to indoline resulted in diminished BChE inhibition, and the same applied to N-methyl substitution and introduction of a polar 5-hydroxy group on the indole^19^. Taking these important interactions and previous findings into consideration, the indole moiety of **970180** keep unchanged. For fragment B, we attempted to introduce different substituents at positions 1, 2, and 3 to investigate their inhibitory potency against BChE. Firstly, the methyl group at position 3 was replaced with carbonyl group, then, three small substituents, such as methyl, methoxy, and fluorine at different position on phenyl ring, were introduced into fragment A. Subsequently, in order to explore the effects of 2-methyl on BChE inhibition capacity, the methyl group on the nitrogen atom was reduced and provide **13a**–**k**. In addition, in order to investigate the influence of carbon chain elongation on the inhibition potency, carbon atom at position 1 was replaced with an oxygen atom to form an ester structure and thereby joined two carbon atoms.

### Chemistry

**Scheme 1:**
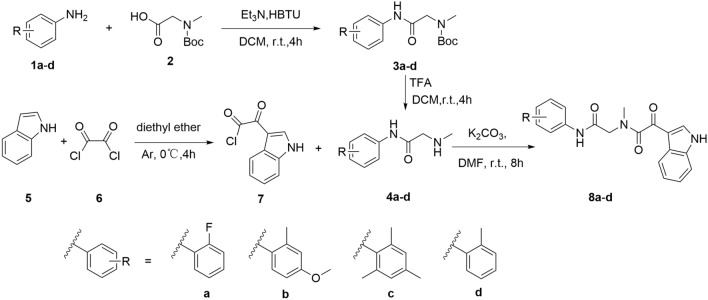
Synthesis of compounds **8a–d**.

**Scheme 2:**
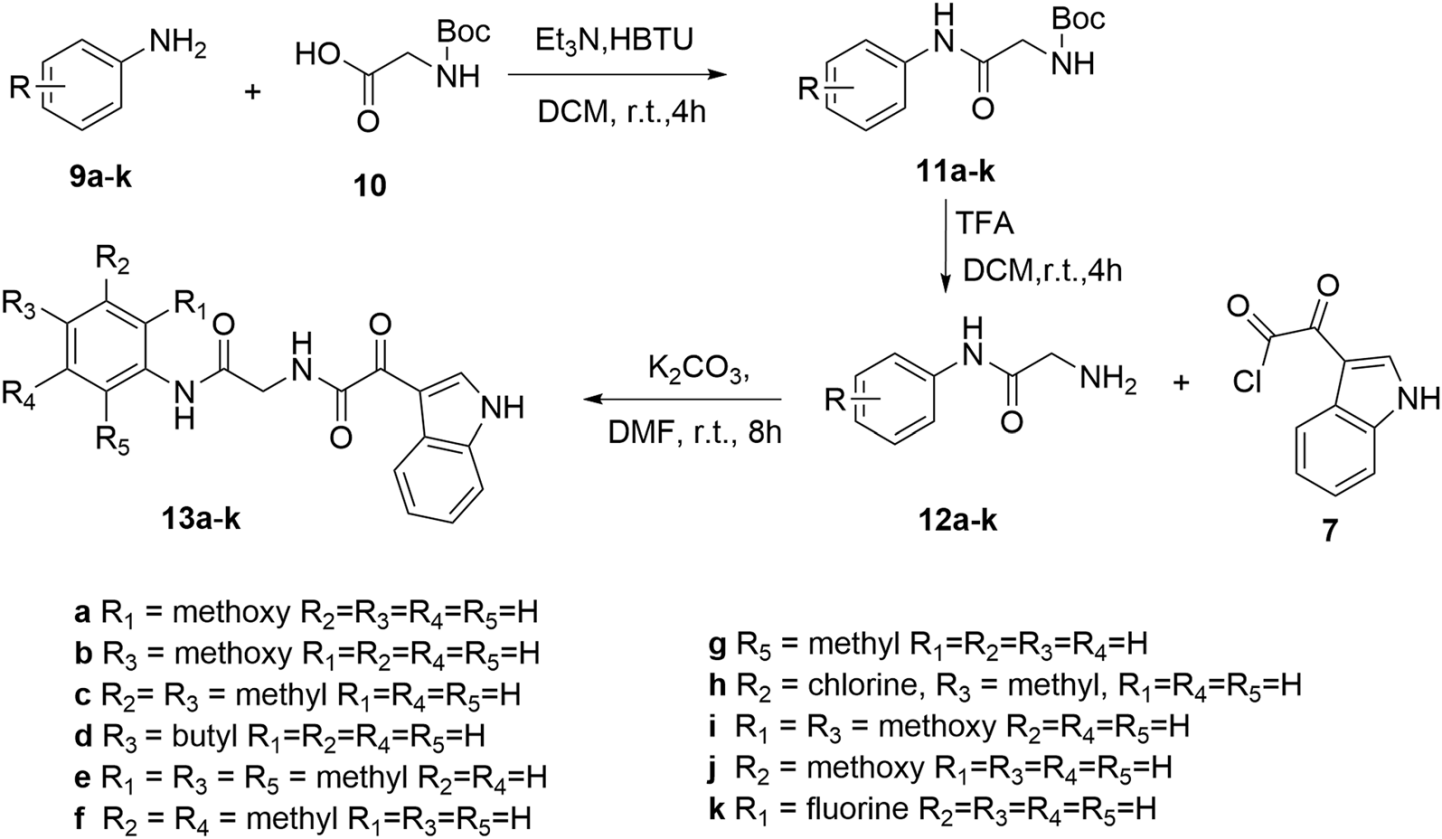
Synthesis of compounds **13a–k**.

**Scheme 3:**
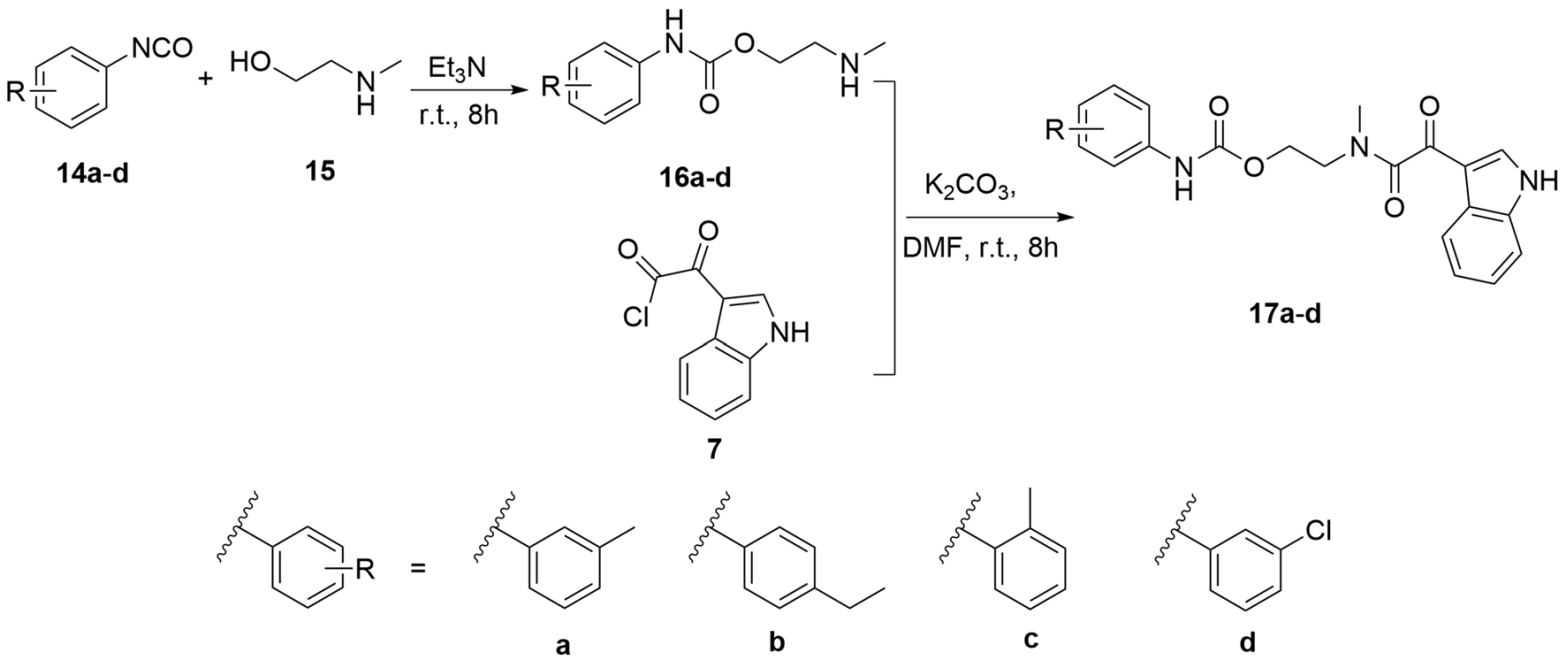
Synthesis of compounds **17a–d**.

Derivatives **8a–d** were obtained according to Scheme 1. The synthetic route started with different substituted anilines **1a–d**, which reacted with Boc-protected sarcosine **2** through condensation reaction to obtain intermediates **3a–d**. Subsequently, intermediates **4a–d** were obtained after Boc deprotection, which was treated with indole-3-glyoxylyl chloride **7** obtaining from the reaction of indole **5** and oxalyl chloride **6** smoothly yielded targets **8a–d**. Meanwhile, the synthetic route of **13a–k** was outlined in Scheme 2, which was similar to that of **8a–d**. Later, as shown in Scheme 3, the synthetic route of **17a–d** was initiated from isocyanates **14a–d** with different substituents, which reacted with N-Methyl-2-hydroxy-ethylamine **15** to afford **16a–d**. Final products **17a–d** were obtained with **7** and **16** as reactants in the catalytic effect of potassium carbonate at 25 °C.

### Evaluation of BChE inhibition and structure–activity relationship (SAR) analysis

All synthesized molecules were evaluated for inhibitory properties against BChE in vitro. Tacrine and galantamine were taken as controls. As shown in Table 1, two of derivatives (**8c**, and **8d**) displayed BChE inhibition below 50 μM and presented moderate activity with IC_50_ values ranging from 3.94 ± 0.15 to 19.60 ± 0.21 μM. The most potent molecule was **8c** (IC_50_ = 3.94 ± 0.16 μM).

**Table.1:** The chemical structure of the synthesized compounds and their IC_50_ values against BChE.


Compds	R_1_/R_3_	BChE: IC_50_ (μM) ± SEM	Compds	R_2_	BChE: IC_50_ (μM) ± SEM
**8a**		> 50	**13a**		> 50
**8b**		> 50	**13b**		> 50
**8c**		3.94 ± 0.15	**13c**		> 50
**8d**		19.60 ± 0.21	**13d**		> 50
**17a**		> 50	**13e**		> 50
**17b**		> 50	**13f**		> 50
**17c**		> 50	**13g**		> 50
**17d**		> 50	**13h**		> 50
			**13i**		> 50
			**13j**		> 50
			**13k**		> 50
**Galantamine**	/	44.1 ± 0.91	**Tacrine**	/	0.14 ± 0.01

The starting compound **970180** exhibited excellent in vitro inhibitory potency with an IC_50_ value of 4.24 ± 0.16 μM. Firstly, the methyl group at position 3 was replaced with a carbonyl group and introduced simultaneously three small substituents (methyl, methoxy, and fluorine) at different positions on ring A to provide derivatives **8a–d**. Among these series, two compounds displayed attractive BChE inhibition (**8c–d**, IC_50_ = 3.94, and 19.60 μM, respectively), the most potent compound **8c** exhibited little increase of inhibitory potency than **970180**. Introducing methyoxy group on *para* position resulted in the decline of activity (**8b** vs. **8d**, IC_50_ > 50 μM vs. IC_50_ = 19.60 μM). Noticeably, further increase the number of methyl group on ring A from one to three to provide **8c**, which exhibited the most potency (**8c** vs. **8d**, IC_50_ = 3.94 μM vs. IC_50_ = 19.60 μM). The above results were possible to confirm that increasing the number of methyl group was beneficial for the improvement of inhibitory activity.

In addition, in order to investigate the contribution of 2-methyl on the nitrogen atom, **13a–k** were synthesized and showed poor activity toward BChE (**13a–k**, IC_50_ > 50 μM), which revealed that the 2-methyl on the nitrogen atom played a prominent role for the maintenance of inhibition. Therefore, the 2-methyl on nitrogen atom were reserved, instead, the 1-methylene was replaced with an oxygen atom and the carbon chain length of two carbon atoms was extended. Unfortunately, no obvious improvement in the potency was observed in this series, this fact pointed out the importance of keeping substituents at positions 1 and 2 unchanged.

Liu et al. designed, synthesized, and evaluated a series of carbamate derivatives of N-salicyloyl tryptamine as multifunctional therapeutic agents for the treatment of Alzheimer's disease (AD). One of the compounds was effective for improving learning and memory behavior, blood–brain barrier permeation, pharmacokinetics, ChE inhibition, and anti-neuroinflammation^20^. In a similar study conducted by Lu et al. Structured-based molecular modification guided the synthesis of an aromatic tertiary amine derivatives. Two optimal compounds presented selective BChE inhibitory (hBChE < 20 nM, eeAChE > 10 μM), good BBB permeation, primary cell safety, and restorative effect on cognitive impairment in vivo test^21^.

### Kinetic study

**Figure 2:**
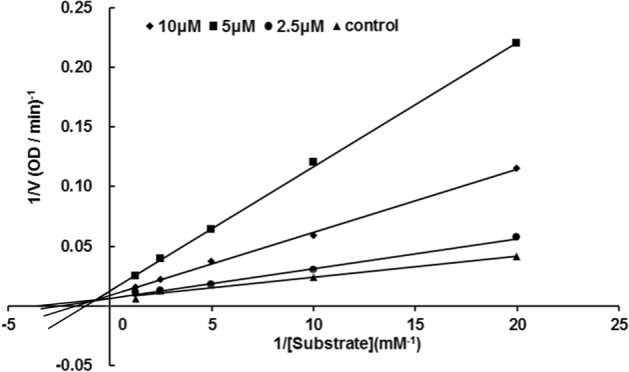
Lineweaver–Burk plots of **8c** on BChE.

Toward a better understanding of the inhibition mechanism, the kinetics of **8c** as the most potent BChE inhibitor was performed. The inhibition type was determined by analyzing the Lineweaver–Burk plots^22^. As shown in Fig. 2, the curves of **8c** in the Lineweaver–Burk plots inhibiting BChE intersected in the second quadrant, which indicated that both *V*_*max*_ and *K*_*m*_ changed with the increase of inhibitory concentration after the adding of inhibitor, and this pattern suggested that **8c** acts as a mixed BChE inhibitor. (*V*_*max*_ = 105.26, *K*_*m*_ = 0.24, *K*_*i*_ = 3.27 µM).

### Binding mode analysis

As far as we know, the catalytic active site (CAS) of BChE was comprised of a choline binding pocket (Trp82), a peripheral anion site (Asp70), a catalytic residue (Ser198, His438, and Glu325), and an acyl-binding pocket (Val288, Leu286, and Trp231)^23^. Among them, Ser198 and Trp82 are considered as key residues to maintain the enzyme activity^24^. In the recent decade, molecular docking has emerged as a common tool for investigating molecular recognition between ligand and protein based on the principle of “lock” and “key”^25^.

**Figure 3:**
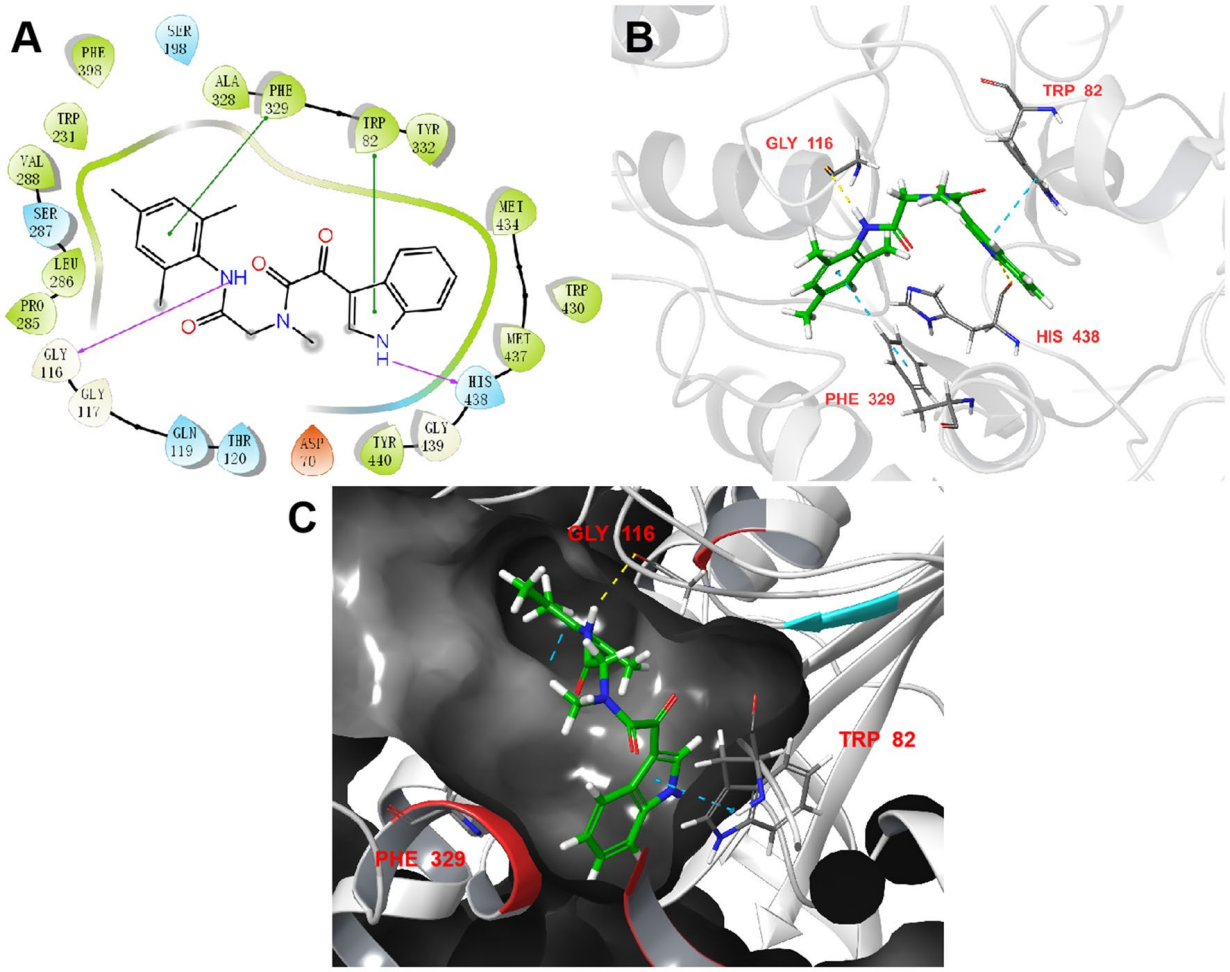
Docking pose of **8c** into active site of BChE (PDB ID 4TPK). (**A**) 2D binding mode of **8c** with BChE. Hydrogen bonds were shown as purple lines, π–π stacking interactions were in green lines. (**B**) interacting residues of BChE were shown in sticks colored by atom type, the carbon in green, hydrogen in white, nitrogen in blue and oxygen in red. Interactions included non-covalent bonds and π interaction, the hydrogen bonds in yellow, π–π stacking in blue. (**C**) 3D complex structure of molecule **8c** with BChE.

In this regards, molecular docking was employed to explore possible ligand-BChE interactions. The most potent compound **8c** were docked into the active site of hBChE (PDB entry 4TPK) using the Glide module of Schrödinger, several significant interactions can be observed from Fig. 3. The indole moiety of **8c** interacted with Trp82 via π–π stacking, which was similar with the results of Chierrito et al.^18^. The *m*-trimethylbenzene of **8c** contributed another π–π stacking with Phe329. Meanwhile, a H-bond interaction forms between an amide nitrogen with Gly116. In addition, the indole nitrogen atom H-bonds to His438. Taken together, these observations were in good agreement with that of kinetic study, rationalize the high affinity of **8c** for hBChE and explain its similar potency with **970180**. It can be derived from this result that the aromatic ring was essential for the inhibitory activity of **8c** against BChE since both H-bond and π–π stacking interactions were significant. Combined with the above-mentioned docking results and related literature reports, the H-bond interaction with His438 was regarded as crucial for the binding ability of BChE.

### Molecular dynamics simulation

Generally, the binding of ligand to protein is a dynamic phenomenon, which occurs in less than one nanosecond. Therefore, Molecular dynamics (MD) simulations over a period of 50 ns were carried out for **8c** with targeted enzyme (BChE), in order to analyze the stability, flexibility, and dynamic changes of **8c**-BChE complex. In the simulation process, the dynamic behavior and motion of **8c**-BChE complex were monitored by root mean squared deviation (RMSD), root mean squared fluctuation (RMSF), radius of gyration (Rg), solvent accessible surface area (SASA), and number of hydrogen bonds.

**Figure 4:**
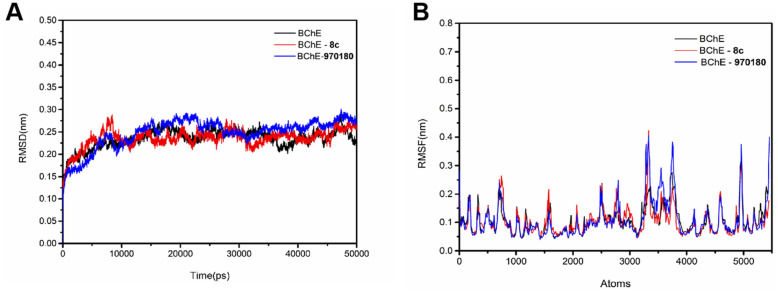
The RMSD (**A**) and RMSF (**B**) of **8c** with BChE.

The RMSD of the main chain atoms in **8c**-BChE complex was used to quantify the conformational change of protein during the simulation. Due to the influence of inhibitor, the BChE skeleton fluctuated greatly, the RMSD values (Fig. 4A) was within 0.20 ~ 0.30 nm and reached the maximum value of 0.3 nm at 8 ns, then fell back to about 0.25 nm at about 10 ns, and remained stable within the following 20 ns, with the oscillation amplitude not exceeding 0.1 nm. This indicated that the composite structure of **8c** and protein was relatively stable, and the **8c**-BChE complex reaches dynamic equilibrium^26^. Interestingly, both complexes (**8c** and **970180** with BChE, respectively) achieved balance almost at the same time (8 ns). On the other hand, the fluctuation ranges of the RMSD curve for **970180**-BChE complex (Fig. 4a) was slightly larger than **8c**-BuChE (Fig. 4b) at the same time, which goes in line with little improvements in BChE inhibition activity of **8c** compared with the starting point **970180**.

Additionally, the RMSF diagram showed the stability of key residues in the active cavity of BChE. The higher the fluctuating residues in the sequence, the more unstable the protein was^27^. The RMSF values of four key binding residues (Gly116, Trp82, Phe329 and His438) were further determined, as these residues were experimentally identified as stability indicators of BChE protein^28^. As shown in Fig. 4B, the highest RMSF value of **8c**-BChE complex was 0.2101, while the lowest RMSF value was 0.0783 in atoms 617 ~ 628 (Trp82), 895 ~ 896 (Gly116), 2497 ~ 2505 (Phe329), and 3412 ~ 3418 (His438), respectively. The maximum fluctuation was 0.1318 nm. The results suggested that the key amino acid residues remained stable and the conformation did not change significant after inhibitor **8c** bound with BChE.

**Figure 5:**
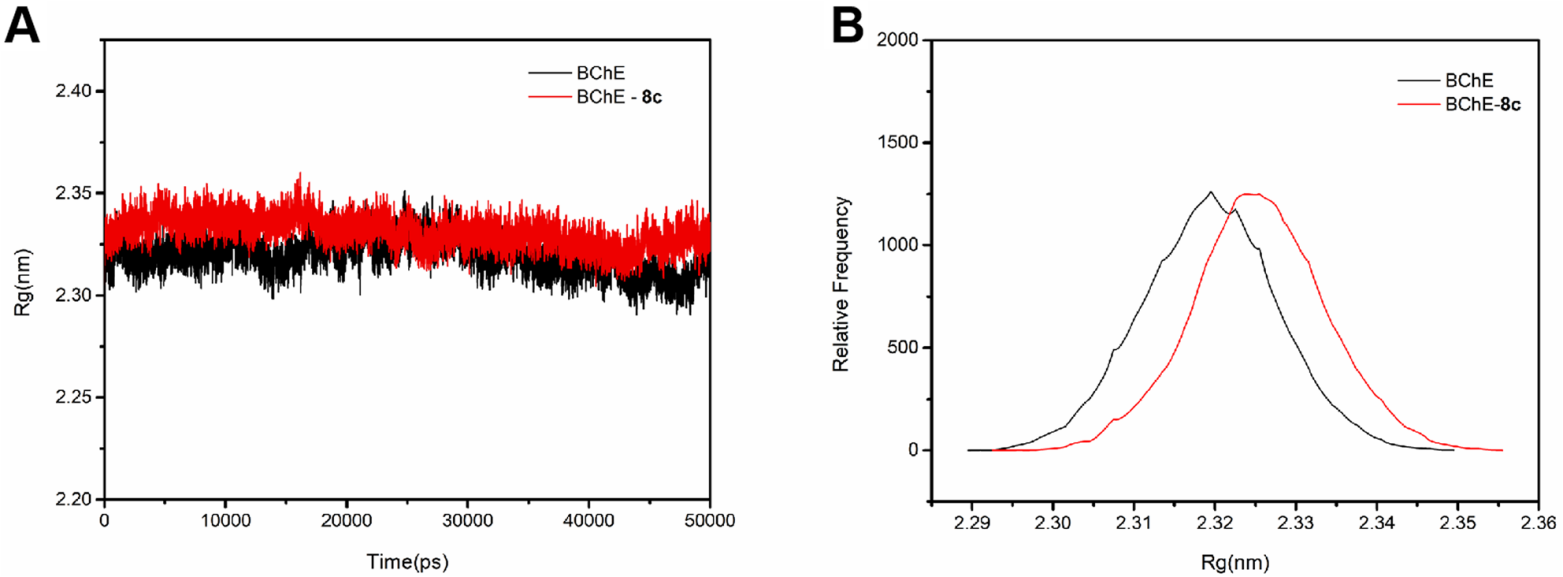
Changes in Rg values (**A**) and relative distribution frequency (**B**) of **8c**-BChE complex.

In addition, Rg is an important parameter for quantitative evaluation of protein structure compactness. A high Rg value represented that the protein conformation is more inclined to open the binding pocket to facilitate its interaction with the ligand^29^. Analyzing the variation plots of Rg vs simulation time (Fig. 5A), Rg value of **8c** in the complex was higher than that of the individual protein and remained in the narrow range of 2.30 ~ 2.35 nm during the simulation time. From the relative frequency analysis (Fig. 5B), compared with the free protein, the Rg value of the complex shifted 0.005 to the right. These facts meant that the protein conformation of **8c**-BChE complex tends even more to the open state in the docked pose.

**Figure 6:**
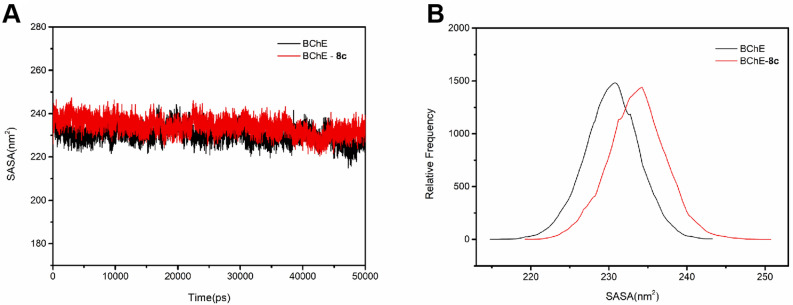
Changes in SASA values (**A**) and relative distribution frequency (**B**) of **8c**-BChE complex.

Considering the importance of SASA in indicating the hydrophobicity of protein, analysis on its fluctuation and relative frequency were performed. During the whole simulation process, the SASA value keep relatively stable in the range from 230 to 245 nm Fig. 6A. Noticeably, SASA value of **8c**-BChE complex increased obviously compared with that of individual BChE (Fig. 6B), this implied that the binding of **8c** with BChE led to the opening of the hydrophobic cavity of BChE protein.

**Figure 7:**
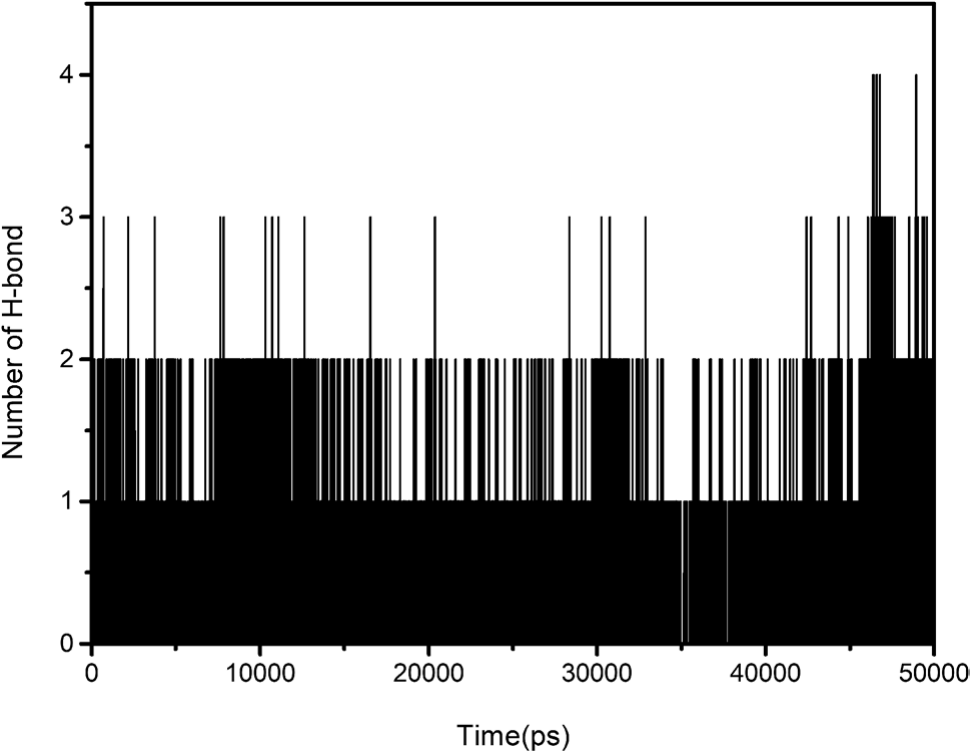
Number of hydrogen bonds between **8c** and BChE.

Besides, hydrogen bond analysis plays a crucial role in predicting the stability of protein–ligand complex. In general, the high number of intermolecular hydrogen bonds means the high stability of protein–ligand complex. The number of hydrogen bonds (Fig. 7) between the active site of BChE and inhibitor **8c** during MD simulation was calculated by analyzing MD trajectories. The maximum number of hydrogen bond reached four and maintained one to three hydrogen bonds throughout the entire simulation trajectory, which suggested that **8c** has a good combination with BChE protein via stable H-bond interaction.

### In silico ADME prediction

As mentioned by Ferreira et al.^30^, a good drug candidate molecule should also possess proper ADME properties, including absorption, distribution, metabolism and excretion. Therefore, in the process of hit identification and optimization, estimating ADME properties of hit compound is of great importance. Multiple ADME properties of **8c** were predicted in silico using the QikProp v. 5.5 (Schrödinger) so as to evaluate its drug-like properties. Noticeably, most of central nervous system (CNS) drugs possess low molecular weights (MWs) in the range of 400 ~ 600 Da or lower than 400 Da. Meanwhile, marketed CNS-acting drugs usually have a mean MW of 310 Da^31^. Besides, as an important parameter, QPlogBB value predicts the brain/blood partition coefficient and is applied to select drugs that can cross the blood–brain barrier. As presented in Table 2, the QPlogBB value of **8c** was within the ideal range (− 3.0 ~ 1.2), indicating that it may be able to enter the CNS by oral administration. In addition, the human oral absorption (HOA) value of **8c** was higher than 50, revealing better gastrointestinal absorption. Compared with the characteristics of galantamine and tacrine, the data indicated that **8c** also had a high binding capacity to human serum protein and adequate clog P, low levels of primary metabolites, and a good Caco-2 permeability. In short, all the above results implied that **8c** can be administered orally as CNS-active compound.

**Table 2:** ADME prediction results of inhibitor **8c**, **Galantamine**, **and Tacrine.**

NO.	ADME Prediction	8c	Galantamine	Tacrine	Recommended values
1	CNS	− 1	1	1	− 2 (inactive) − 2 (active)
2	MW	377.44	287.4	198.3	130–725
3	SASA	652.50	509.3	419.9	300.0–1000.0
4	Volume	1196.974	900	689	500.0–2000.0
5	Donor HB	1	1	1.5	0.0–6.0
6	Accpt HB	6.5	5.2	2	2.0–20.0
7	QPlogPo/w	3.59	2.01	2.50	− 2.0–6.5
8	QPlogS	− 4.96	− 2.2	− 2.9	− 6.5–0.5
9	QPPCaco	834.79	758	3374	< 25 poor, > 500 great
10	QPlogBB	− 0.82	0.38	0.10	− 3.0–1.2
11	metab	4	4	3	1–8
12	QPlogKhsa	0.39	0.01	0.02	− 1.5–1.5
13	HOA%	100.00	90	100	< 25% poor, > 80% high
14	PSA	94.16	43.30	31.57	7.0–200.0
15	Rule of five	0	0	0	Maximum is 4
16	Rule of three	0	0	0	Maximum is 3

*CNS* Predicted central nervous system activity, − 2 (inactive) − 2 (active), *MW* Molecular weight of the molecule, *SASA* Total solvent-accessible surface area, in square angstroms, using a probe with a 1.4 Å radius, *Volume* Total solvent-accessible volume, in cubic angstroms, using a probe with a 1.4 Å radius, *Donor HB* Estimated number of hydrogen bonds that would be donated by the solute, *Accpt HB* Estimated number of hydrogen bonds that would be accepted by the solute, *QPlogPo/w* Predicted octanol/water partition coefficient, *QPlogS* Predicted aqueous solubility. *S* in mol/dm^3^, is the concentration of the solute’s saturated solution that is in equilibrium with crystalline solid, *QPPCaco* Predicted apparent Caco-2 cell permeability in nm/s (a model for the gut–blood barrier), *QPlogBB* Predicted brain/blood partition coefficient, *Metab* Number of primary metabolites, *QPlogKhsa* Prediction of binding to human serum albumin, *%HOA* Predicted qualitative human oral absorption, *PSA* Van der Waal surface area of polar nitrogen and oxygen atoms, *ROF* Number of violations of Lipinski’s rule of five, molecular weight < 500, *QPlogPo/w < 5* number of hydrogen bond donors ≤ 5, number of hydrogen bond acceptors HB ≤ 10, *ROT* Number of violations of Jorgensen’s rule of three (QPlogS > − 5.7, QPCaco > 22 nm/s, number of primary metabolites < 7).

## Conclusion

In summary, 21 new substituted acetamide derivatives were designed and synthesized to discover selective BChE inhibitors for the treatment of AD, and their inhibitory potency against BChE were evaluated. Two of them showed attractive BChE inhibition (IC_50_ < 50 μM). Among them, **8c** presented the most potent inhibition against BChE with IC_50_ value of 3.947 ± 0.15 μM. Structure–activity relationship analysis revealed that the design strategy of replacing 3-methyl with a carbonyl group was feasible, but reducing 2-methyl on the nitrogen atom and introducing ester group at position 3 had a negative influence on the inhibitory activity. And increasing the methyl group on the A ring from one to three significantly improved the inhibition capacity. Docking study further predicted the binding mode of **8c**-BChE complex and provided a reasonable explanation for its high activity. Moreover, the results of enzyme kinetics assay suggested that **8c** acted as a mixed-type inhibitor. The MD simulation results confirmed the structure of **8c**-BChE complex was relatively stable, the RMSD value did not exceed 0.35 nm during the simulation process.

## Methods and materials

### General apparatus and chemicals

All common chemicals were purchased from commercial sources and used without further purification. The water used was distilled water. Reaction progress was monitored using analytical thin layer chromatography (TLC, detected by UV light 254 nm) on precoated silica gel HSGF_254_ plates (Qingdao Haiyang Chemical Plant, China). Column chromatography was performed on silica gel (200 ~ 300 mesh; Qingdao Dingkang Chemical Inc.). Horse serum butyrylcholinesterase (BChE, EC 3.1.1.8, from equine serum), 5, 5-dithiobis (2-nitrobenzoic) acid (DTNB), butyrylthiocholine iodide, galantamine and tacrine were purchased from Sigma Aldrich (St. Louis, MO, USA).^1^H NMR and ^13^C NMR spectra were measured using tetramethylsilane (TMS, δ 0 ppm) as the internal standard in dimethyl sulfoxide (DMSO‑*d*_6_) solutions on a JNM-ECZ400S/L1 (JEOL Ltd., Japan), Chemical shifts were reported in parts per million (ppm, δ) downfield from TMS. Proton coupling patterns are described as singlet (s), doublet (d), double doublet (dd), triplet (t), and multiplet (m). ESI–MS date were recorded on a Waters Quattro Premier XE triquadrupole mass spectrometer (Waters Corporation, USA), while ESI-HRMS date were recorded on a Q-TOF Priemier mass spectrometer (Micromass, Manchester, UK). The enzyme kinetic assay was performed on an EnSpire Enzyme Marker (Thermos Fisher, USA). Purity for final compounds (higher than 95%) were determined by HPLC‑ELSD [Alltech Lab Alliance Modells 201 system instrument, equipped with Alltech ELSD 6000 detector and Cromasil C_18_ column (250 mm × 4.6 mm, 5 μm), mobile phase: methanol/0.1% formic acid (40/70 ~ 100/0, in 45 min), 1 mL/min].

### Chemistry

#### General procedure for the synthesis of **3**

Boc-sarcosine **2** (1.75 g, 10 mmol) was dissolved in dry CH_2_Cl_2_ (30 mL) and cooled to 0 °C, NEt_3_ (1.53 mL, 11 mmol) was added, followed the addition of HBTU (4.17 g, 11 mmol) slowly, the mixture was keep stirring for 1 h and the corresponding aniline compound **1a–d** (10 mmol) was added, then, the reaction was allowed to rise to room temperature (r. t.) and stirred vigorously for further 4 h. The crude product was added with ethyl acetate, the resulting organic layer was washed 3 times with saturated NaHCO_3_ solution and saturated NaCl solution. The organic layer was collected and dried over anhydrous Na_2_SO_4_. After removing Na_2_SO_4_ by filtration, the solvent was removed *in vacuum* to give the products **3a–d** (yield, 81 ~ 90%).

#### General procedure for the synthesis of **4a–d**

The intermediate **3a–d** (10 mmol) was dissolved in a mixture of TFA: DCM (10 mL, 1:1), then stirred for 4 h at r.t. After the reaction, the crude product was treated according to the procedure described in 4.2.1 to afford **4a–d** (yield 85 ~ 91%).

#### General procedure for the synthesis of **7**

Indole** 5** (6 g, 50 mmol) was dissolved in a 50 mL three-neck round bottom flask with 60 mL anhydrous ether under argon. After dissolving completely in the bottle, oxalyl chloride **6** (6 mL, 60 mmol) of was slowly added dropwise and the mixture was stirred for 1 h at r.t. Then The reaction system was washed three times with anhydrous ether and filtered, and the obtained solid was product **7** (yellow solid, yield 81%). ^1^H NMR (400 MHz, acetone- *d*_6_) δ 8.75 (d, *J* = 12.2 Hz, 1H), 8.31 (s, 1H), 7.58 (s, 1H), 7.30 (s, 2H).

#### General procedure for the synthesis of **11a–k**

Boc-sarcosine compound **10** (1.88 g, 10 mmol) was dissolved in dry CH_2_Cl_2_ (30 mL) and cooled to 0 °C, NEt_3_ (1.53 mL, 11 mmol) was added, followed the addition of HBTU (4.17 g, 11 mmol) slowly, keep stirring for 1 h and was then added to the corresponding aniline compound **9a–k** (10 mmol), the mixture was allowed to warm up to r.t. and stirred vigorously for further 4 h. The crude product was treated according to the procedure described in 4.2.1 to afford **11a–k** (yield 82 ~ 93%).

#### General procedure for the synthesis of **12a–k**

The intermediate product **11a–k** (10 mmol) was dissolved in a mixture of TFA: DCM (10 mL, 1:1) and allowed to stir for 4 h at r.t. After the reaction, the reaction solution was washed 3 times with saturated NaHCO_3_ solution and NaCl solution, extracted with ethyl acetate. The resulting organic fractions were combined, washed with plenty of distilled water and dried over Na_2_SO_4_. After removing Na_2_SO_4_ by filtration, the organic phase was concentrated under reduced pressure to afford the product **12a–k** (yield, 89 ~ 95%).

#### General procedure for the synthesis of **16a–d**

The isocyanate compound **14a–d** (1.17 g, 10 mmol) was dissolved in 30 mL of CH_2_Cl_2,_ followed by addition of NEt_3_ (4.17 mL, 30 mmol) and N-methyl-2-hydroxy-ethylamine **5** (0.81 mL, 10 mmol), keep stirring for 8 h at r.t. Then, the reaction was quenched by dilute HCl (v/v, HCl: H_2_O = 1:1.1) and extracted with ethyl acetate. The organic layer was washed with plenty of distilled water and dried over Na_2_SO_4_. After removing Na_2_SO_4_ by filtration, organic layer was concentrated *in vacuum* and purified by silica gel CC using petroleum ether/ethyl acetate (v/v, 1/1 ~ 1/2) as the elution to afford **16a–d** (yield, 25 ~ 51%).

#### General procedure for the synthesis of **8a–d**

Amide **4a–d** (0.3 mmol), indole-3-glyoxylyl chloride **7** (54.07 mg), and K_2_CO_3_ (53.90 mg, as catalysis) were mixed and reacted at 25 °C for 8 h. The resulting products **8a–d** were separated by silica gel CC using petroleum ether/EtOAc (v/v, 2/1 ~ 1/1) as eluents.

##### N-(2-((2-fluorophenyl)amino)-2-oxoethyl)-2-(1H-indol-3-yl)-N-methyl-2-oxoacetamide (**8a**)

white solid, yield, 80%.^1^H NMR (400 MHz, DMSO-*d*_6_) δ = 12.32 (s, 1H), 10.11 (s, 1H), 8.31 (d, *J* = 3.2 Hz, 1H), 8.20 ~ 8.10 (m, 2H), 7.57 ~ 7.53 (m, 1H), 7.32 ~ 7.22 (m, 5H), 4.40 (d, *J* = 3.2 Hz, 2H), 3.01 (s, 3H). ^13^C NMR (100 MHz, DMSO-*d*_6_) δ 186.3, 168.2, 166.9, 162.3, 137.7, 137.5, 136.9, 124.8, 124.4, 124.1, 123.6, 123.5, 122.6, 120.9, 115.7, 115.5, 113.1, 112.7, 49.3, 35.7, 30.7. ESI–MS: *m/z* calcd. C_19_H_16_FN_3_O_3_ [M–H]^+^ 352.3, found 352.2. HPLC purity: 96.5%.

##### 2-(1H-indol-3-yl)-N-(2-((4-methoxy-2-methylphenyl)amino)-2-oxoethyl)-N-methyl-2-oxoacetamide (**8b**)

white solid, yield, 82%. ^1^H NMR (400 MHz, DMSO-*d*_6_) δ 12.27 (s, 1H), 9.53 (s, 1H), 8.34 (t, *J* = 3.4 Hz, 1H), 8.19–8.15 (m, 1H), 7.96 (s, 1H), 7.55 (ddd, *J* = 7.6, 3.1, 1.5 Hz, 1H), 7.30 ~ 7.27 (m, 2H), 6.85 ~ 6.59 (m, 2H), 4.27 (d, *J* = 4.9 Hz, 2H), 3.75 (s, 3H),3.01 (s, 3H), 2.22 (s, 3H). ^13^C NMR (100 MHz, DMSO-*d*_6_) δ186.1, 168.2, 166.6, 162.3, 156.9, 137.7, 136.7, 133.9, 128.8, 126.7, 124.8, 123.6, 122.6, 120.9, 115.4, 113.2, 112.6, 111.3, 55.1, 35.7, 30.7, 18.1. ESI–MS: *m/z* calcd. C_21_H_21_N_3_O_4_ [M–H]^+^ 378.4, found 378.2. HPLC purity:95.8%.

##### 2-(1H-indol-3-yl)-N-(2-(mesitylamino)-2-oxoethyl)-N-methyl-2-oxoacetamide (**8c**)

white solid, yield, 83%. ^1^H NMR (400 MHz, DMSO-*d*_6_) δ 12.25 (s, 1H), 9.49 (s, 1H), 8.30 (t, *J* = 3.2 Hz, 1H), 8.16 ~ 8.14 (m, 1H), 7.53 ~ 7.50 (m, 1H), 7.28 ~ 7.24 (m, 2H), 6.90 (d, *J* = 2.8 Hz, 2H), 4.35 (d, *J* = 4.8 Hz, 2H), 2.24 (s, 3H), 2.16 (s, 3H), 2.15 (s, 3H), 1.81 (s, 3H). ^13^C NMR (100 MHz, DMSO-*d*_6_): δ 186.9, 168.8, 167.0, 166.7, 138.4, 138.2, 137.4, 136.1, 135.4, 135.3, 132.7, 132.3, 128.8, 128.7, 125.3, 124.2, 123.1, 121.5, 113.7, 113.2, 113.2, 37.1, 34.1, 21.0, 18.6, 18.0. ESI–MS: *m/z* calcd. C_22_H_23_N_3_O_3_ [M–H]^+^ 376.4, found 376.2. HPLC purity: 97.0%.

##### 2-(1H-indol-3-yl)-N-methyl-2-oxo-N-(2-oxo-2-(o-tolylamino)ethyl)acetamide (**8d**)

white solid, yield, 82%. ^1^H NMR (400 MHz, DMSO-*d*_6_) δ 12.27 (s, 1H), 10.16 (s, 1H), 8.99 ~ 8.75 (m, 2H), 8.30 ~ 8.23 (m, 1H), 7.63 (dt, *J* = 7.1, 2.5 Hz, 2H), 7.56 (ddd, *J* = 4.6, 3.5, 2.2 Hz, 1H), 7.28 (dq, *J* = 6.8, 3.0, 2.2 Hz, 2H), 7.19 ~ 7.14 (m, 2H), 4.06 (d, *J* = 6.1 Hz, 2H), 2.89 (s, 3H), 2.74 (s, 3H). ^13^C NMR (100 MHz, DMSO-*d*_6_) δ 182.1, 167.6, 164.4, 162.9 139.2, 136.8, 135.8, 126.7, 124.0, 123.2, 121.9, 121.6, 121.5, 116.0, 115.8, 113.1, 112.8, 43.0, 36.3, 31.3. ESI–MS: *m/z* calcd. C_20_H_19_N_3_O_3_ [M–H]^+^ 348.3, found 348.1. HPLC purity:97.1%.

#### General procedure for the synthesis of **13a–k**

K_2_CO_3_ (53.90 mg, 0.39 mmol) was added to a solution of amides **12a–k** (0.3 mmol) and indole-3-glyoxylic chloride **7** (54.07 mg, 0.3 mmol in 2 mL of N, N-dimethylformamide and stir for 1 h at r.t. The target products **13a–k** was purified by silica gel CC with petroleum ether/ethyl acetate (v/v, 1/1 ~ 1/2) as the elution.

##### 2-(1H-indol-3-yl)-N-(2-((2-methoxyphenyl)amino)-2-oxoethyl)-2-oxoacetamide (**13a**)

White solid, yield, 90%.^1^H NMR (400 MHz, DMSO-*d*_*6*_) δ 12.27 (s, 1H), 9.28 (d, *J* = 4.0 Hz, 1H), 9.03 (t, *J* = 5.6 Hz, 1H), 8.80 (d, *J* = 3.6 Hz, 1H), 8.26 (m, 1H), 8.02 (d,* J* = 6.0 Hz, 1H), 7.55 (d, *J* = 4.4 Hz, 1H), 7.28 (q, *J* = 4.6 Hz, 2H), 7.08 (m, 2H), 6.92 (d, *J* = 6.4 Hz, 1H), 4.05 (d, *J* = 4.0 Hz, 2H), 3.82 (s, 3H). ^13^C NMR (100 MHz, DMSO-*d*_*6*_) δ 181.6, 169.2, 164.0, 138.7, 127.0, 126.1, 124.3, 123.5, 122.6, 121.3, 120.3, 112.6, 111.2, 55.7, 42.8. ESI–MS: *m/z* calcd. C_17_H_19_N_3_O_4_ [M–H]^+^ 350.3, found 350.2. HPLC purity: 98.8%.

##### 2-(1H-indol-3-yl)-N-(2-((4-methoxyphenyl)amino)-2-oxoethyl)-2-oxoacetamide (**13b**)

White solid, yield, 87%.^1^H NMR (400 MHz, DMSO-*d*_6_) δ 12.24 (s, 1H), 9.93 (s, 1H), 8.99 ~ 8.78 (m, 2H), 8.32 ~ 8.19 (m, 1H), 7.52 (dtd, *J* = 13.8, 7.1, 2.9 Hz, 3H), 7.30 ~ 7.19 (m, 2H), 7.03 ~ 6.85 (m, 2H), 4.02 (d, *J* = 6.4 Hz, 2H), 3.72 (s, 3H). ^13^C NMR (100 MHz, DMSO-*d*_6_) δ 181.5, 166.5, 163.8, 155.3, 138.6, 136.3, 131.9, 126.2, 123.5, 122.6, 121.3, 120.8, 113.9, 112.6, 112.2, 55.1, 42.4. ESI–MS: *m/z* calcd. C_17_H_19_N_3_O_4_ [M–H]^+^ 350.3, found 350.2. HPLC purity: 98.9%.

##### N-(2-((3,4-dimethylphenyl)amino)-2-oxoethyl)-2-(1H-indol-3-yl)-2-oxoacetamide (**13c**)

White solid, yield, 88%. ^1^H NMR (400 MHz, DMSO-*d*_6_) δ 12.25 (s, 1H), 9.91 (s, 1H), 8.88(m,1H), 8.81 (m, 1H), 8.26 (d, *J* = 4.6 Hz, 1H), 7.55 (m, 1H), 7.37 ~ 7.24 (m, 4H), 7.07 (dd, *J* = 8.3, 4.6 Hz, 1H), 4.03 (t, *J* = 5.2 Hz, 2H), 2.51 (s, 3H), 2.18 (s, 3H). ^13^C NMR (100 MHz, DMSO-*d*_6_) δ 181.5, 166.7, 163.9, 138.6, 136.5, 136.3, 131.0, 129.6, 126.2, 123.5, 122.6, 121.3, 120.4, 116.7, 112.6, 42.5, 19.6, 18.7. ESI–MS: *m/z* calcd. C_20_H_19_N_3_O_3_ [M–H]^+^ 348.3, found 348.2. HPLC purity: 98.9%.

##### N-(2-((4-butylphenyl)amino)-2-oxoethyl)-2-(1H-indol-3-yl)-2-oxoacetamide (**13d**)

white solid, yield, 85%. ^1^H NMR (400 MHz, DMSO-*d*_6_) δ 12.25 (s, 1H), 10.00 (s, 1H), 8.95 ~ 8.82 (t, 2H), 8.26 (m, 1H), 7.56 ~ 7.53 (m, 1H), 7.52 ~ 7.47 (m, 2H), 7.28 (m, 2H), 7.12 (m, 2H), 4.04 (d, *J* = 5.6 Hz, 2H), 2.53 (brs, 2H), 1.53 (m, 2H), 1.29 (m, 2H), 0.89 (t, *J* = 7.3 Hz, 3H). ^13^C NMR (100 MHz, DMSO-*d*_6_) δ 181.5, 166. 8, 163.8, 138.6, 137.3, 136.5, 136.3, 128.5, 126.2, 123.5, 122.6, 121.3, 119.2, 112.6, 112.2, 42.5, 34.2, 33.2, 21.7, 13.8. ESI–MS: *m/z* calcd. C_22_H_23_N_3_O_3_ [M–H]^+^ 376.4, found 376.2. HPLC purity: 98.4%.

##### 2-(1H-indol-3-yl)-N-(2-(mesitylamino)-2-oxoethyl)-2-oxoacetamide (**13e**)

white solid, yield, 91%. ^1^H NMR (400 MHz, DMSO-*d*_6_) δ 12.22 (s, 1H), 9.28 (s, 1H), 8.92 (t, *J* = 8.0 Hz 1H), 8.82 (brs, 1H), 8.25 (m, 1H), 7.55 (m, 1H), 7.27(m, 2H), 6.86 (s, 2H), 4.05 (d, *J* = 6.1 Hz, 2H), 2.22 (s, 3H), 2.12 (s, 3H), 2.11 (s, 3H). ^13^C NMR (100 MHz, DMSO-*d*_6_) δ 181.5, 166.9, 163.8, 138.6, 136.2, 135.4, 134.9, 128.2, 126.2, 123.4, 122.5, 121.3, 112.6, 42.1, 20.5, 17.9. ESI–MS: *m/z* calcd. C_21_H_21_N_3_O_3_ [M–H]^+^ 362.4, found 362.2. HPLC purity: 98.1%.

##### N-(2-((3,5-dimethylphenyl)amino)-2-oxoethyl)-2-(1H-indol-3-yl)-2-oxoacetamide (**13f**)

white solid, yield, 87%. ^1^H NMR (400 MHz, DMSO-*d*_6_) δ 12.28 (s, 1H), 9.90 (s, 1H), 8.88(m, 1H), 8.81 (m, 1H), 8. 8.25 (m, 1H), 7.53(m, 1H), 7.29 ~ 7.15 (m, 4H), 6.70 (s, 1H), 4.02 (d, *J* = 6.0 Hz, 2H), 2.24 (s, 6H). ^13^C NMR (100 MHz, DMSO-*d*_6_) δ 181.5, 166.9, 163.8, 138.6, 137.7, 126.2, 124.9, 123.5, 122.6, 121.3, 116.9, 112. 6, 42.6, 21.1. ESI–MS: *m/z* calcd. C_20_H_19_N_3_O_3_ [M–H]^+^ 348.3, found 348.1. HPLC purity: 99.1%.

##### 2-(1H-indol-3-yl)-2-oxo-N-(2-oxo-2-(o-tolylamino)ethyl)acetamide (**13g**)

white solid, yield, 85%. ^1^H NMR (400 MHz, DMSO-*d*_6_) δ 12.24 (s, 1H), 9.42 (s, 1H), 8.93 (s, 1H), 8.82 (m, 1H), 8.25 (m, 1H), 7.54 (m, 1H), 7.42 (d, *J* = 8.0 Hz, 1H), 7.30 ~ 7.12 (m, 5H), 4.09 (d, *J* = 6.4 Hz, 2H), 2.23 (s, 3H). ^13^C NMR (100 MHz, DMSO-*d*_6_) δ181.5, 167.0, 164.3, 138.61, 136.2, 135.4, 134.9, 128.2, 126.2, 123.4, 123.4, 122.5, 121.3, 112.6, 42.1, 20.5, 18.0. ESI–MS: *m/z* calcd. C_19_H_17_N_3_O_3_ [M–H]^+^ 334.3, found 334.2. HPLC purity: 98.6%.

##### N-(2-((3-chloro-4-methylphenyl)amino)-2-oxoethyl)-2-(1H-indol-3-yl)-2-oxoacetamide (**13h**)

white solid, yield, 86%. ^1^H NMR (400 MHz, DMSO-*d*_6_) δ 12.25 (s, 1H), 10.17 (s, 1H), 8.94 (d, *J* = 3.2 Hz, 1H), 8.81 (d, *J* = 3.2 Hz, 1H), 8.26 (dd, *J* = 6.4, 2.8 Hz, 1H), 7.79 (d, *J* = 2.4 Hz, 1H), 7.55 (m, 1H), 7.38 ~ 7.22 (m, 4H), 4.04 (d, *J* = 6.2 Hz, 2H), 2.27 (s, 3H). ^13^C NMR (100 MHz, DMSO-*d*_6_) δ 181.5, 167.2, 163.7, 138.6, 137.9, 136.3, 131.2, 129.9, 123.5, 122.6, 121.3, 119.1, 117.8, 112.6, 112.2, 42.6, 18.9. ESI–MS: *m/z* calcd. C_19_H_16_N_3_O_3_ [M–H]^+^ 368.8, found 368.1. HPLC purity: 96.7%.

##### N-(2-((2,4-dimethoxyphenyl)amino)-2-oxoethyl)-2-(1H-indol-3-yl)-2-oxoacetamide (**13i**)

white solid, yield, 82%. ^1^H NMR (400 MHz, DMSO-*d*_6_) δ 12.26 (s, 1H), 9.15 (s, 1H), 8.96 (t, *J* = 6.4 Hz, 1H), 8.80 (d, *J* = 2.8 Hz, 1H), 8.24 (m, 1H), 7.77 (d, *J* = 8.8 Hz, 1H), 7.54 (dd, *J* = 5.0, 1.5 Hz, 1H), 7.29 ~ 7.25 (m, 2H), 6.62 (d, *J* = 2.4 Hz, 1H), 6.50 (dd, *J* = 8.8, 2.4 Hz, 1H), 4.06 (d, *J* = 6.0 Hz, 2H), 3.80 (s, 3H), 3.74 (s, 3H). ^13^C NMR (100 MHz, DMSO-*d*_6_) δ 166.8, 138.7, 136.3, 126.2, 122.9, 122.6, 121.3, 112.6, 104.1, 98.8, 55.7, 55.3, 42.6. ESI–MS: *m/z* calcd. C_20_H_19_N_3_O_5_ [M–H]^+^ 380.3, found 380.1. HPLC purity: 98.3%.

##### 2-(1H-indol-3-yl)-N-(2-((3-methoxyphenyl)amino)-2-oxoethyl)-2-oxoacetamide (**13j**)

white solid, yield, 92%. ^1^H NMR (400 MHz, DMSO-*d*_6_) δ 12.26 (s, 1H), 10.08 (s, 1H), 8.99(t, *J* = 6.1 Hz,1H), 8.70 (s, 1H), 8.27 (td, *J* = 4.1, 2.2 Hz, 1H), 7.55 (m, 1H), 7.30 ~ 7.12 (m, 5H), 6.64 (ddd, *J* = 8.3, 2.5, 1.0 Hz, 1H), 4.05 (d, *J* = 6.0 Hz, 2H), 3.73 (s, 3H). ^13^C NMR (100 MHz, DMSO-*d*_6_) δ 181.5, 167.1, 163.8, 159.5, 140.0, 138.7, 136.3, 129.6, 126.2, 123.5, 122.6, 121.3, 112.6, 112.3, 111.5, 108.7, 105.0, 54.9, 42.6. ESI–MS: *m/z* calcd. C_19_H_17_N_3_O_4_ [M–H]^+^ 350.3, found 350.2. HPLC purity: 97.8%.

##### N-(2-((2-fluorophenyl)amino)-2-oxoethyl)-2-(1H-indol-3-yl)-2-oxoacetamide (**13k**)

white solid, yield, 83%. ^1^H NMR (400 MHz, DMSO-*d*_6_) δ 12.28 (s, 1H), 9.92 (s, 1H), 8.98 ~ 8.93 (m, 1H), 8.82 (d, *J* = 2.1 Hz, 1H), 8.34–8.19 (m, 1H), 7.94 ~ 7.88 (m, 1H), 7.55 (dt, *J* = 6.7, 2.1 Hz, 1H), 7.28 (dt, *J* = 7.4, 2.3 Hz, 3H), 7.17 (ddt, *J* = 7.0, 4.3, 2.2 Hz, 2H), 4.13 (d, *J* = 6.2 Hz, 2H). ^13^C NMR (100 MHz, DMSO-*d*_6_) δ 181.6, 167.6, 163.9, 138.7, 136.3, 126.2, 125.8, 125.4, 124.4, 124.1, 123.5, 122.6, 121.3, 115.6, 115.5, 112.6, 112.3, 42.4. ESI–MS: *m/z* calcd. C_18_H_14_ClN_3_O_3_ [M–H]^+^ 338.1, found 338.2. HPLC purity: 97.3%.

#### General procedure for the synthesis of **17a–d**

Carbamates **16a–d** (0.3 mmol), indole-3-glyoxyl chloride **7** (54.07 mg), and K_2_CO_3_ (53.90 mg, as catalysis) were mixed and reacted at 25 °C for 8 h. The resulting products **17a–d** were separated by silica gel CC using petroleum ether/EtOAc (v/v, 1/1 ~ 1/2) as eluents.

##### 2-(2-(1H-indol-3-yl)-N-methyl-2-oxoacetamido)ethyl m-tolylcarbamate (**17a**)

white solid, yield, 81%.^1^H NMR (400 MHz, DMSO-*d*_6_) δ 12.39 (s, 1H), 8.46 (q, *J* = 3.4 Hz, 1H), 8.21 ~ 8.13 (m, 2H), 7.53 (d, *J* = 8.1 Hz, 1H), 7.28 (q, *J* = 6.9 Hz, 2H), 7.20 (d, *J* = 7.6 Hz, 2H), 7.03 (d, *J* = 8.0 Hz, 1H), 6.71 (t, *J* = 7.9 Hz, 1H), 4.47 (t, *J* = 5.6 Hz, 2H), 3.73 (t, *J* = 9.2 Hz, 2H), 3.03 (s, 3H), 2.15 (s, 3H). ^13^C NMR (100 MHz, DMSO-*d*_6_) δ 178.6, 163.3, 155.5, 140.2, 138.5, 137.2, 136.7, 127.9, 125.5, 123.8, 122.8, 122.5, 121.1, 120.6, 117.2, 112.7, 63.4, 46.8, 35.2, 21.1. ESI–MS: *m/z* calcd. C_21_H_21_N_3_O_4_ [M–H]^+^ 378.1, found 378.1. HPLC purity: 98.1%.

##### 2-(2-(1H-indol-3-yl)-N-methyl-2-oxoacetamido)ethyl (4-ethylphenyl)carbamate (**17b**)

white solid, yield, 83%. ^1^H NMR (400 MHz, DMSO-*d*_6_) δ 12.40 (s, 1H), 8.46 (d, *J* = 3.3 Hz, 1H), 8.22 ~ 8.13 (m, 2H), 7.54 (dd, *J* = 4.3, 2.5 Hz, 1H), 7.29 (dq, *J* = 9.2, 4.2, 3.2 Hz, 4H), 6.99 (dd, *J* = 8.5, 3.6 Hz, 2H), 4.48 (t, *J* = 5.0 Hz, 2H), 3.73 (t, *J* = 5.0 Hz, 2H), 3.35 (s, 2H), 3.03 (d, *J* = 3.7 Hz, 3H), 1.12 (s, 3H). ^13^C NMR (100 MHz, DMSO-*d*_6_) δ 178.6, 163.4, 155.5, 138.5, 137.9, 137.1, 136.7, 127.3, 125.5, 123.8, 122.8, 121.1, 120.2, 112.7, 112.4, 63.5, 46.8, 35.2, 27.5, 15.7. ESI–MS: *m/z* calcd. C_22_H_23_N_3_O_4_ [M–H]^+^ 392.4, found 392.3. HPLC purity: 98.3%.

##### 2-(2-(1H-indol-3-yl)-N-methyl-2-oxoacetamido)ethyl o-tolylcarbamate (**17c**)

white solid, yield, 87%. ^1^H NMR (400 MHz, DMSO-*d*_6_) δ 12.40 (s, 1H), 8.45 (d, *J* = 2.8 Hz, 1H), 8.18 (dd, *J* = 6.4, 2.8 Hz, 1H), 7.82 (s, 1H), 7.57 ~ 7.53 (m, 1H), 7.32 ~ 7.27 (m, 2H), 7.13 (dt, *J* = 5.6, 2.4 Hz, 2H), 7.01 (td, *J* = 6.4, 2.0 Hz, 2H), 4.49 (t, *J* = 5.4 Hz, 2H), 3.73 (t, *J* = 5.4 Hz, 2H), 3.04 (s, 3H), 2.11 (s, 3H). ^13^C NMR (100 MHz, DMSO-*d*_6_) δ 178.7, 163.4, 155.9, 138.4, 137.9, 136.7, 133.1, 129.9, 126.1, 125.6, 125.5, 124.5, 123.8, 122.8, 121.1, 112.7, 112.4, 63.4, 46.9, 35.1, 17.7. ESI–MS: *m/z* calcd. C_21_H_21_N_3_O_4_ [M–H]^+^ 378.4, found 378.2. HPLC purity: 99.5%.

##### 2-(2-(1H-indol-3-yl)-N-methyl-2-oxoacetamido)ethyl (3-chlorophenyl)carbamate (**17d**)

white solid, yield, 83%. ^1^H NMR (400 MHz, DMSO-*d*_6_) δ 12.40 (s, 1H), 8.46(m, 2H), 8.17 (m, 1H), 7.59 (d, *J* = 2.1 Hz, 1H), 7.37 (m, 1H), 7.32 ~ 7.18 (m, 4H), 6.93 (dd, *J* = 8.0, 2.1 Hz, 1H), 4.48 (t, *J* = 5.4 Hz, 2H), 3.73 (t,* J* = 5.4 Hz, 2H), 3.04 (s, 3H). ^13^C NMR (100 MHz, DMSO-*d*_6_) δ 178.5, 163.3, 155.1, 141.9, 138.4, 132.6, 129.7, 125.5, 123.8, 122.8, 121.3, 121.1, 119.1, 117.9, 112.7, 112.3, 63.3, 46.9, 35.2. ESI–MS: *m/z* calcd. C_20_H_18_ClN_3_O_4_ [M–H]^+^ 398.7,found 398.2. HPLC purity: 98.1%.

### BChE inhibition assays

BChE inhibitory activities were determined according to a procedure reported in our previous study^17^. Galantamine and tacrine (hydrochloride) were used as positive controls.

### Enzyme kinetics studies

Kinetic studies were performed in same method as described in BChE inhibition assays. The substrate (BTCI) at five concentrations (from 0.05 to 0.8 mM) was determined for **8c** at different times to reveal its inhibition model, and reciprocal plots of 1/V versus 1/[S]. The kinetic parameters and apparent inhibition constants were calculated by the “Enzyme kinetics” module of Prism.

### Molecular docking

The binding mode of **8c** in the active sites of BChE was illustrated by using the Glide module of Schrödinger (Schrödinger LLC, NY, USA). The X-ray crystal structure of BChE (PDB ID: 4TPK) was downloaded from the RCSB Protein Date Bank (http://www.rcsb.org/). First, the protein receptor was pretreated with the protein preparation module of Schrödinger to retain A chain and the original ligand of 4TPK located in the active cavity of A chain, while the remaining redundant ligand molecules, B chain, and solvents were not retained. Then, the receptor grids were prepared by Maestro to define the active site of the protein through the co-crystallized ligand and it was excluded from grid generation. The box length in X, Y and Z was 10 Å. The processing of inhibitor **8c** was also performed on Schrödinger, using the Ligprep module to generate various 3D structures of **8c**. Finally, run the docking module, set the scaling factor and partial charge cut-off value as 0.8 and 0.15, respectively, and used the default parameters for other parameters.

### Molecular dynamics simulation

Analyzing the behavior and motion of the BChE-**8c** complex in a simulated environment could be a good way to grasp the stability, flexibility, and binding energy of **8c** to BChE. Therefore, molecular dynamics simulation of BChE-**8c** was performed with GROMACS 2019.5. GROMOS96 force field was used to generate the topology of the protein, while the topology of **8c** was obtained from the ATB website at https://atb.uq.edu.au/index.py. When uploading, the ligand molecule was simultaneously charge corrected by selecting its corresponding charge number according to the charge of the molecule itself. The minimum distance between the protein atoms and the cell wall was set to 1 Å and cubic cell was used in the entire MD simulation process to maintain the periodic boundary conditions. The solvents were added to the system, and the solvent type was SPC solvent model and the solvent type was water molecule. Since the processed system might not be in an electrically neutral state, the charge of the system was balanced by adding counter ions such as Cl^−^ or Na^+^ to the plasma to achieve a physiologically neutral state. Performing energy minimization to eliminate stereo conflicts, create indexes for thermal coupling groups and modify mdp files, and subsequent location restrictions. After minimization, the NVT ensemble was carried out under 300 K, followed by the NPT ensemble. 50 ns molecular dynamics simulation was carried out for a well-balanced system with a time step of 0.002 ns.

### In silico ADME prediction

Considering the ADME properties of a drug candidate is quite important, so, the QikProp module (Schrödinger) was adapted for ADME prediction of **8c** in silico. The various properties of **8c** were obtained, such as partition coefficient (QPlogp octanol/water), water solubility, percentage of human oral absorption, and hydrogen bond donor/acceptor. It is worth noting that molecular preprocessing of **8c** using the Ligprep module was required before performing QikProp calculations.

### Statistical analysis

All bioassays were carried out in triplicates. Data obtained were presented as mean and standard deviation values (S.E.M.). The IC_50_ values were calculated with GraphPad.

## Supplementary Information

Supplementary Information.

## Data Availability

All data is provided in the manuscript or Supplementary Material.

## Abbreviations

ACh: Acetylcholine
AChE: Acetylcholinesterase
AD: Alzheimer’s disease
BChE: Butyrylcholinesterase
CAS: Catalytic active site
CNS: Central nervous system
hAChE: Human acetylcholinesterase
hBChE: Human butyrylcholinesterase
HOA: Human oral absorption
MD: Molecular dynamics
PAS: Peripheral anionic site
Rg: Radius of gyration
RMSD: Root mean squared deviation
RMSF: Root mean squared fluctuation
SAR: Structure–activity relationship
SASA: Solvent accessible surface area
TMS: Tetramethylsilane
