# Supplementary material for: Synthesis and biological evaluation of substituted acetamide derivatives as potential butyrylcholinestrase inhibitors
Source: Sci Rep. 2023 Mar 25;13:4877. doi: 10.1038/s41598-023-31849-5 (PMC10039877; doi:10.1038/s41598-023-31849-5)

**SUPPLEMENTARY MATERIAL**

**Synthesis and biological evaluation of substituted acetamide derivatives as potential butyrylcholinestrase inhibitors**

Dehong Yu ^1^, Can Yang ^1^, Yi Liu^1^, Tao Lu^1^, lizi Li ^1^, Gang Chen ^2,3^, Zerong Liu ^2,3^, and Yanfang Li*^1^

^1^ School of Chemical Engineering, Sichuan University, Chengdu, 610065, China

^2^ Central Nervous System Drug Key Laboratory of Sichuan Province, Luzhou, 646106, China

^3^ Sichuan Credit Pharmaceutical CO., Ltd. Luzhou, 646106, China

^*^Corresponding author：E-mail address: lyf471@vip.163.com

**List of Contents**

**Table S1** HPLC analysis data of 19 compounds**.**

**Figure S1**. The representative ^1^H NMR, ^13^C NMR, ESI-MS spectra and HPLC chromatogram of compounds **8a-d,** **13a-k,** and **17a-d.**

**Table S1.** HPLC analysis data of compounds **8a-d, 13a-k** and **17a-d.**

The purities of compounds were determined by the methods shown in following table.

The peak aera was determined according UV (254 nm) or ELSD detector.

| Equipment | Alltech Modells 201 with a ELSD 6000 detector | | |
| --- | --- | --- | --- |
|  |  |  |  |
| Column | Nacalai tesque COSMOSIL Packed column AR-Ⅱ (4.6 ID × 250 mm, 5μm ) | | |
|  |  |  |  |
| Method | CH_3_OH/ 0.1% formic acid, from 70% (v/v) of CH_3_OH gradient in the beginning to 100%(v/v) of CH_3_OH gradient in 30 minutes | | |
|  |  |  |  |
|  | flow rate: 1.0 mL /min | | |
|  |  |  |  |
|  |  |  |  |
| Results | Compounds | Retention time (min) | Relative purity (%) |
|  | **8a** | 12.6 | 96.5 |
|  | **8b** | 11.3 | 95.8 |
|  | **8c** | 22.7 | 97.0 |
|  | **8d** | 17.2 | 97.1 |
|  | **13a** | 18.7 | 98.8 |
|  | **13b** | 17.0 | 99.0 |
|  | **13c** | 11.2 | 98.9 |
|  | **13d** | 18.0 | 98.4 |
|  | **13e** | 20.6 | 98.1 |
|  | **13f** | 28.9 | 99.1 |
|  | **13g** | 26.5 | 98.6 |
|  | **13h** | 20.7 | 96.7 |
|  | **13i** | 13.2 | 98.3 |
|  | **13j** | 10.8 | 97.8 |
|  | **13k** | 13.2 | 97.3 |
|  | **17a** | 13.8 | 98.1 |
|  | **17b** | 13.9 | 98.3 |
|  | **17c** | 21.0 | 99.5 |
|  | **17d** | 12.6 | 98.1 |
|  |  |  |  |
|  |  |  |  |

**Figure S1**. The ^1^H NMR, ^13^C NMR, ESI-MS spectra and HPLC chromatogram of compounds **8a-d, 13a-k** and **17a-d.**

^1^H NMR spectrum of compound **8a**（400 MHz, DMSO-*d*_6_）


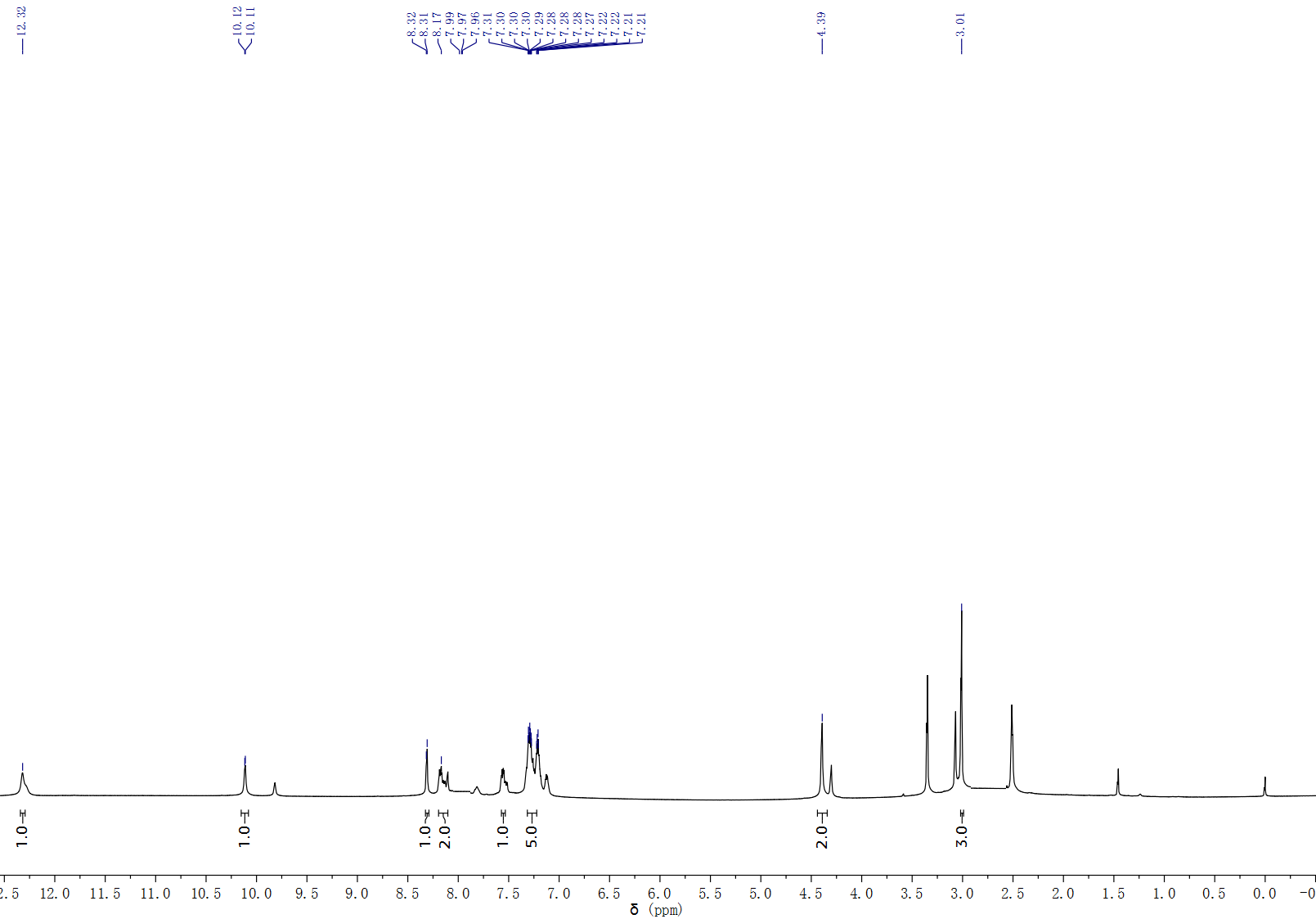


^13^C NMR spectrum of compound **8a**（100 MHz, DMSO-*d*_6_）


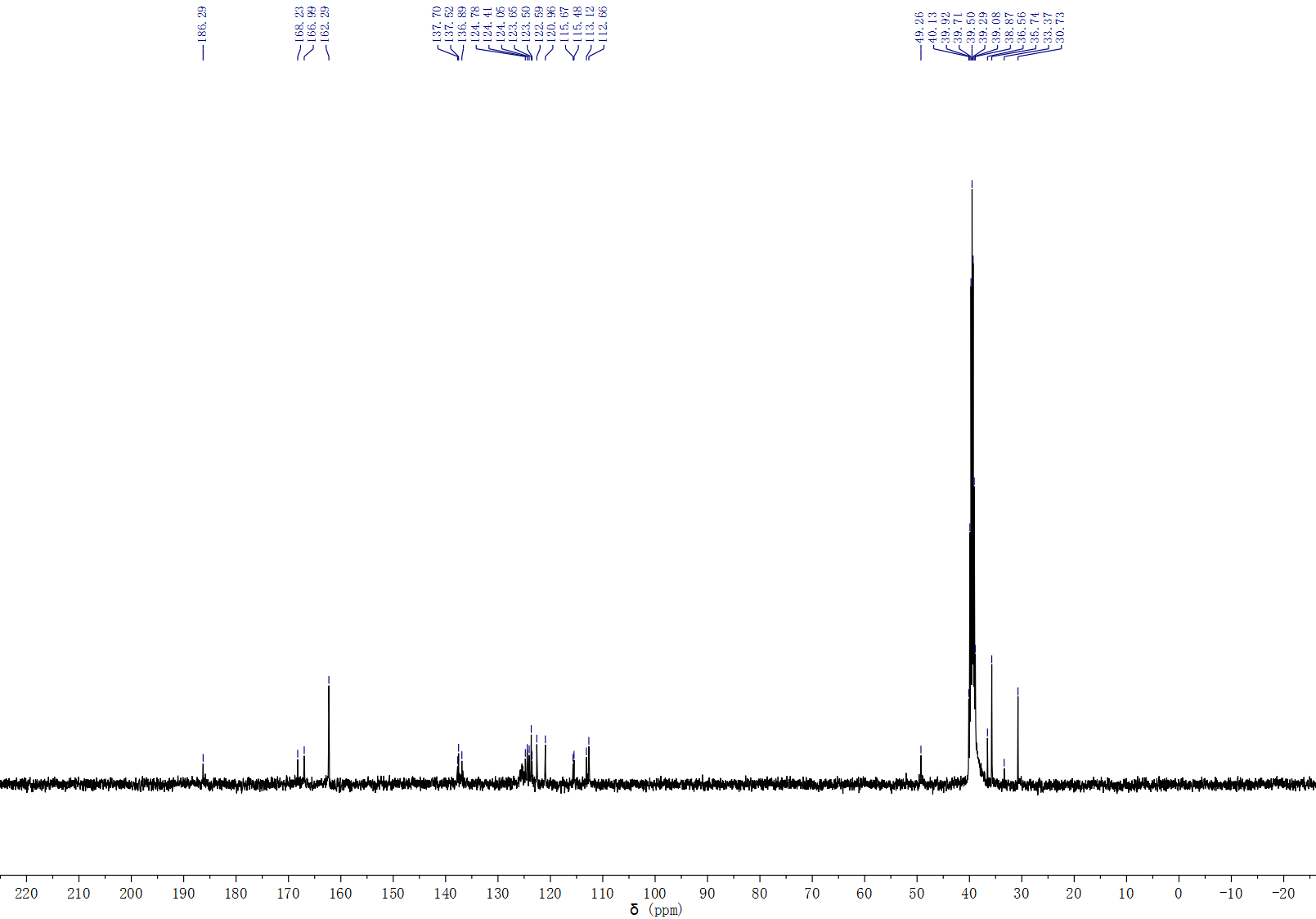


MS(ESI) spectrum of compound **8a**.

HPLC chromatogram of compound **8a**.


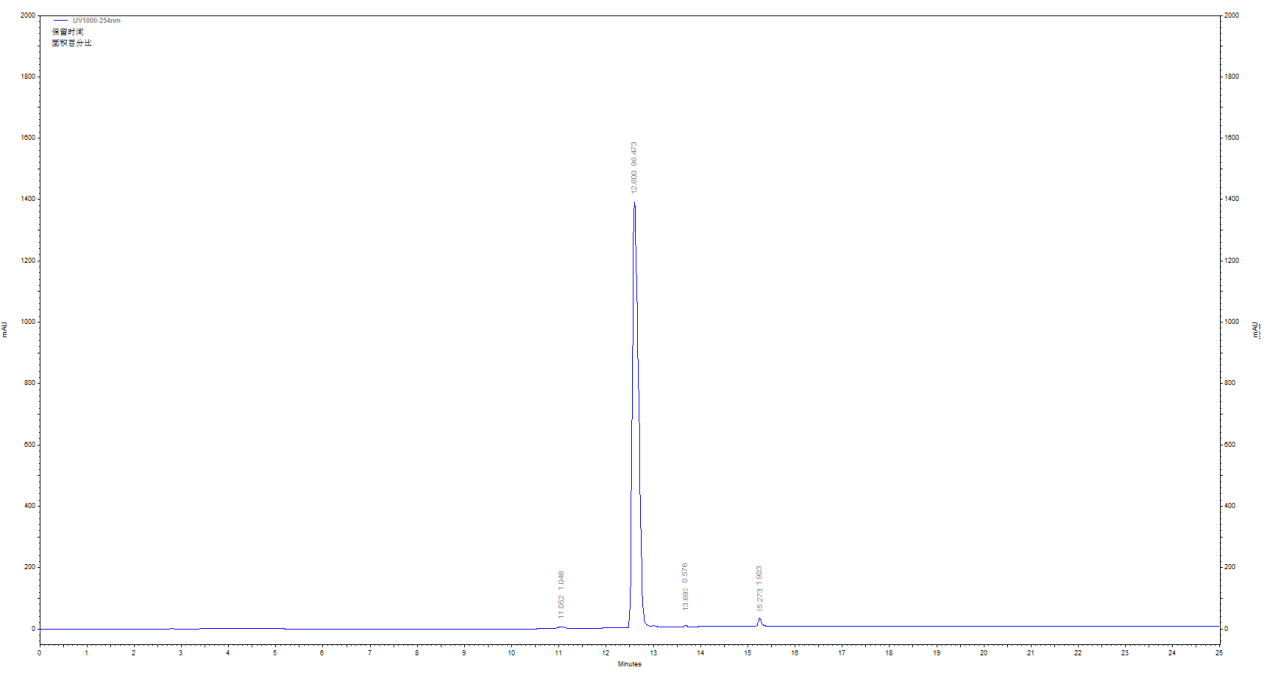


^1^H NMR spectrum of compound **8b**（400 MHz, DMSO-*d*_6_）


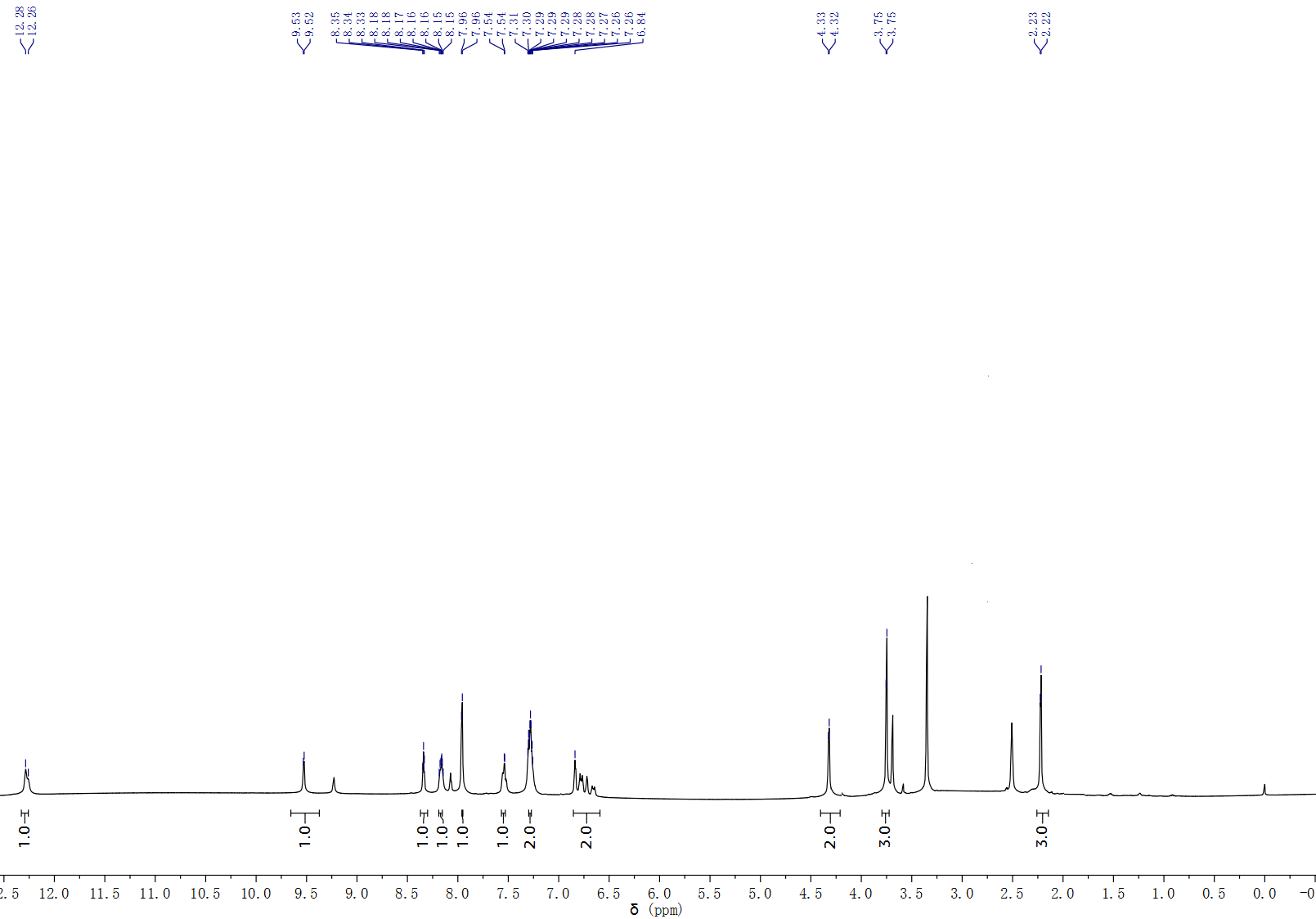


^13^C NMR spectrum of compound **8b**（100 MHz, DMSO-*d*_6_）


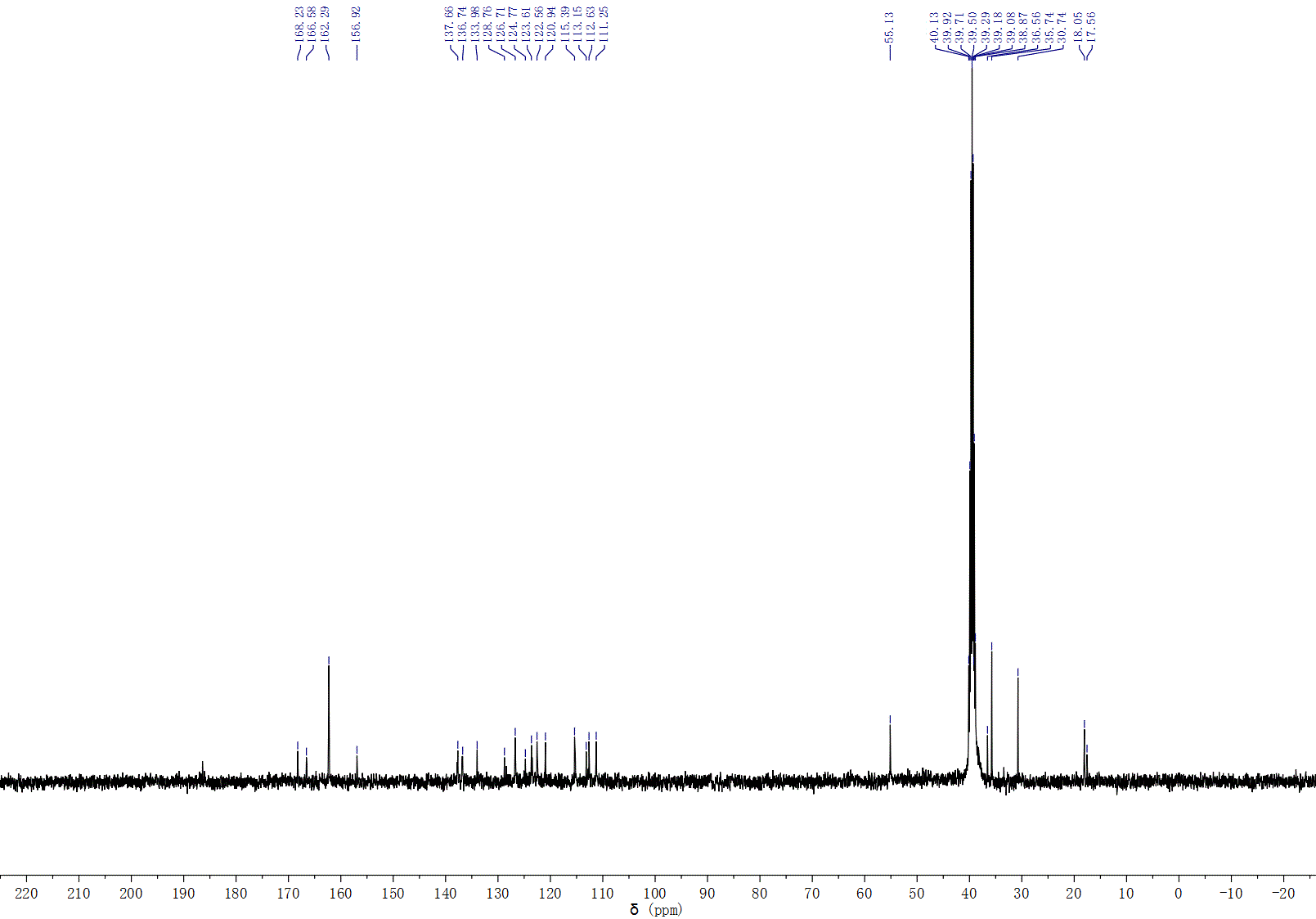


MS(ESI) spectrum of compound **8b**.

HPLC chromatogram of compound **8b**.


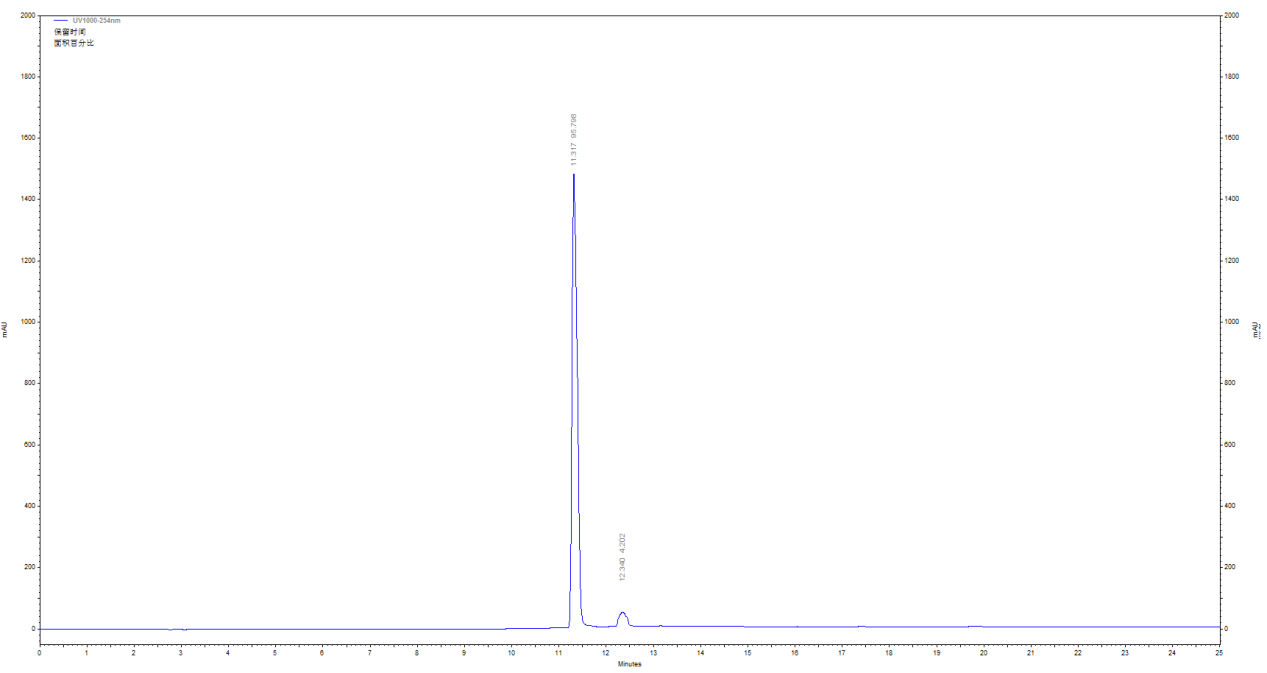


^1^H NMR spectrum of compound **8c**（400 MHz, DMSO-*d*_6_）


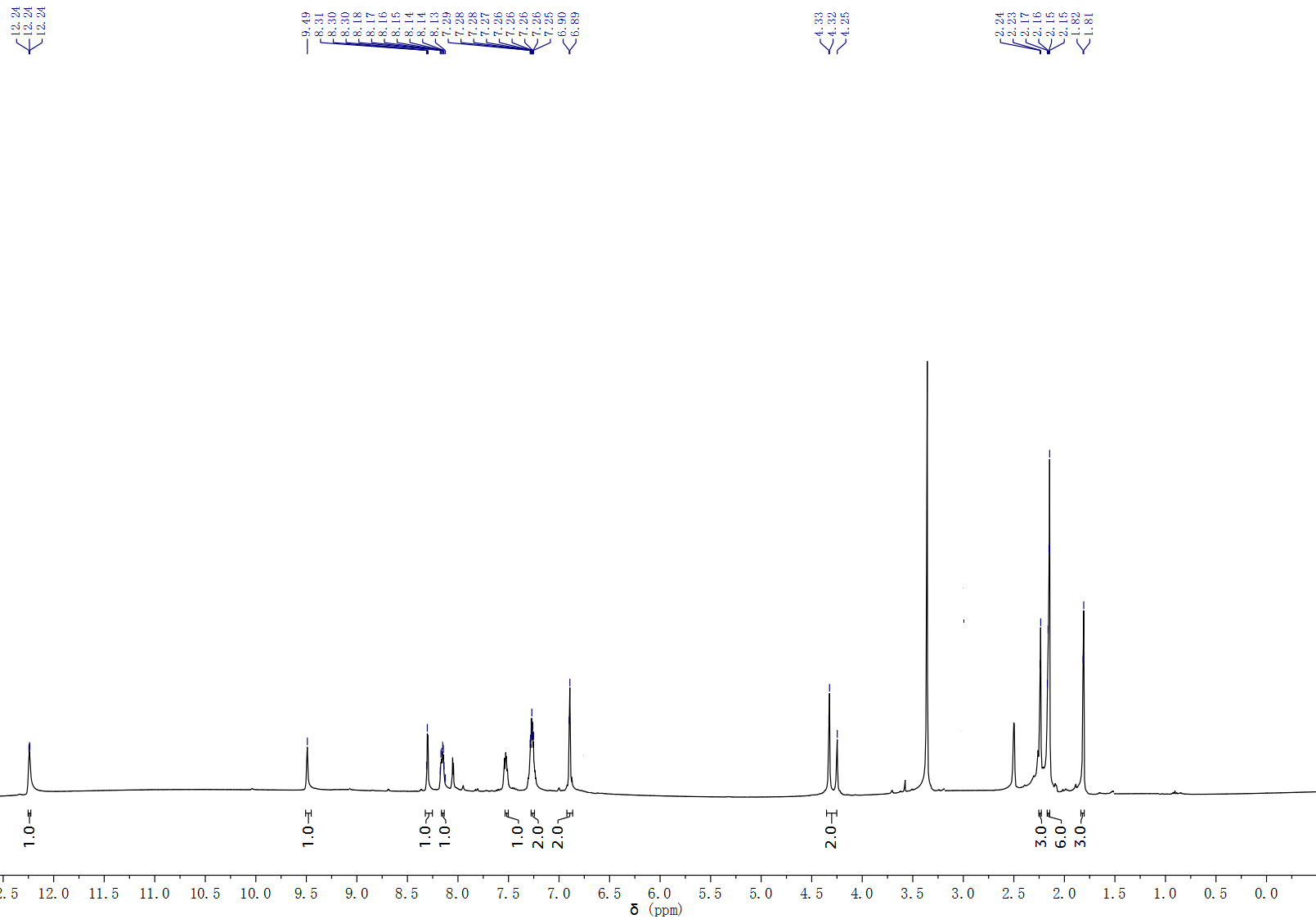


^13^C NMR spectrum of compound **8c**（100 MHz, DMSO-*d*_6_）


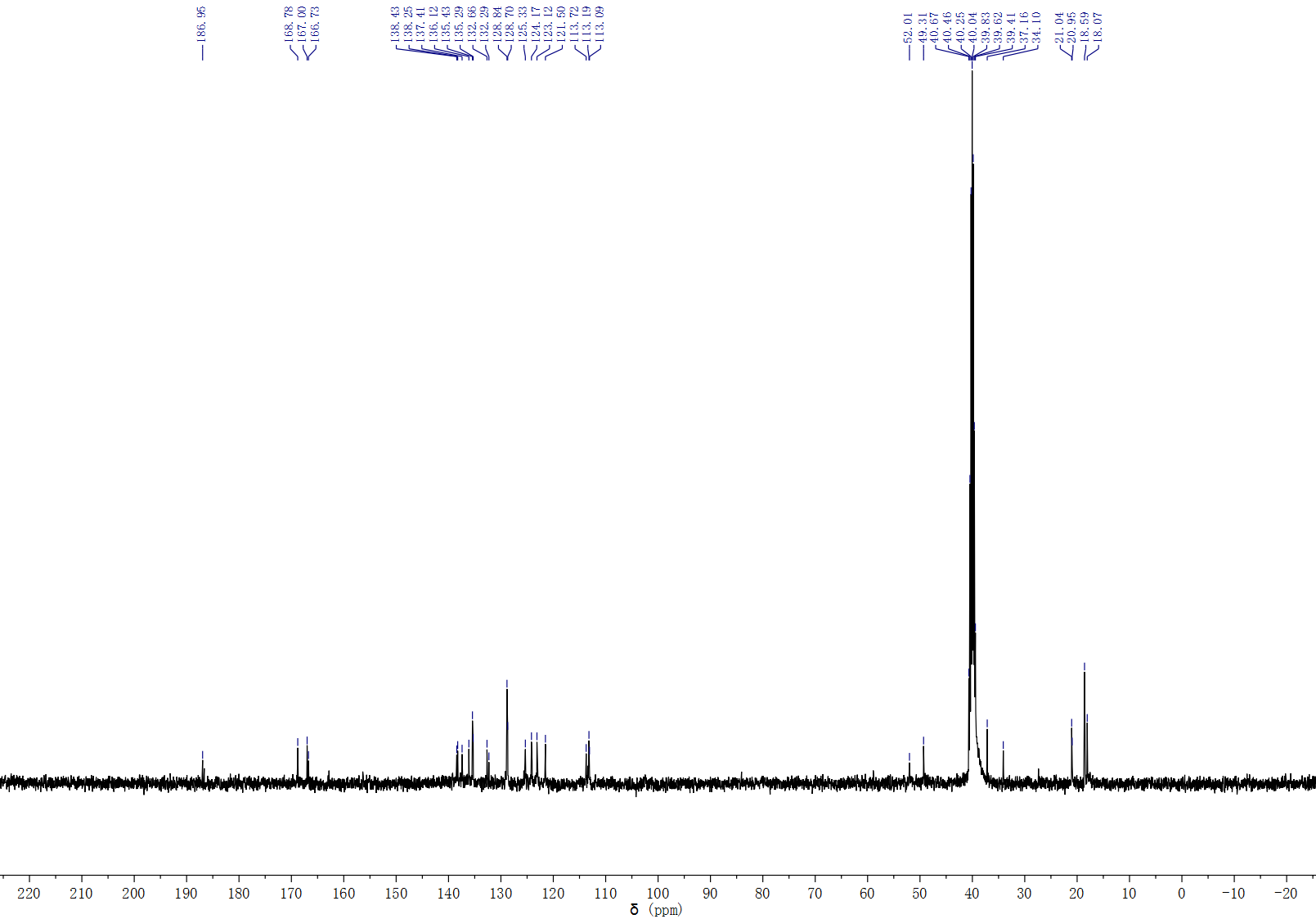


MS(ESI) spectrum of compound **8c**.

HPLC chromatogram of compound **8c**.


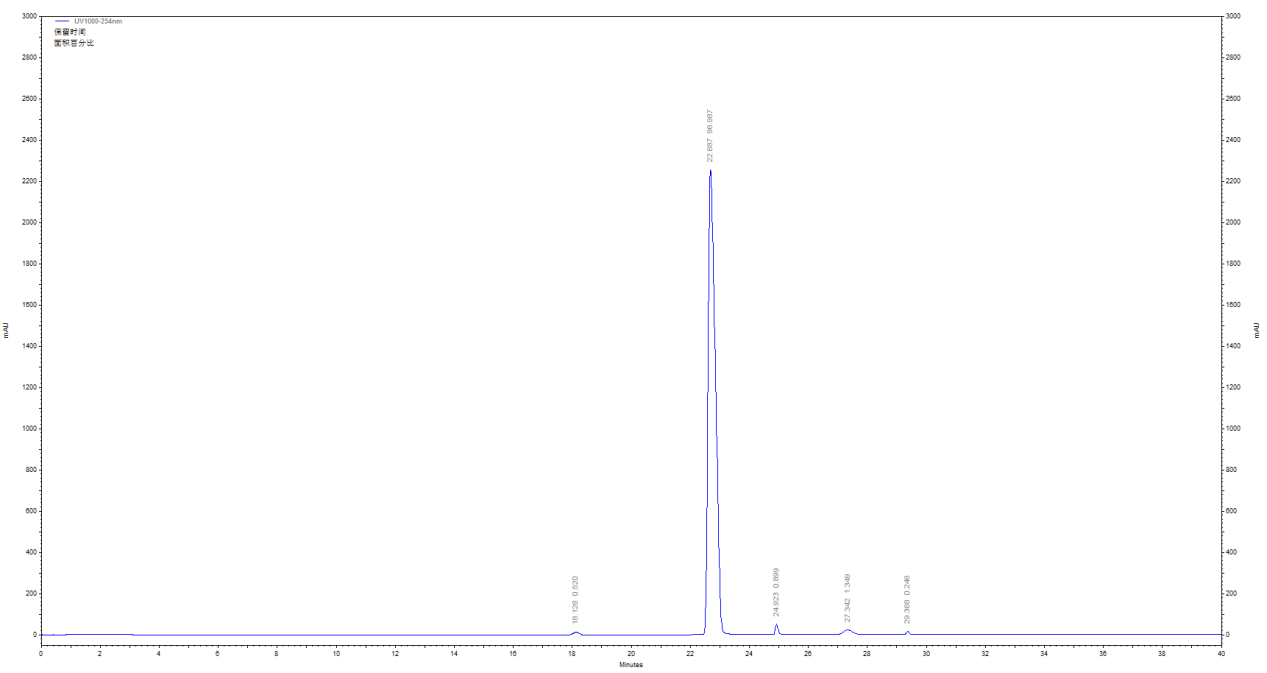


^1^H NMR spectrum of compound **8d**（400 MHz, DMSO-*d*_6_）


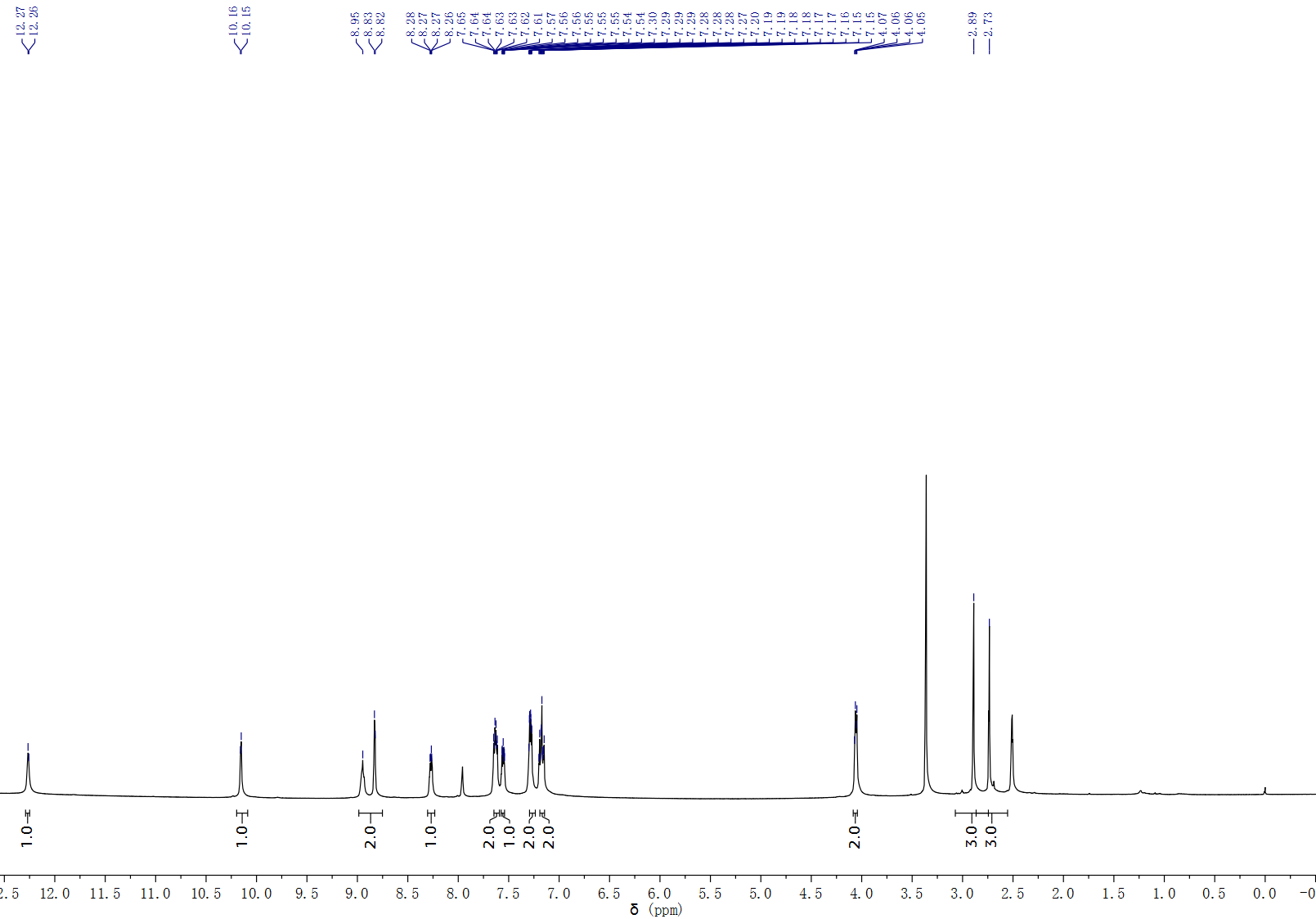


^13^C NMR spectrum of compound **8d**（100 MHz, DMSO-*d*_6_）


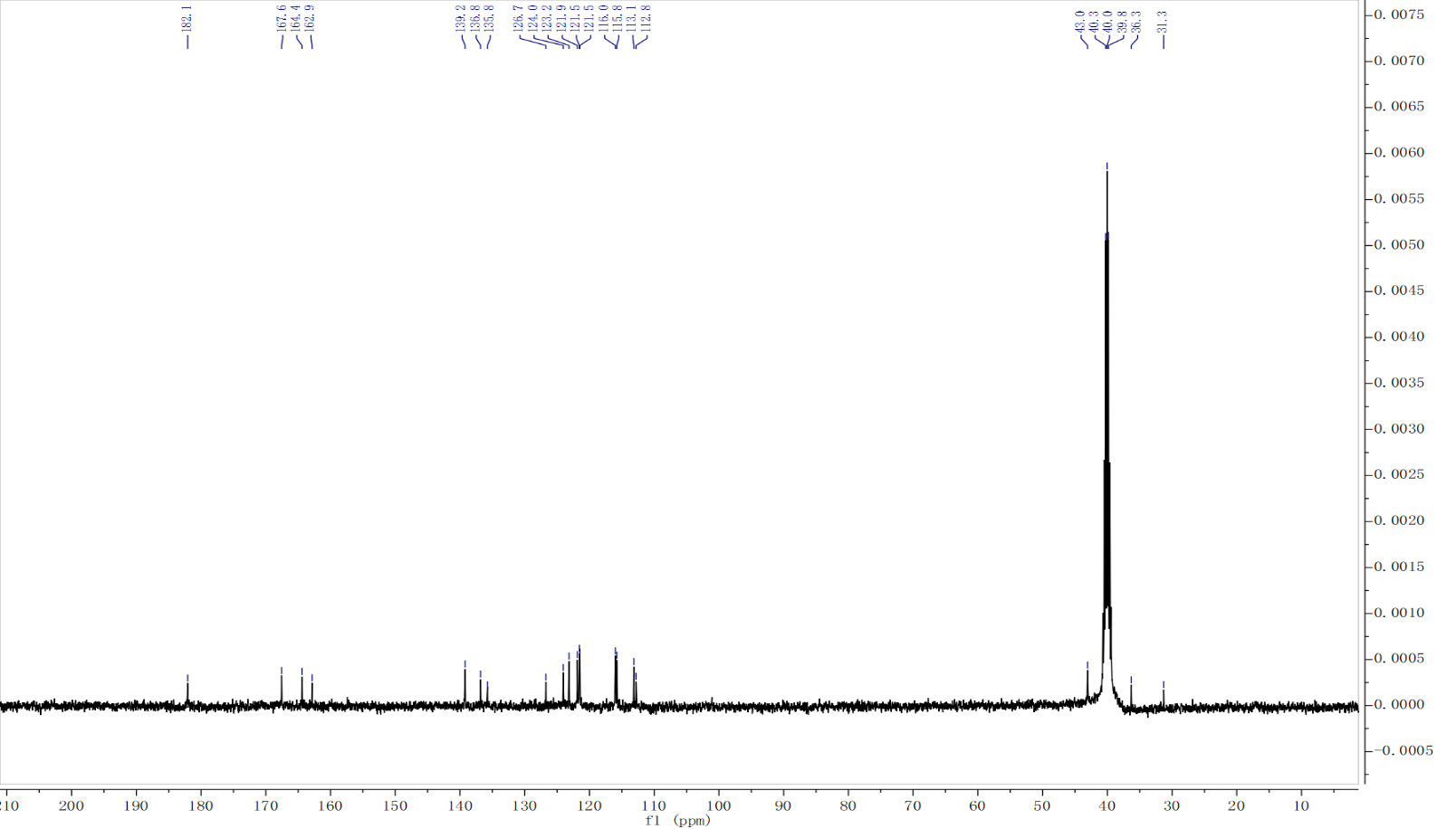


MS(ESI) spectrum of compound **8d**.

HPLC chromatogram of compound **8d**.


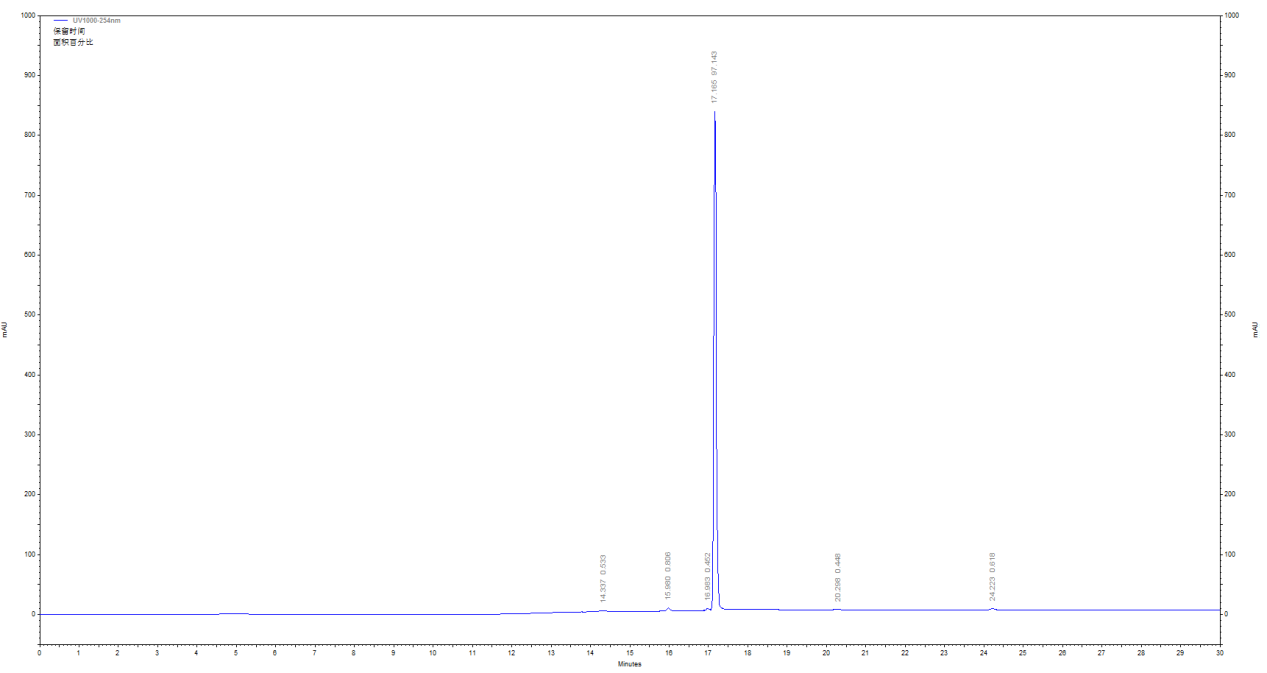


^1^H NMR spectrum of compound **13a**（400 MHz, DMSO-*d*_6_）


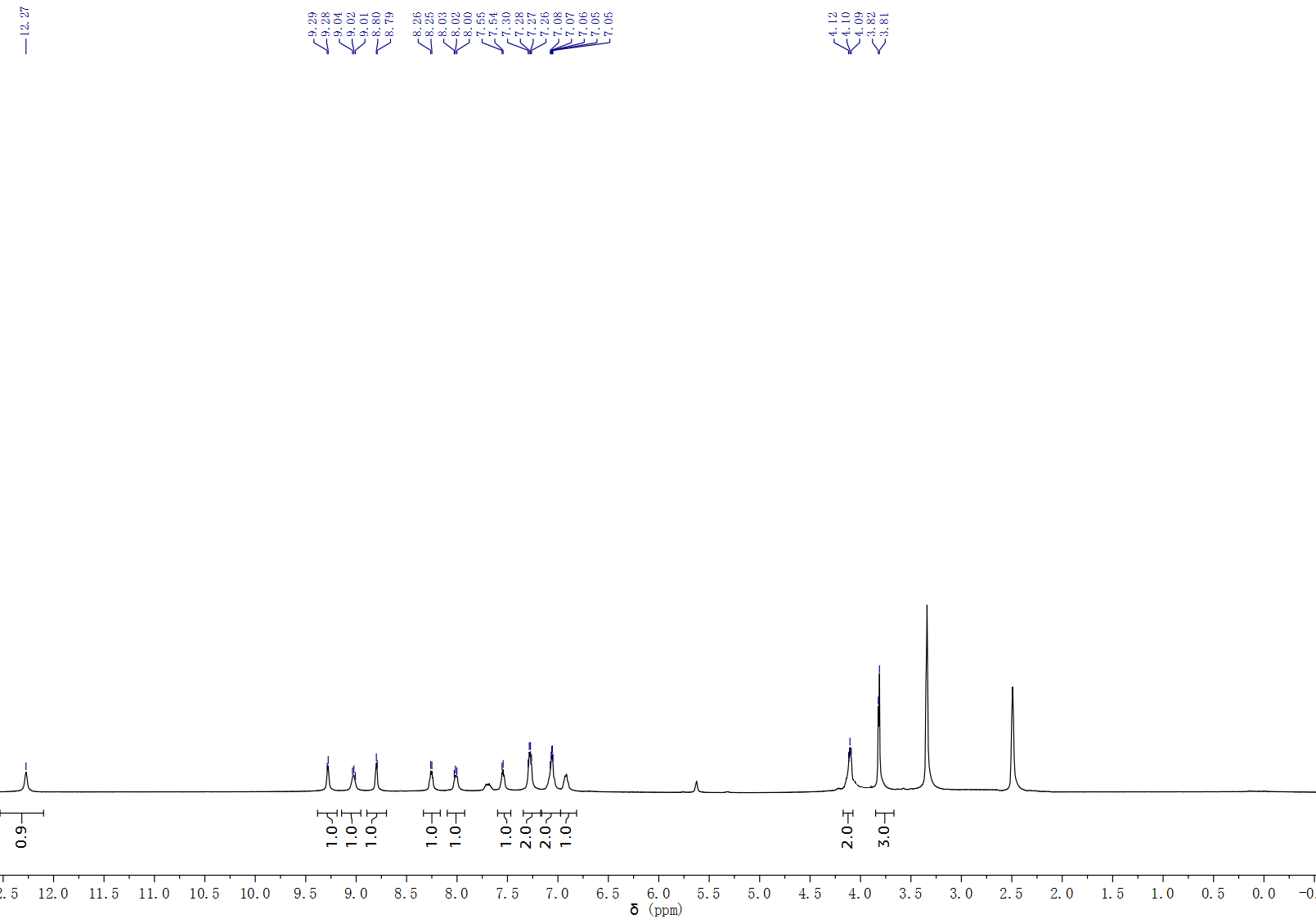


^13^C NMR spectrum of compound **13a**（100 MHz, DMSO-*d*_6_）


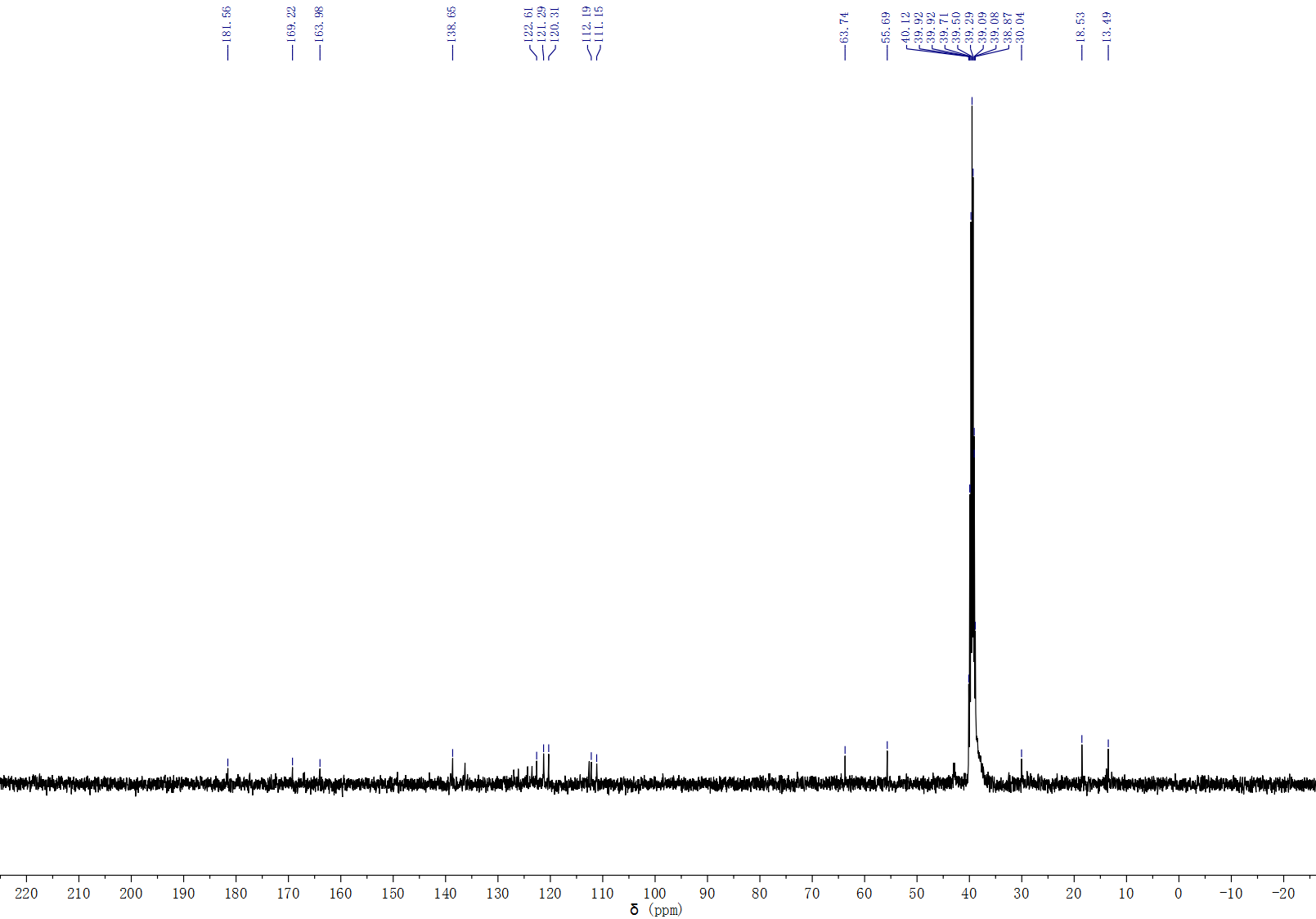


MS(ESI) spectrum of compound **13a**.

HPLC chromatogram of compound **13a**.


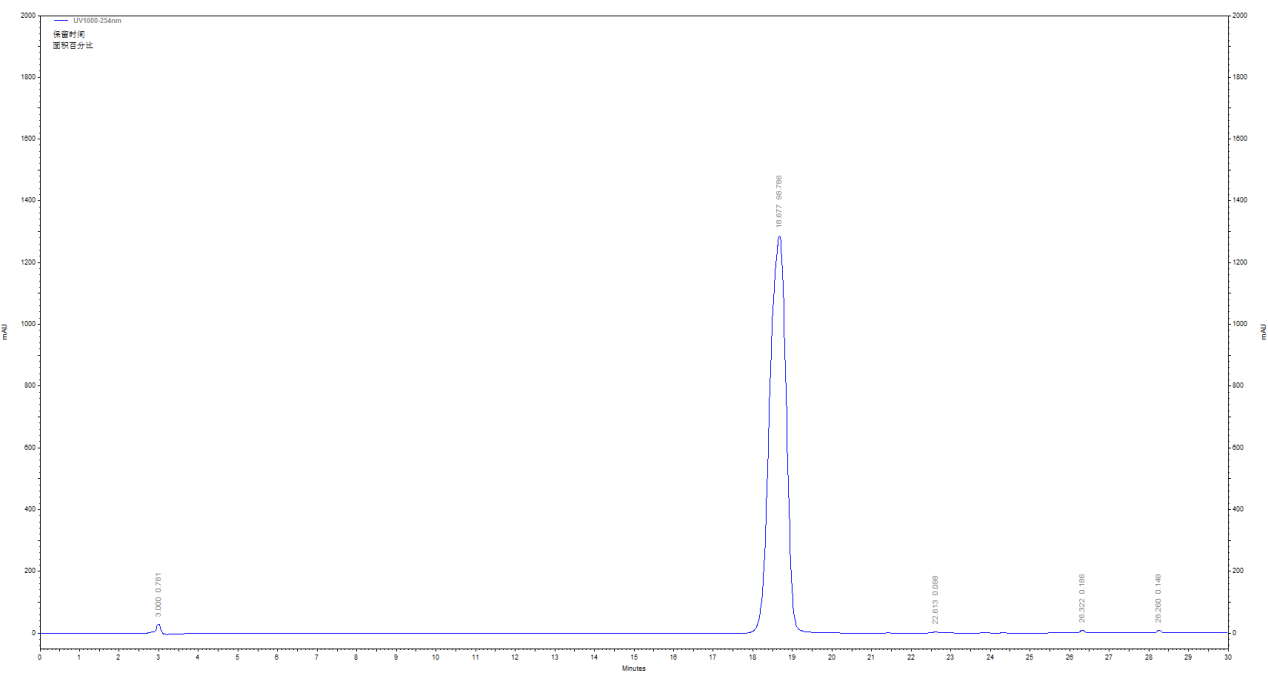


^1^H NMR spectrum of compound **13b**（400 MHz, DMSO-*d*_6_）


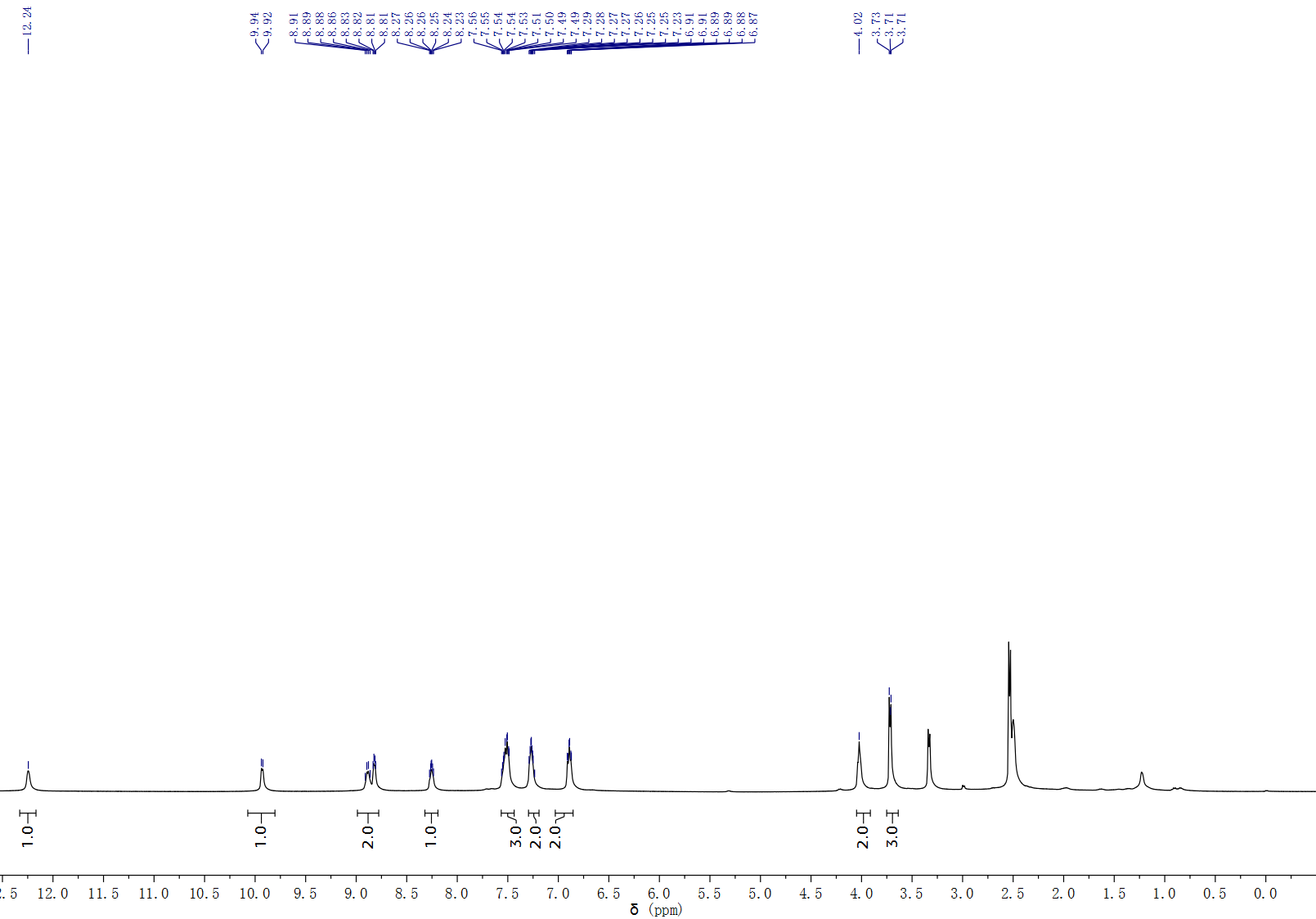


^13^C NMR spectrum of compound **13b**（100 MHz, DMSO-*d*_6_）
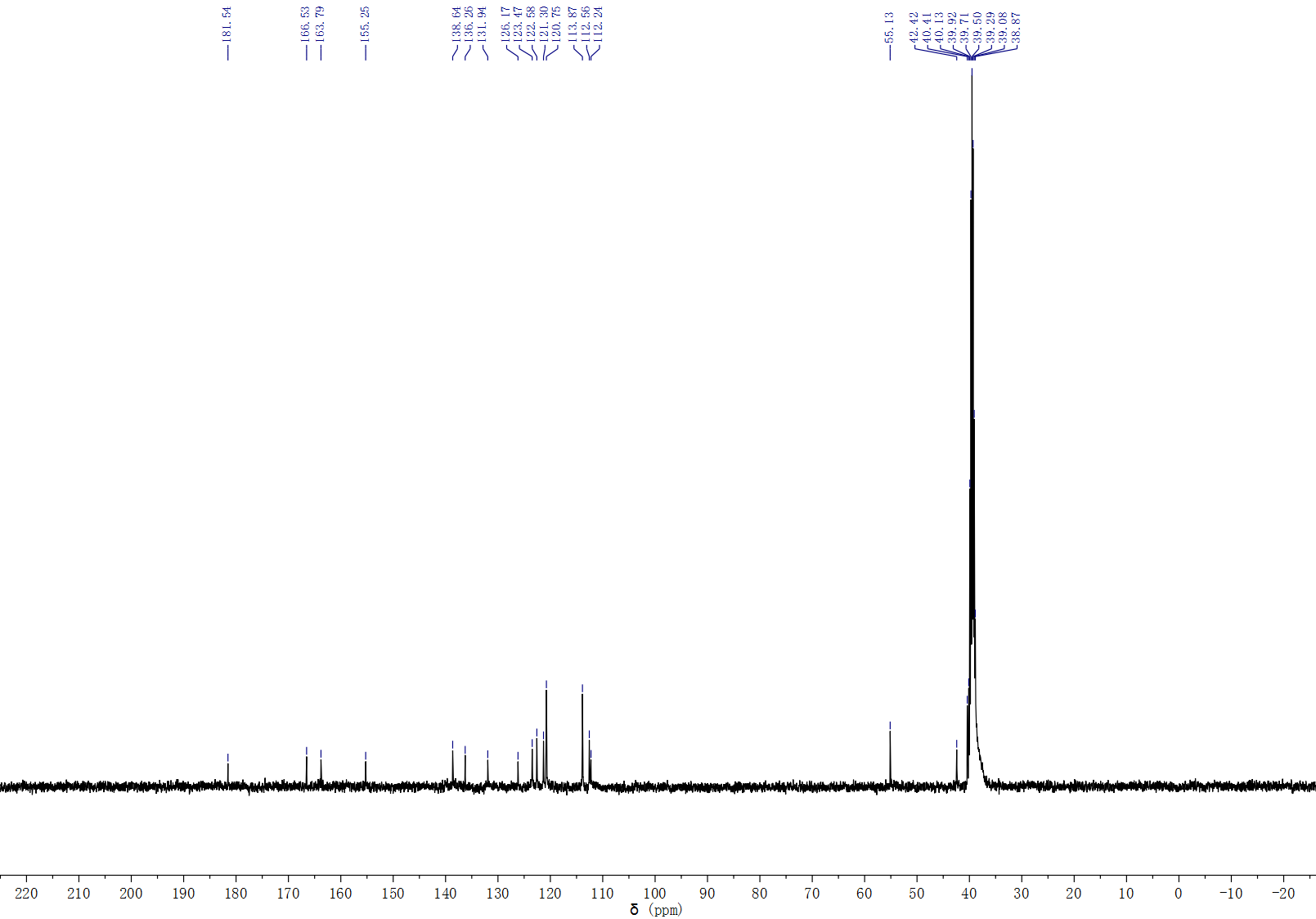


MS(ESI) spectrum of compound **13b**.

HPLC chromatogram of compound **13b**.


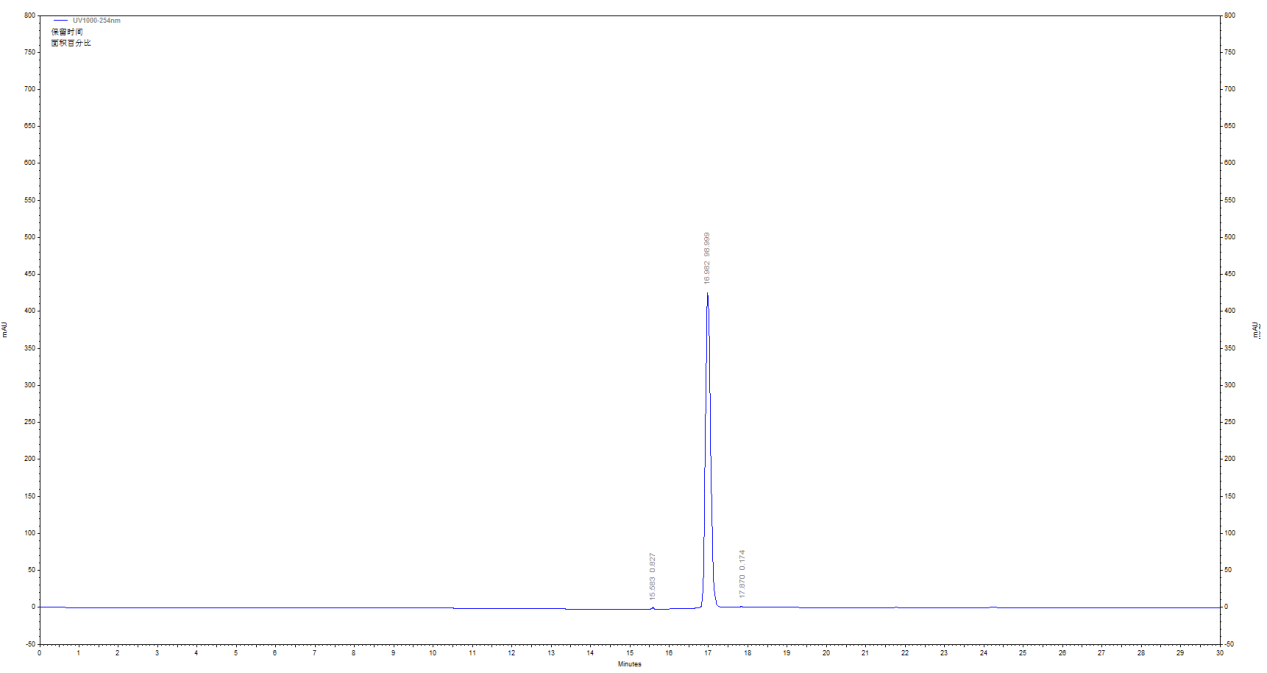


^1^H NMR spectrum of compound **13c**（400 MHz, DMSO-*d*_6_）


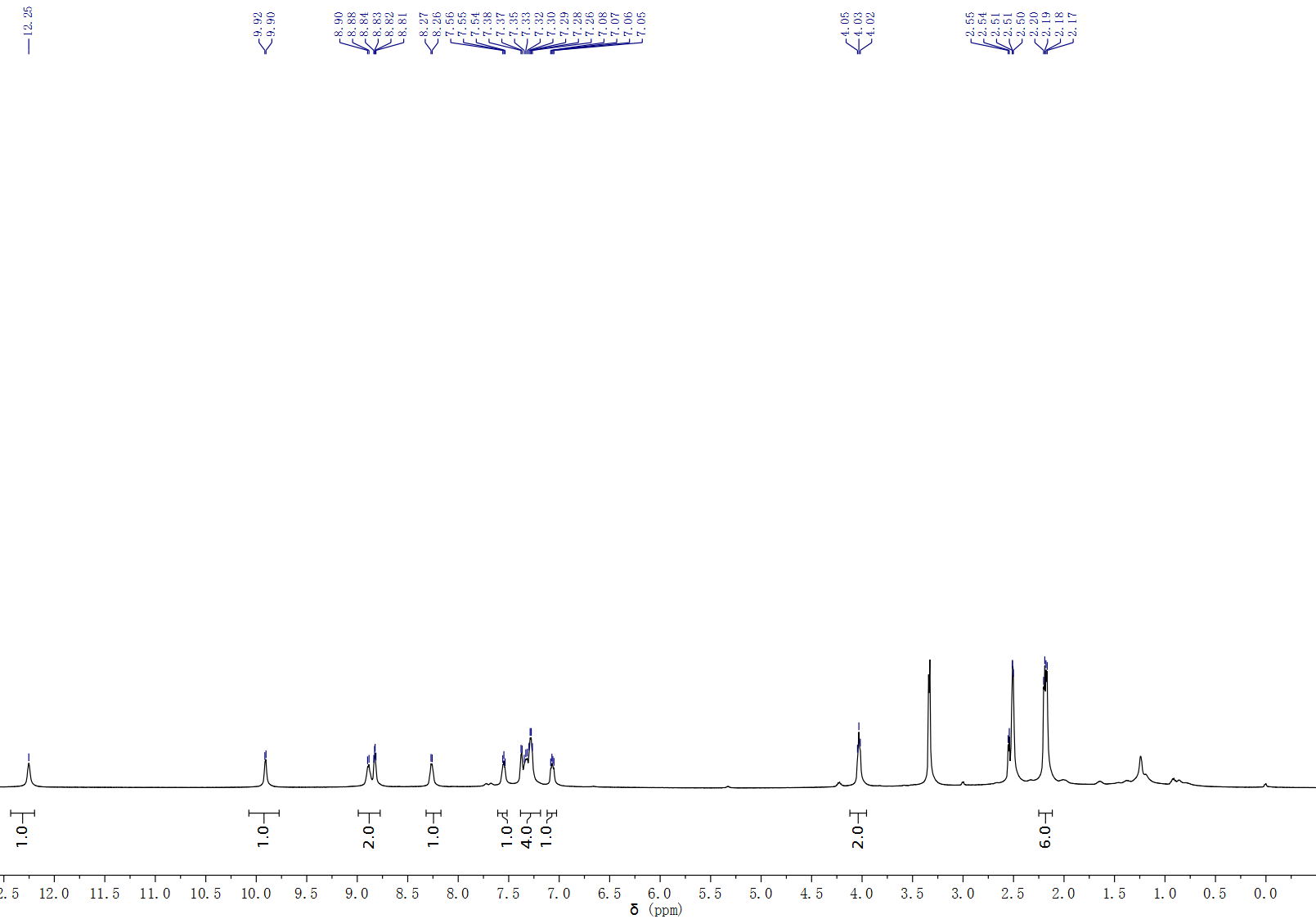


^13^C NMR spectrum of compound **13c**（100 MHz, DMSO-*d*_6_）


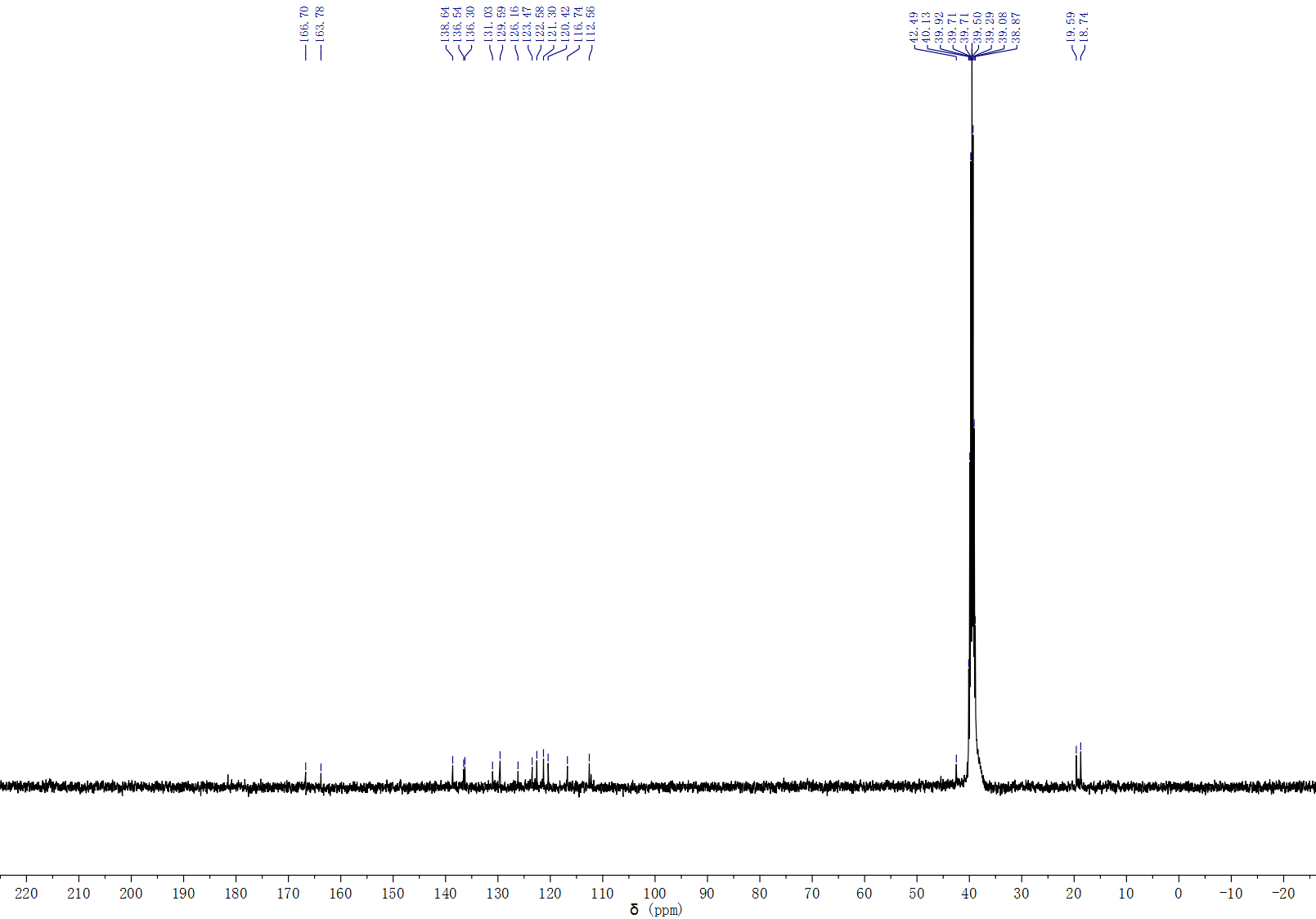


MS(ESI) spectrum of compound **13c**.

HPLC chromatogram of compound **13c**.


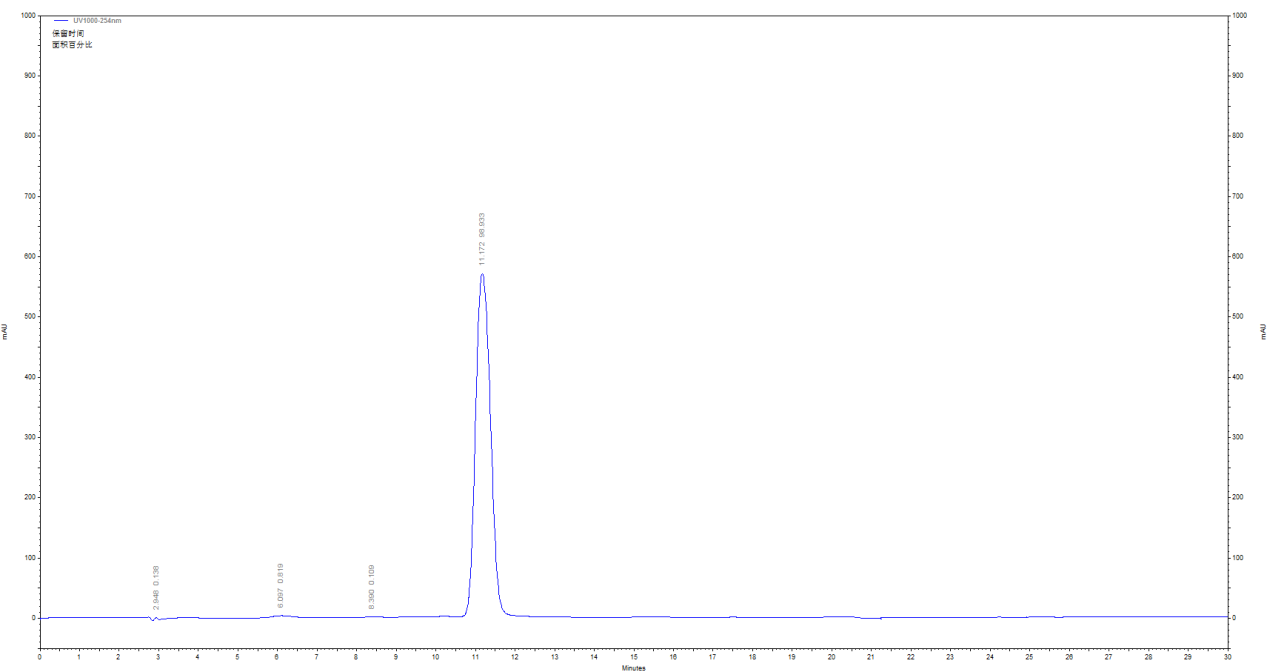


^1^H NMR spectrum of compound **13d**（400 MHz, DMSO-*d*_6_）


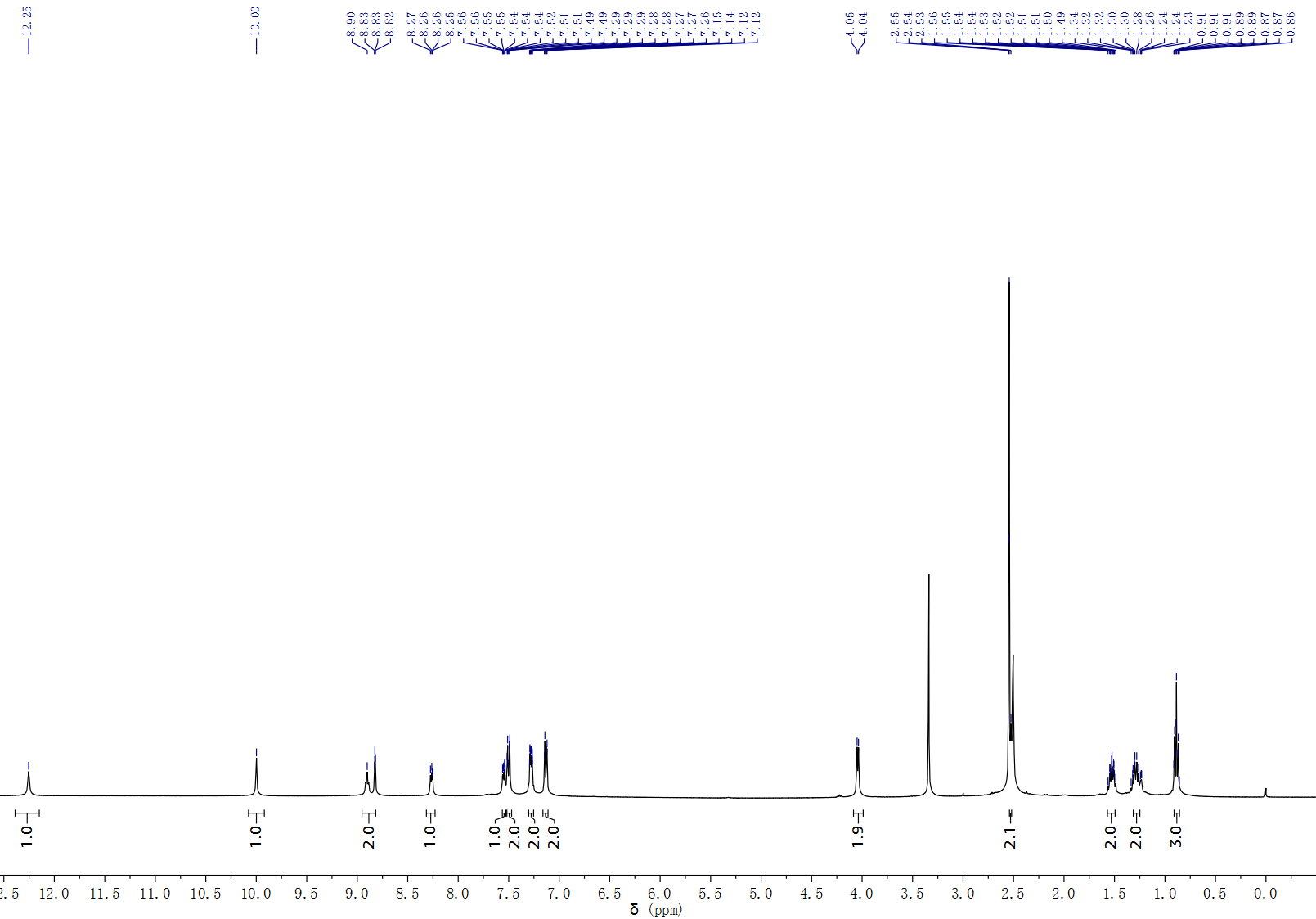


^13^C NMR spectrum of compound **13d**（100 MHz, DMSO-*d*_6_）


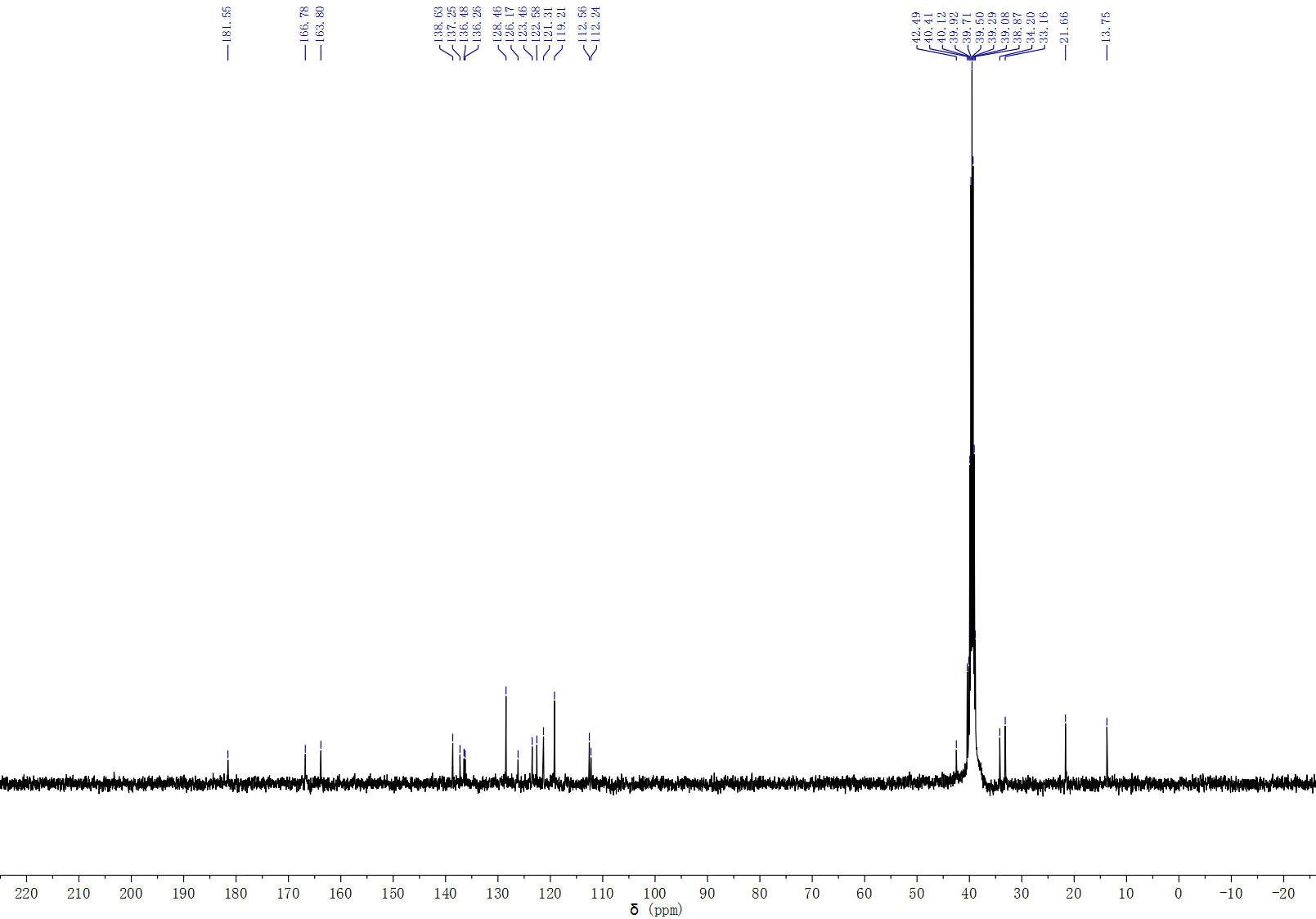


MS(ESI) spectrum of compound **13d**.

HPLC chromatogram of compound **13d**.


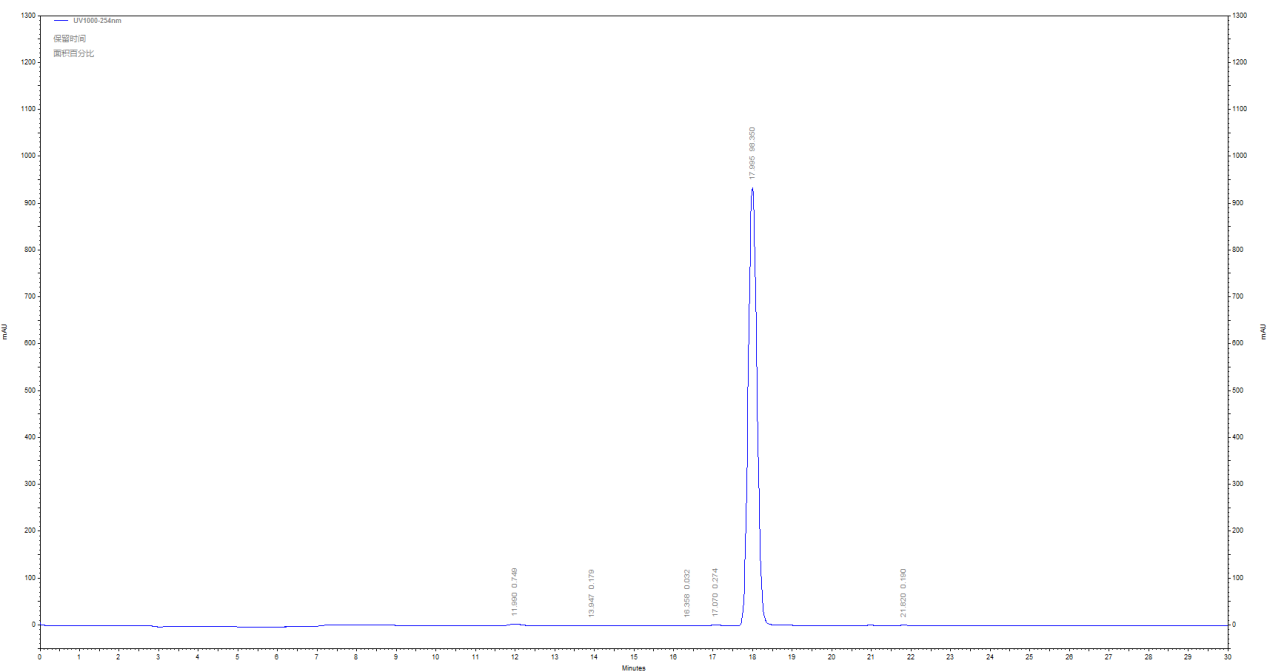


^1^H NMR spectrum of compound **13e**（400 MHz, DMSO-*d*_6_）


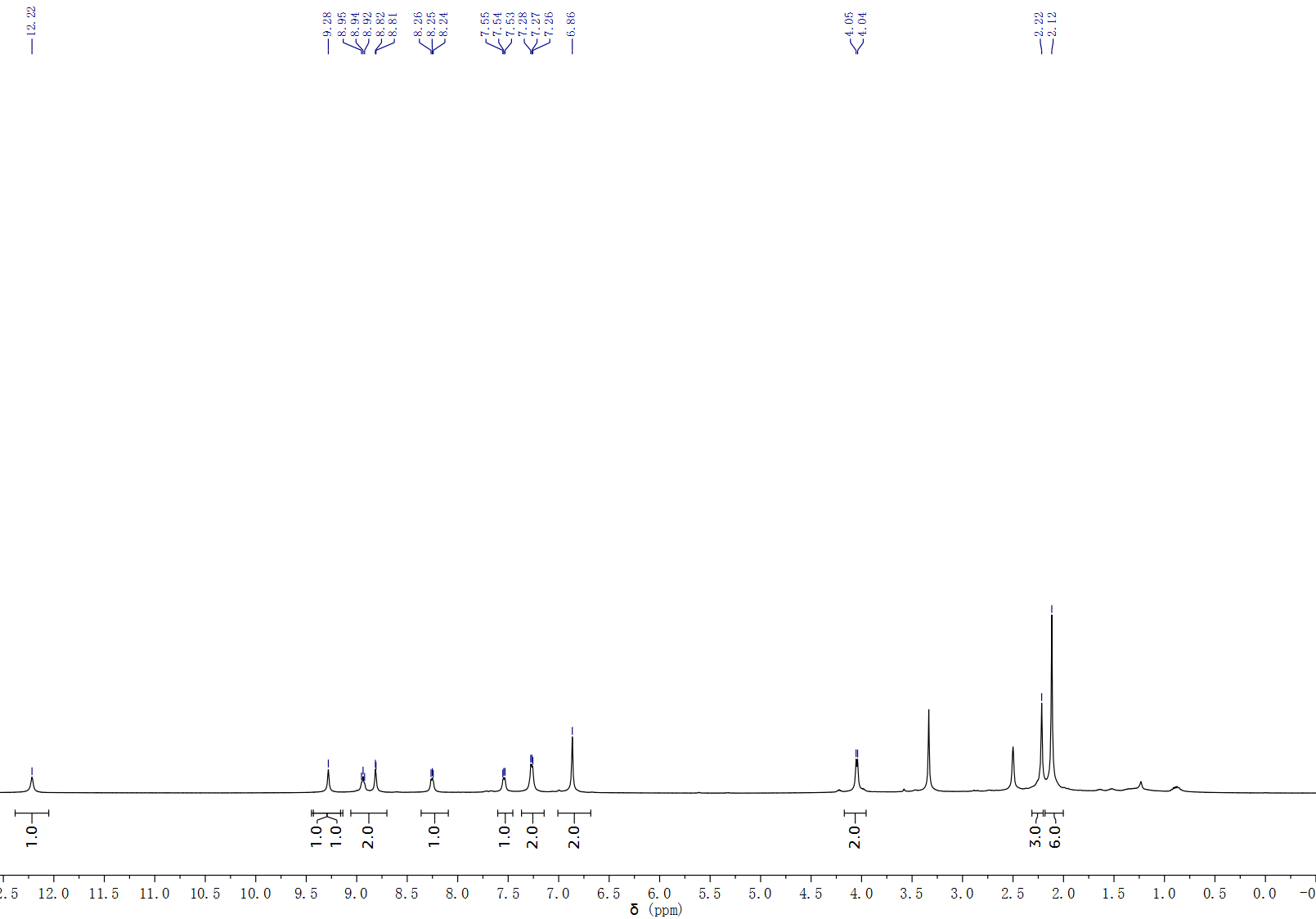


^13^C NMR spectrum of compound **13e**（100 MHz, DMSO-*d*_6_）


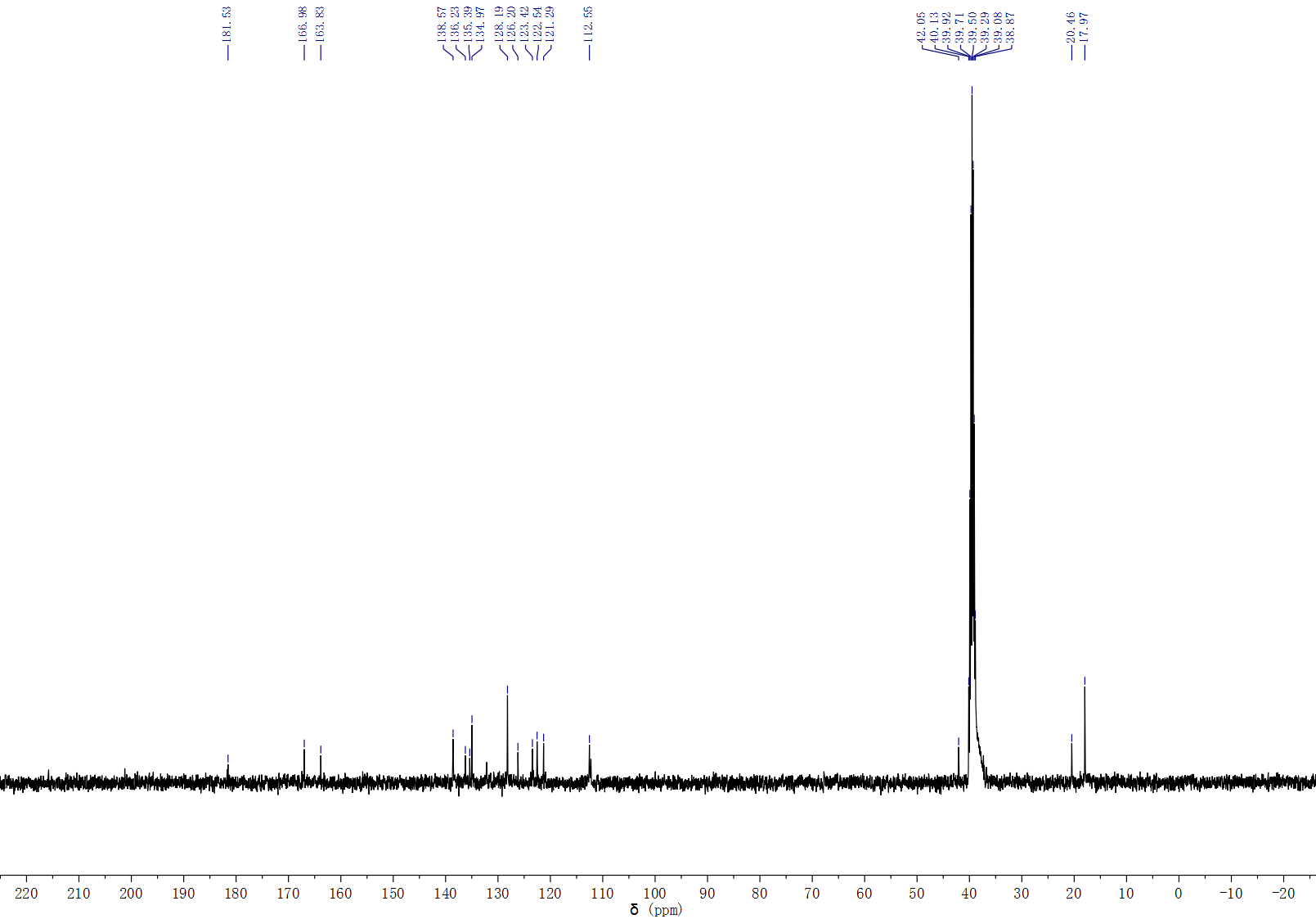


MS(ESI) spectrum of compound **13e**.

HPLC chromatogram of compound **13e**.


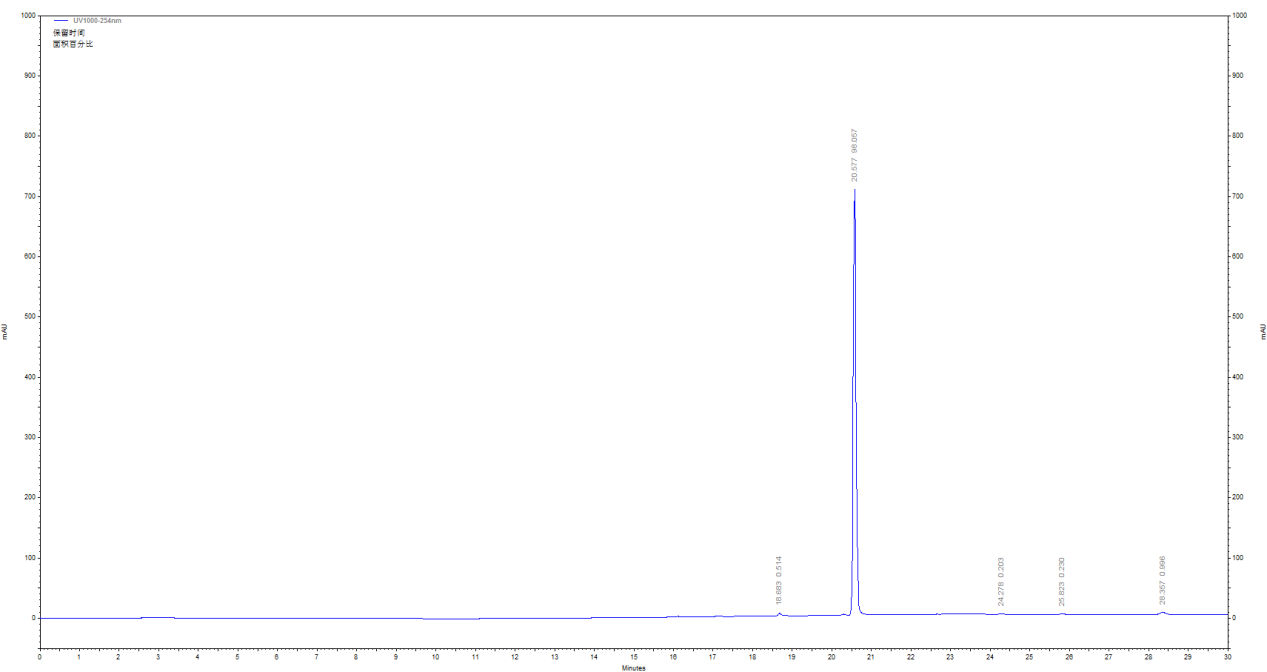


^1^H NMR spectrum of compound **13f**（400 MHz, DMSO-*d*_6_）


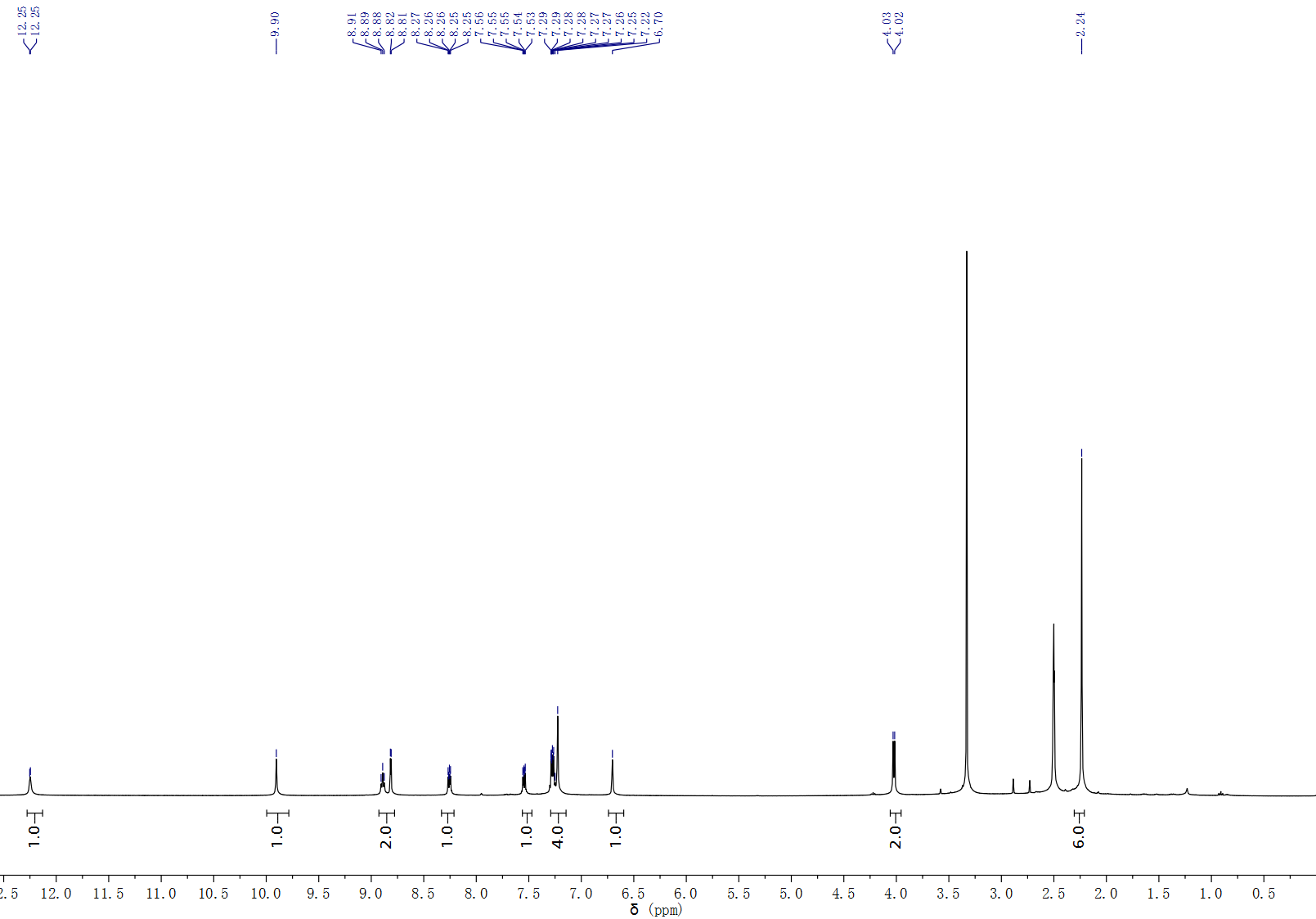


^13^C NMR spectrum of compound **13f**（100 MHz, DMSO-*d*_6_）


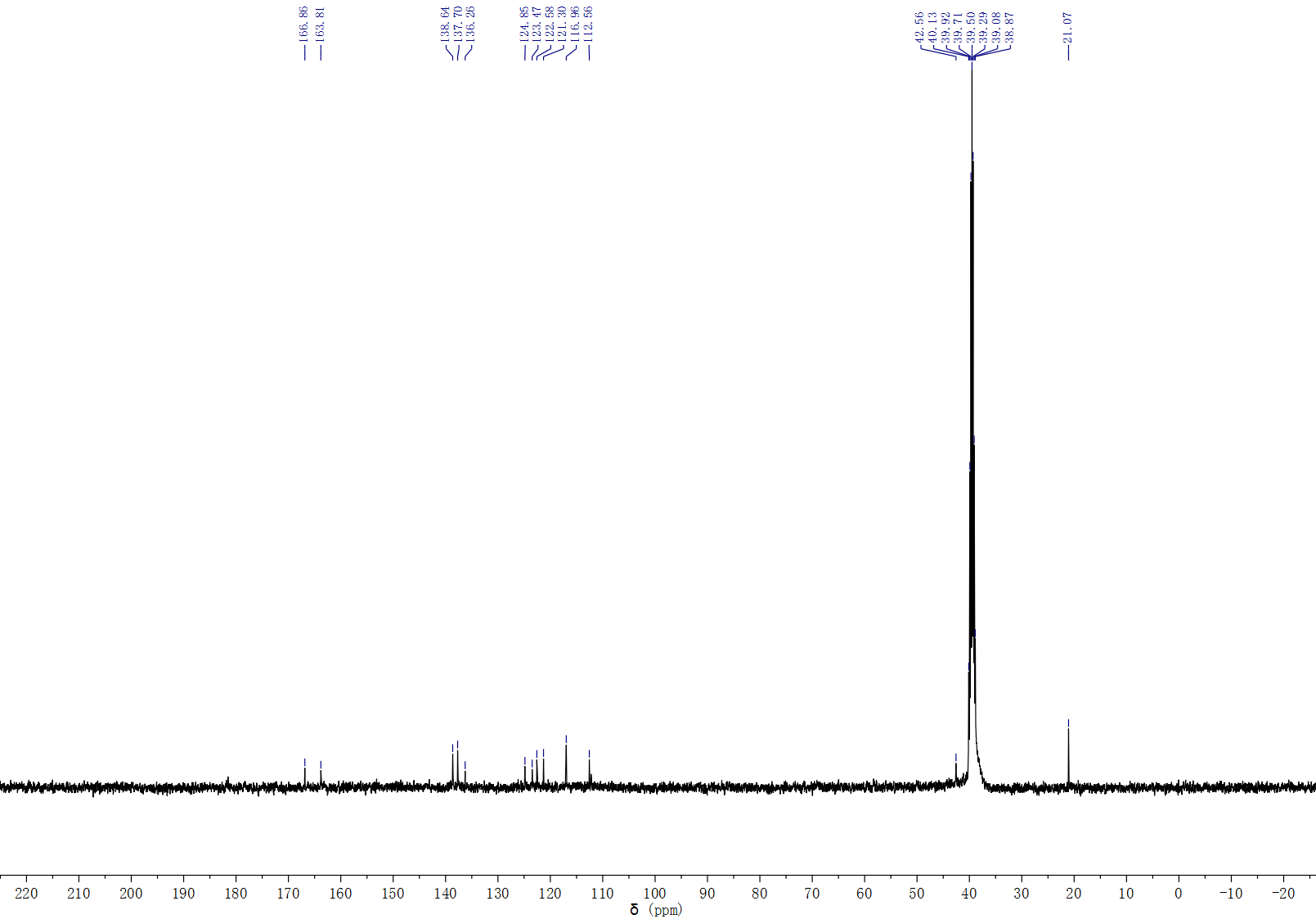


MS(ESI) spectrum of compound **13f**.

HPLC chromatogram of compound **13f**.


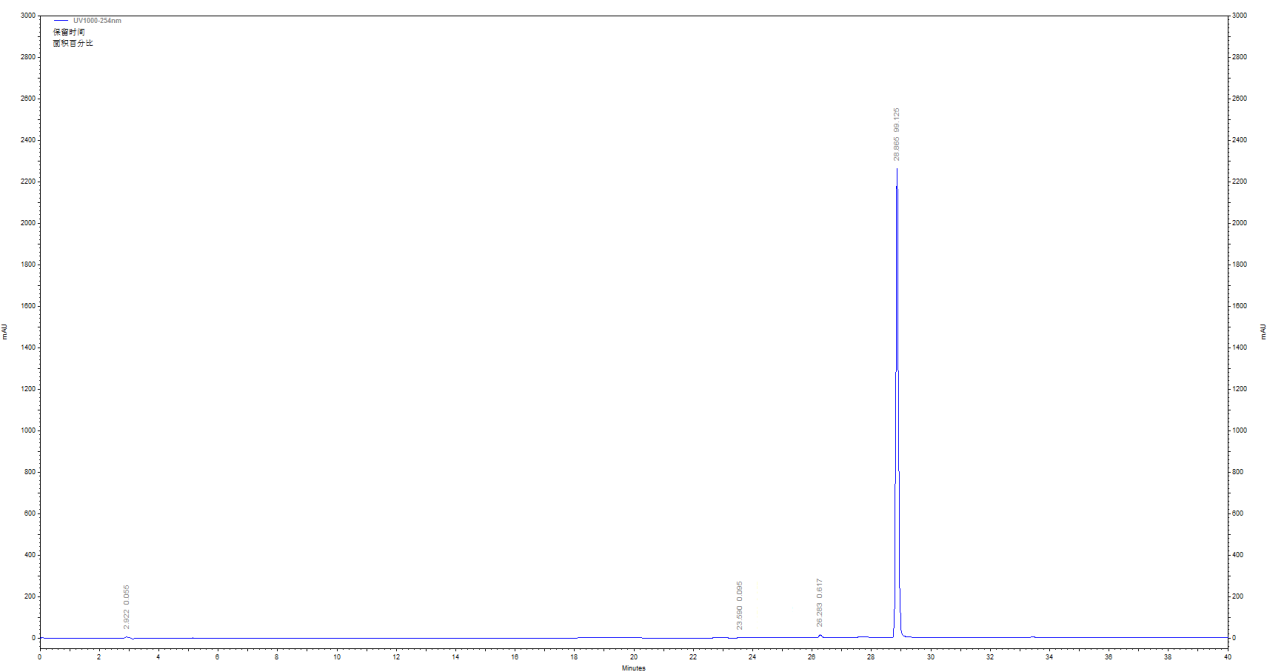


^1^H NMR spectrum of compound **13g**（400 MHz, DMSO-*d*_6_）


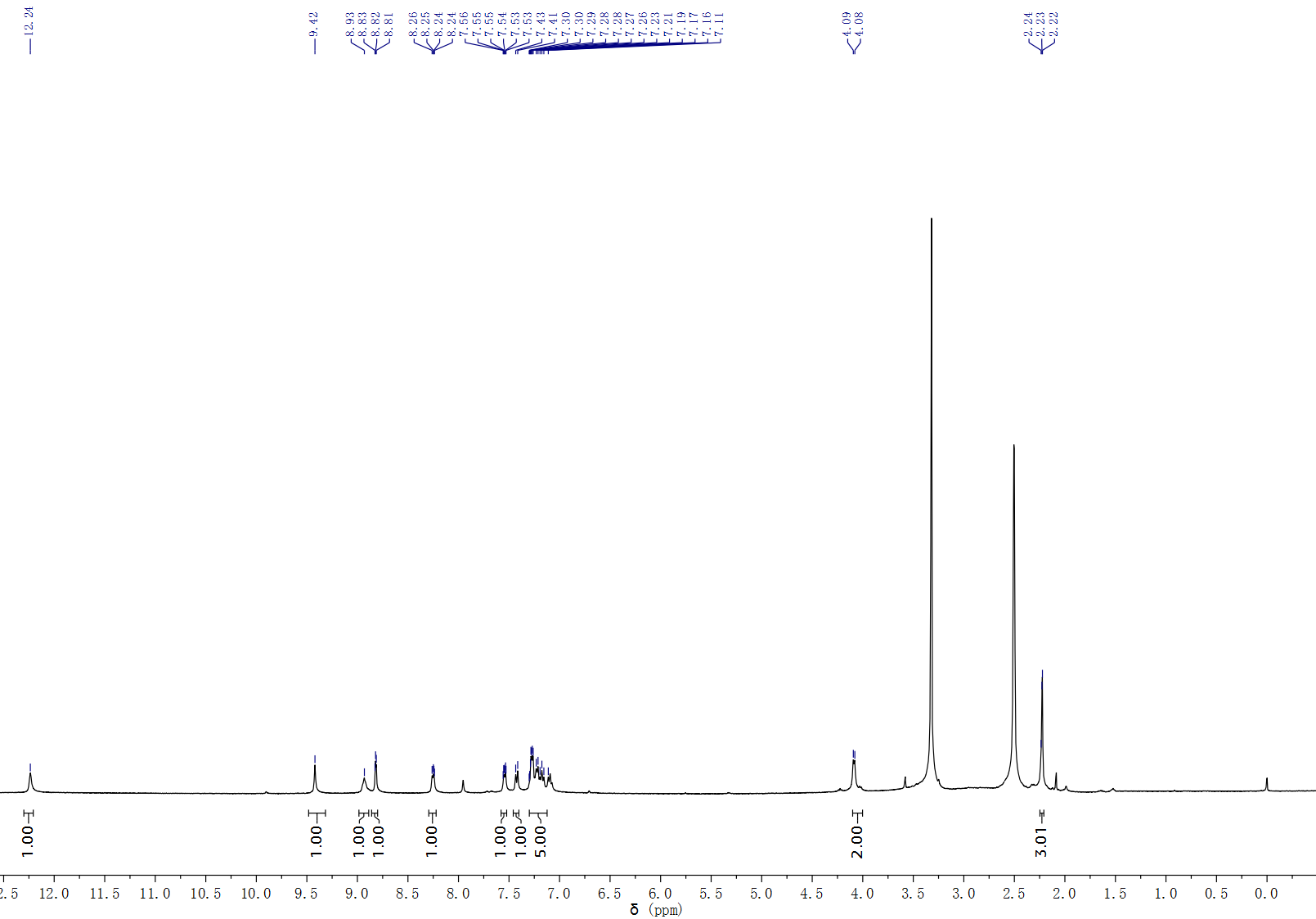


^13^C NMR spectrum of compound **13g**（100 MHz, DMSO-*d*_6_）


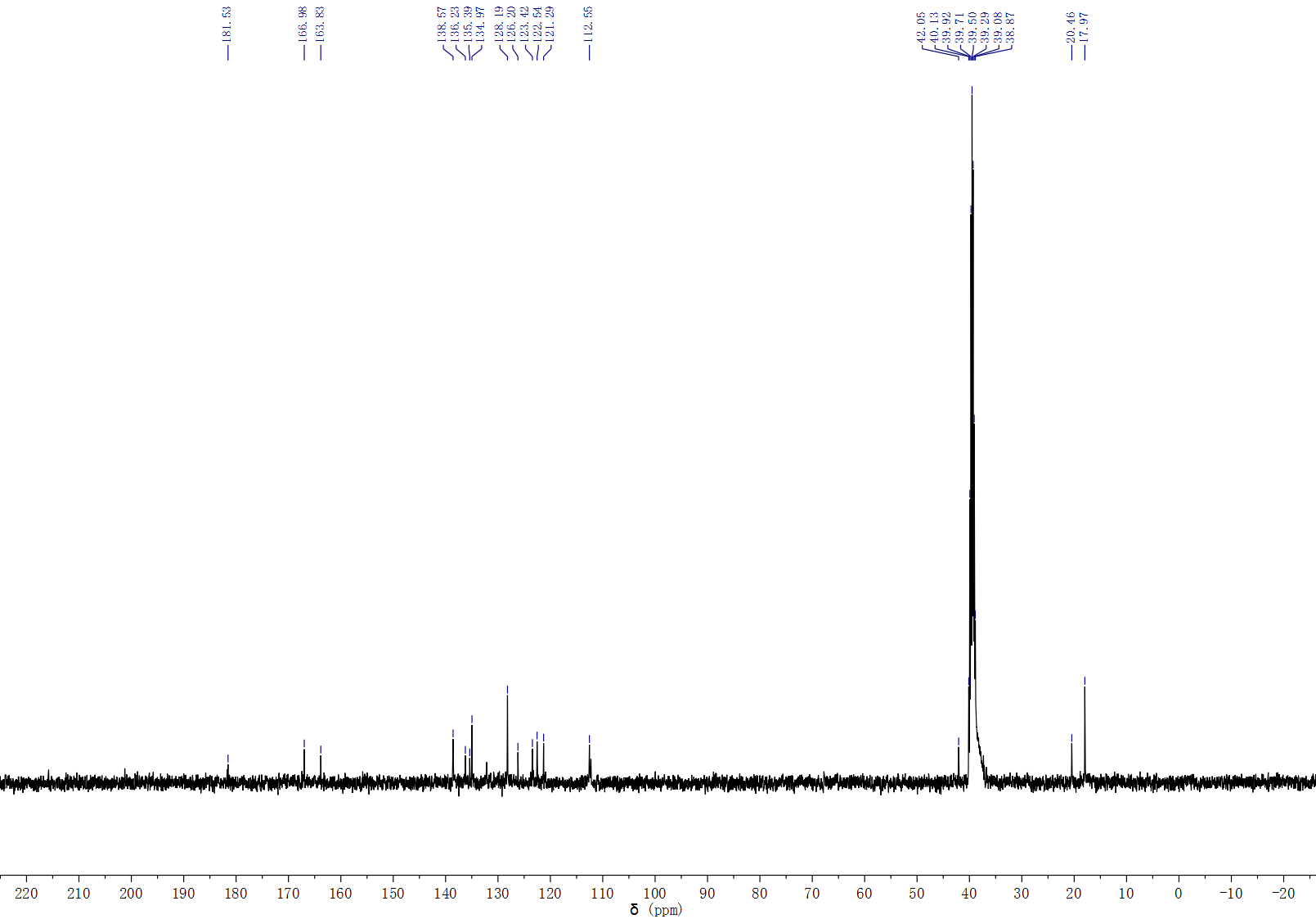


MS(ESI) spectrum of compound **13g**.

HPLC chromatogram of compound **13g**.


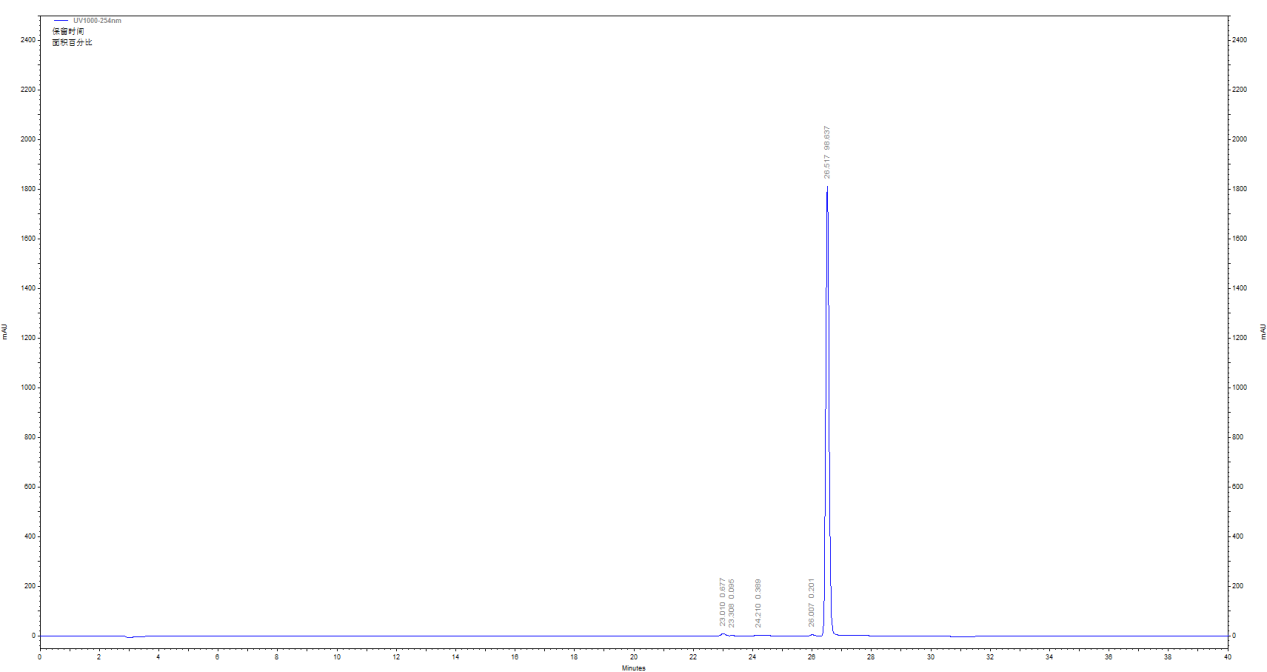


^1^H NMR spectrum of compound **13h**（400 MHz, DMSO-*d*_6_）


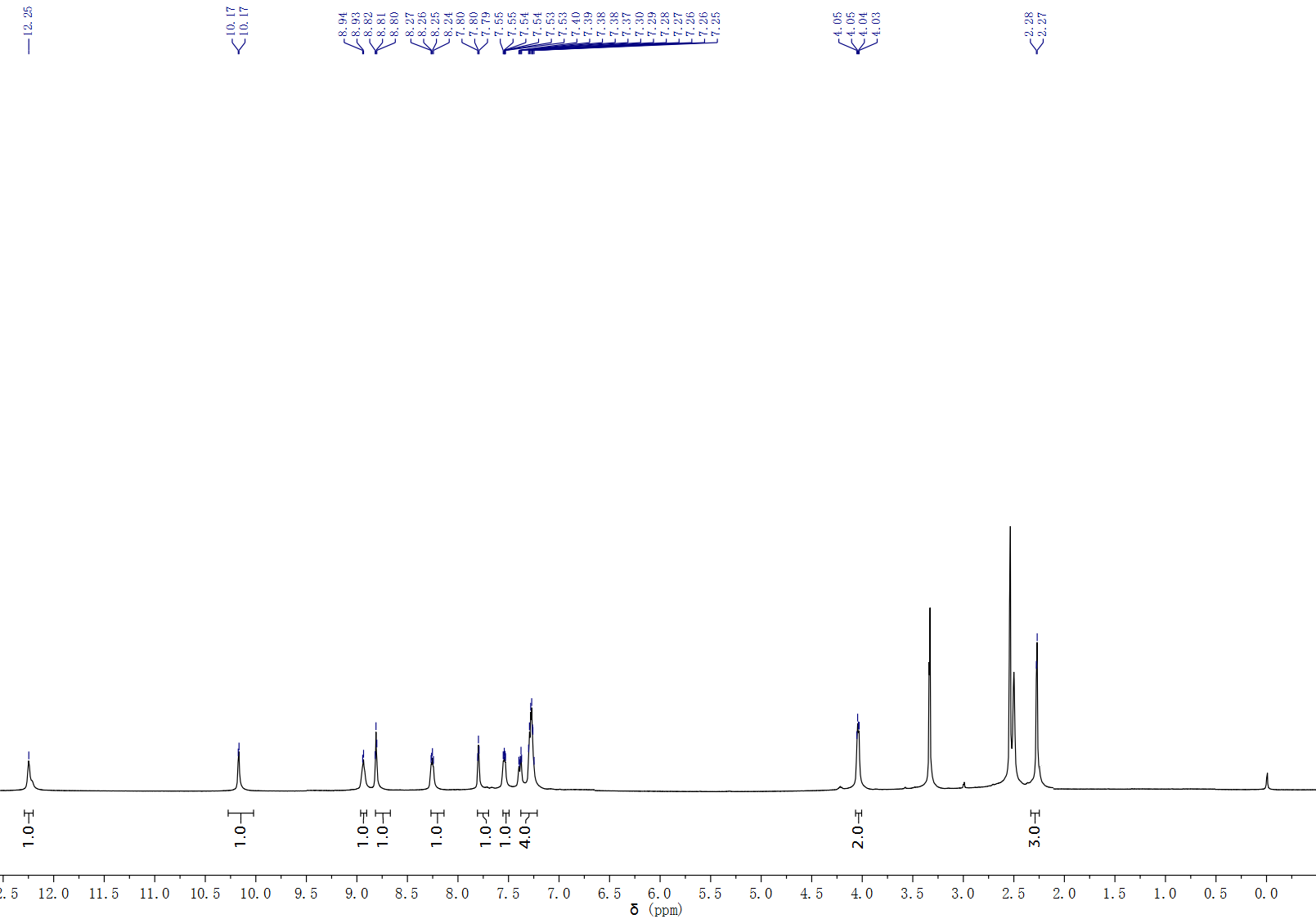


^13^C NMR spectrum of compound **13h**（100 MHz, DMSO-*d*_6_）


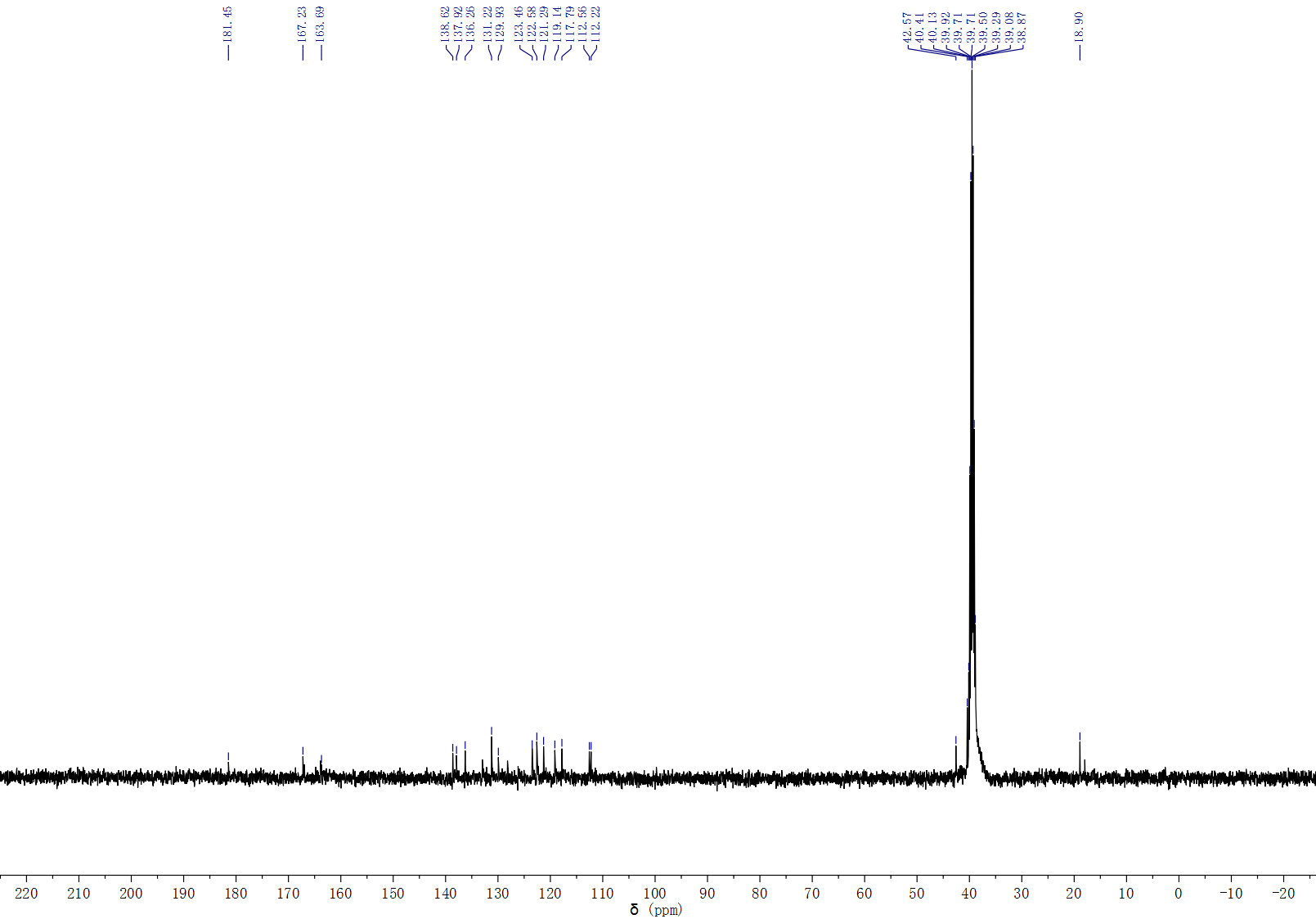


MS(ESI) spectrum of compound **13h**.

HPLC chromatogram of compound **13h**.


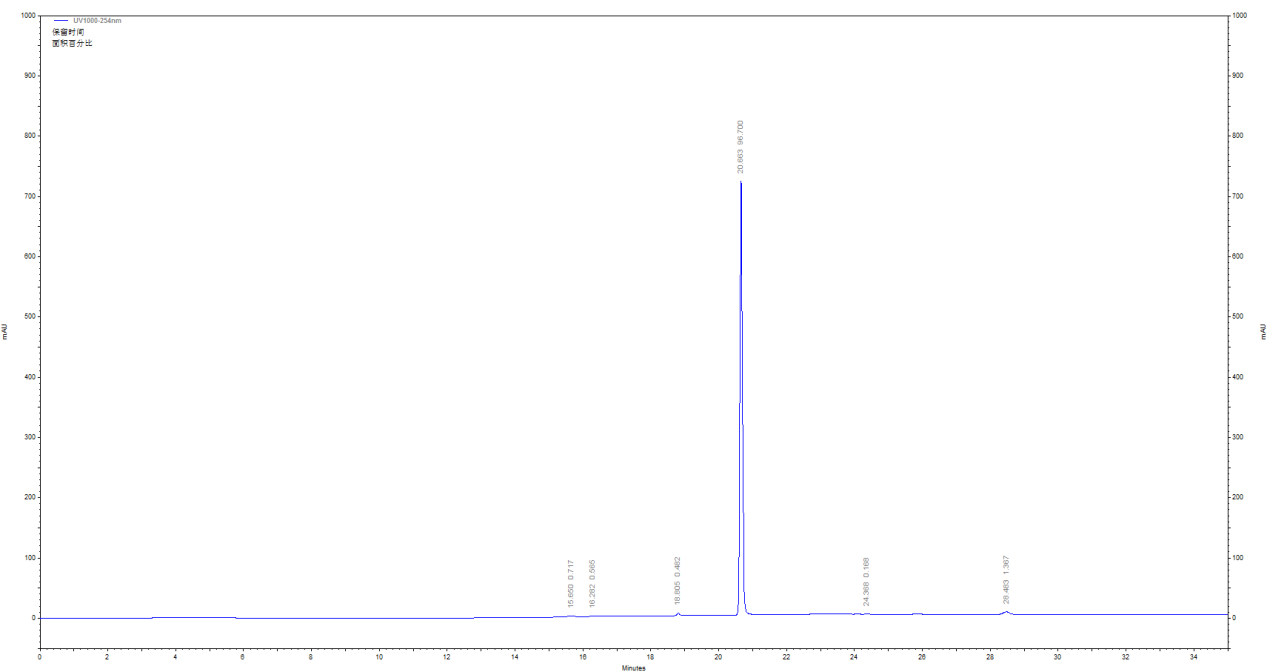


^1^H NMR spectrum of compound **13i**（400 MHz, DMSO-*d*_6_）


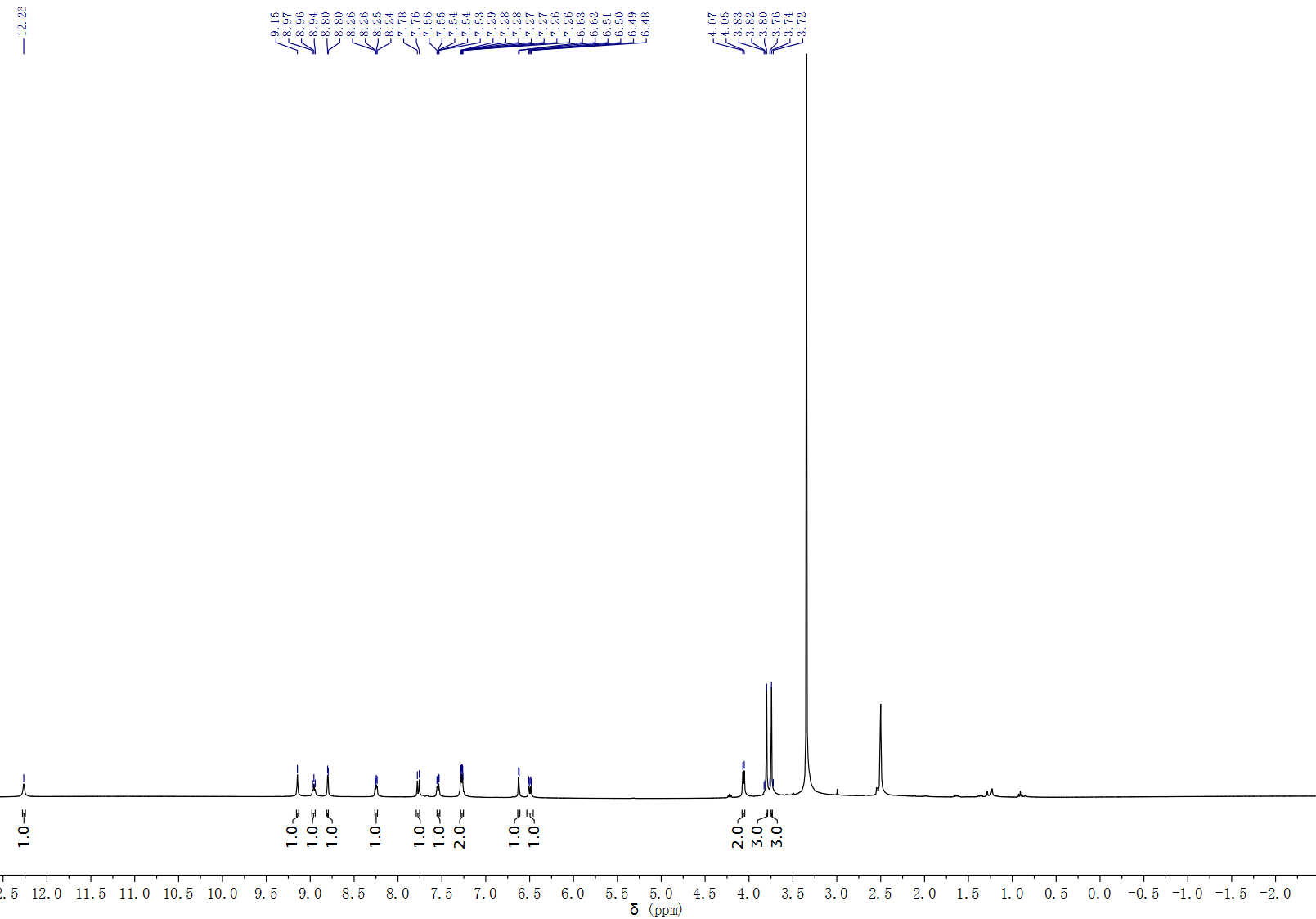


^13^C NMR spectrum of compound **13i**（100 MHz, DMSO-*d*_6_）


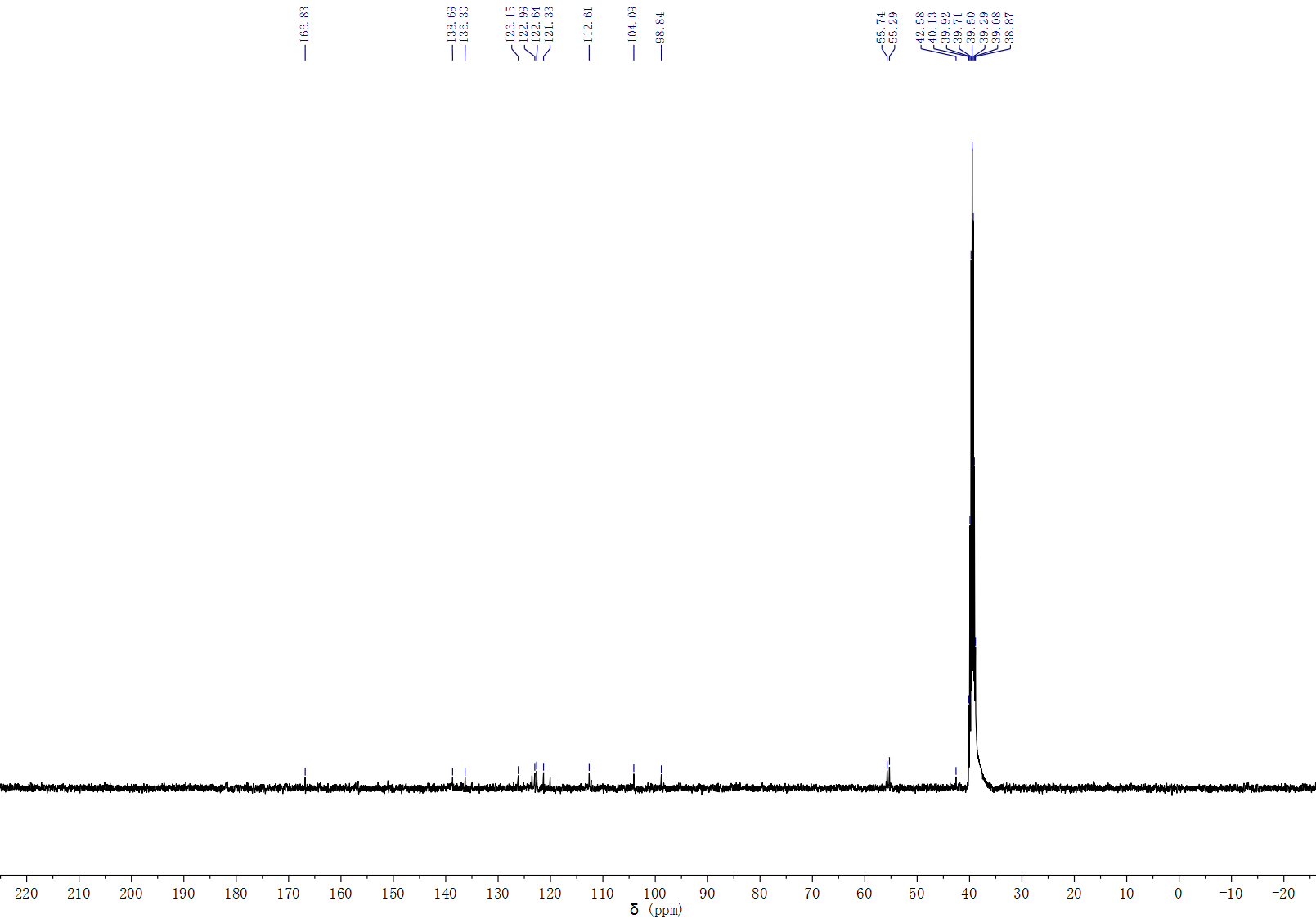


MS(ESI) spectrum of compound **13i**.

HPLC chromatogram of compound **13i**.


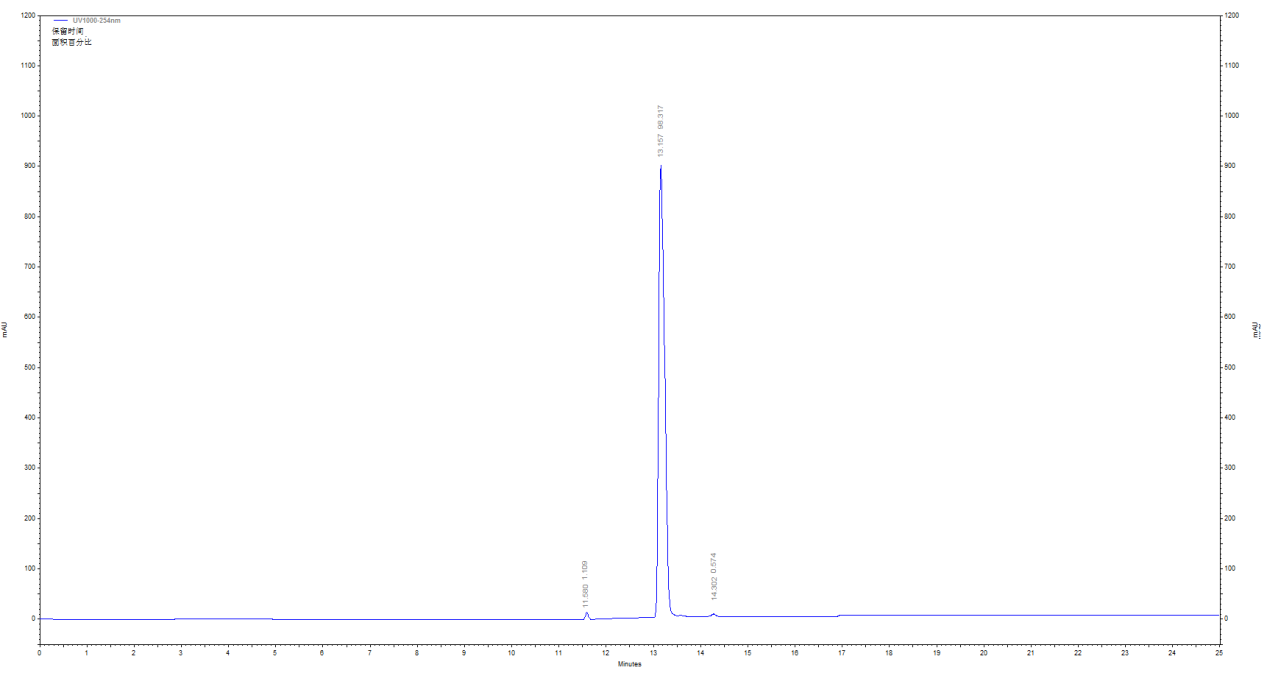


^1^H NMR spectrum of compound **13j**（400 MHz, DMSO-*d*_6_）


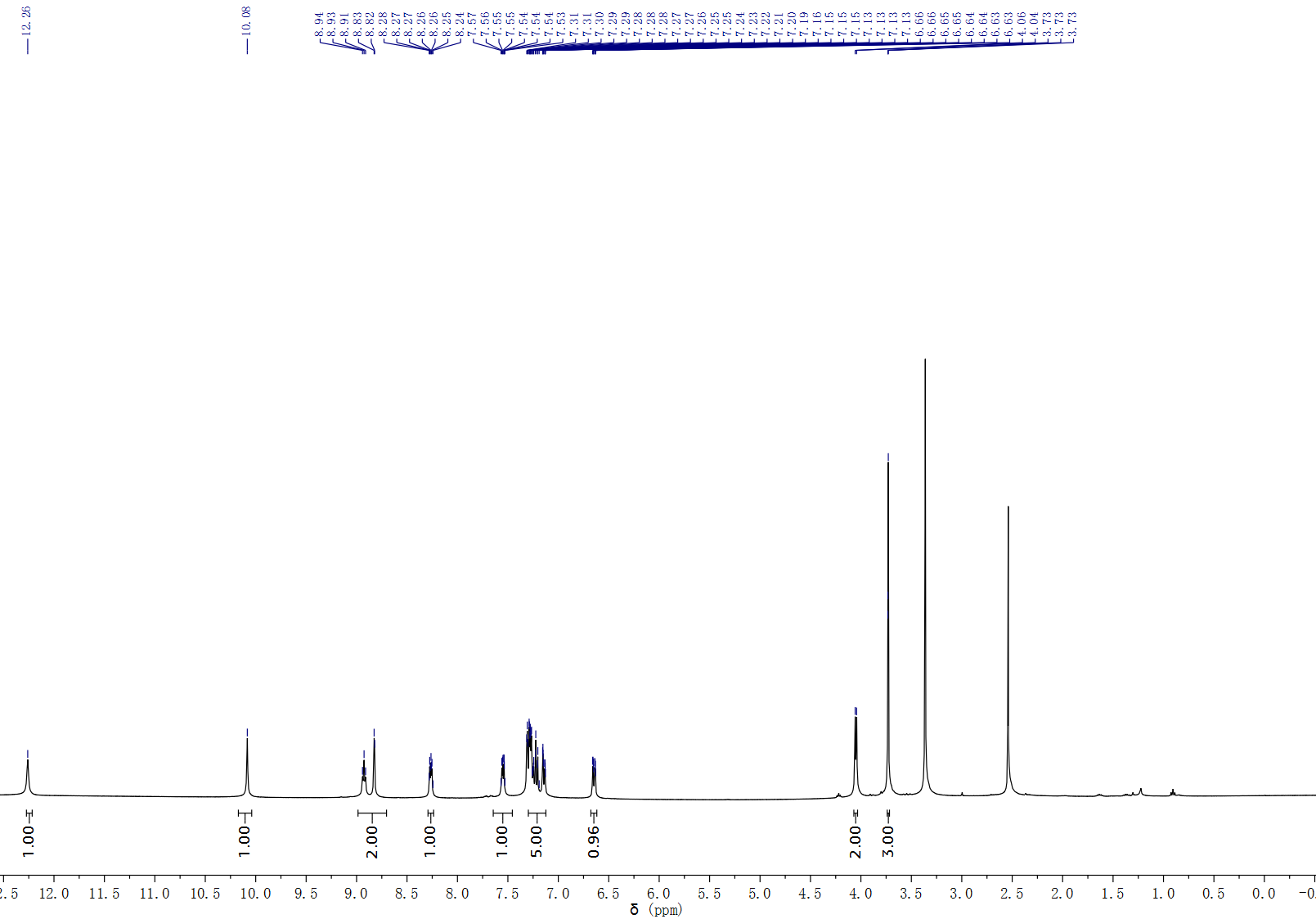


^13^C NMR spectrum of compound **13j**（100 MHz, DMSO-*d*_6_）


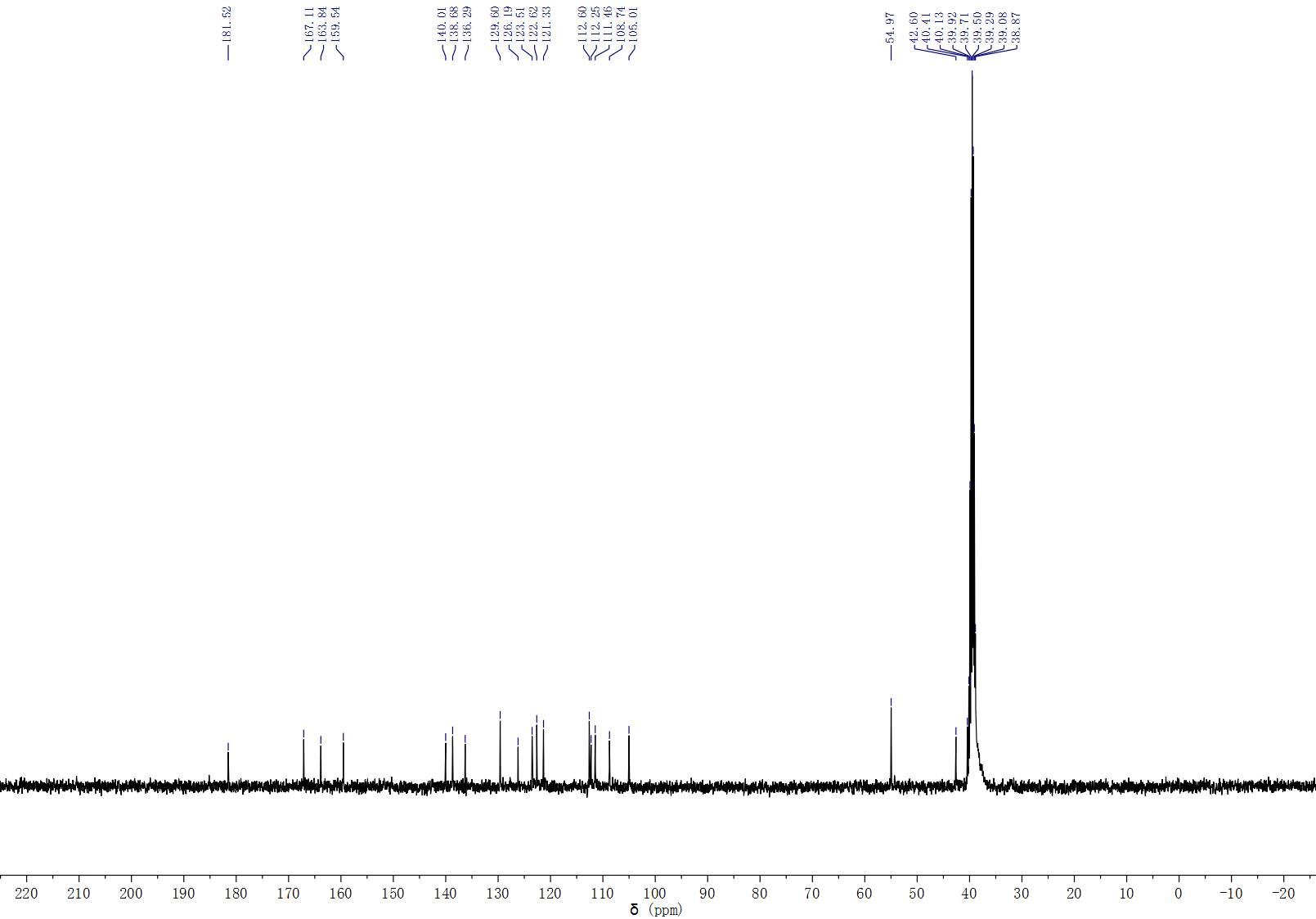


MS(ESI) spectrum of compound **13j**.

HPLC chromatogram of compound **13j**.


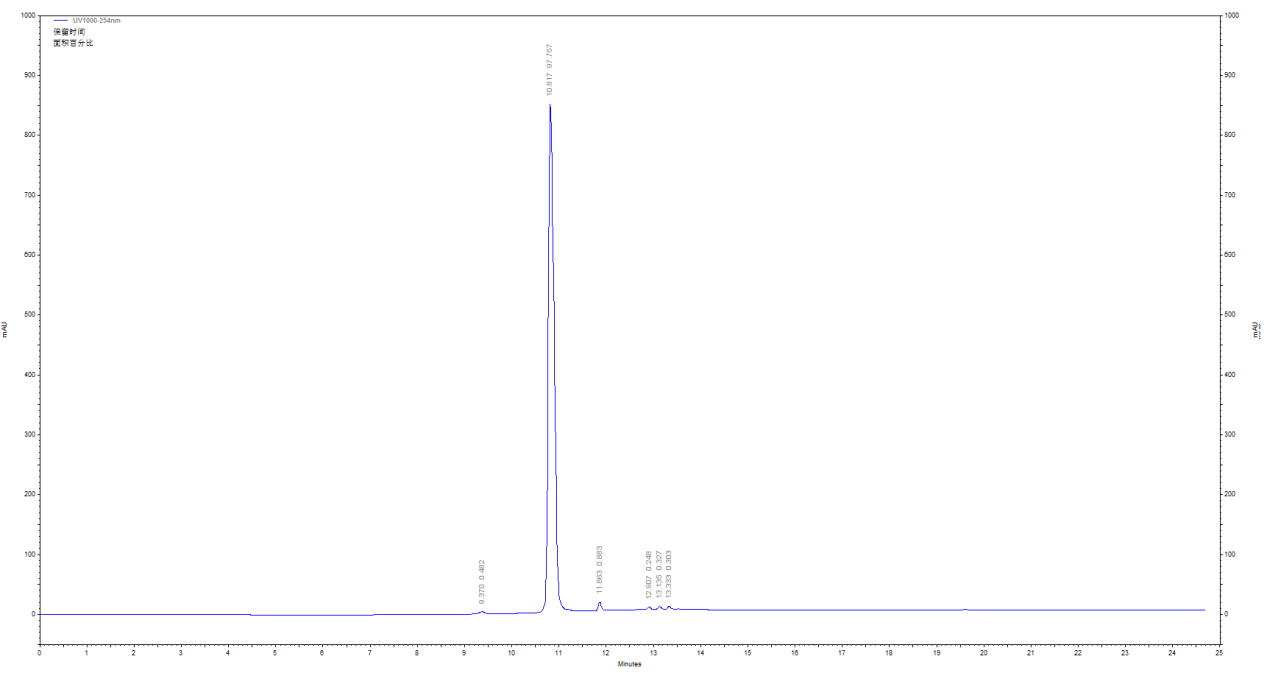


^1^H NMR spectrum of compound **13k**（400 MHz, DMSO-*d*_6_）


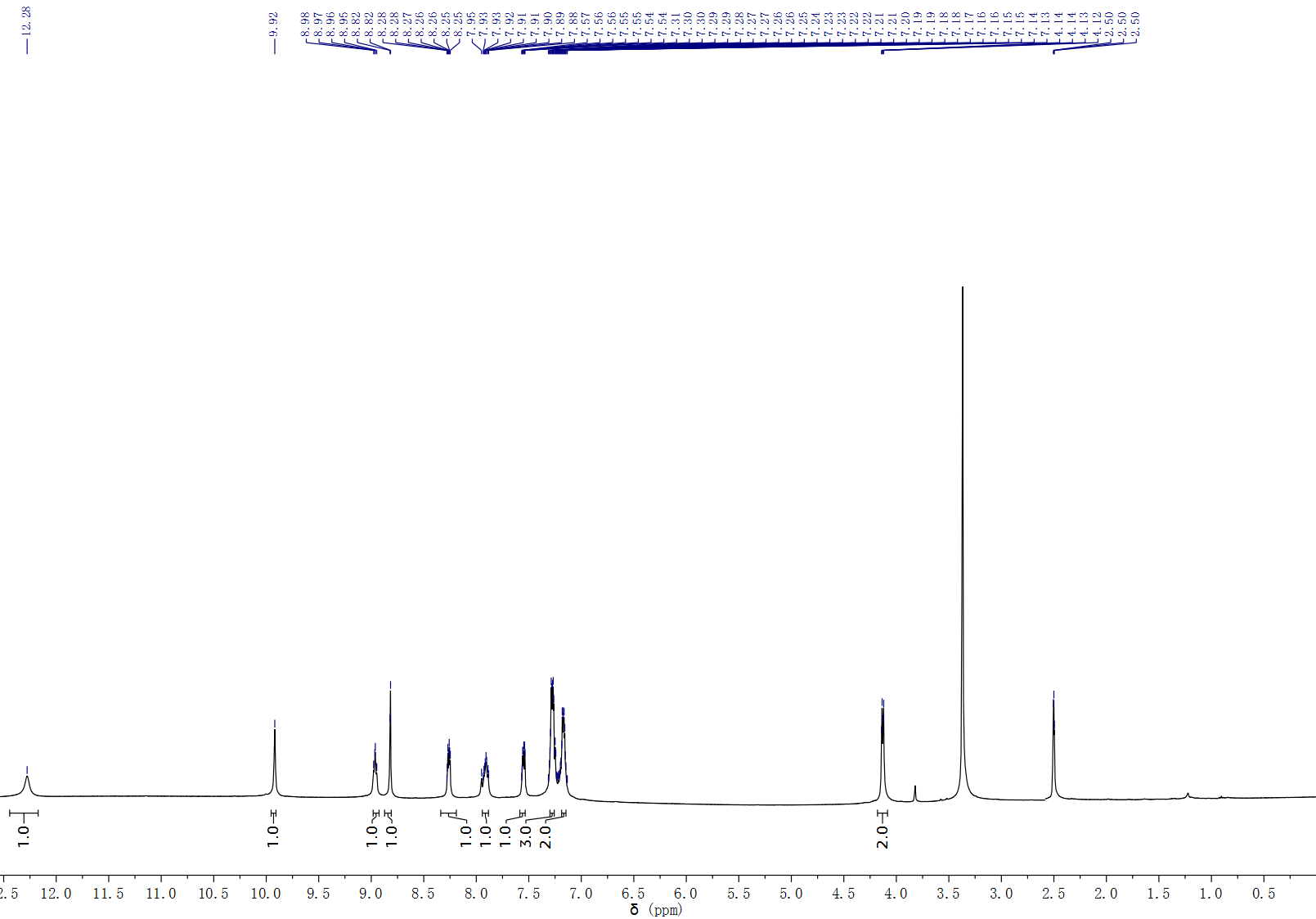


^13^C NMR spectrum of compound **13k**（100 MHz, DMSO-*d*_6_）


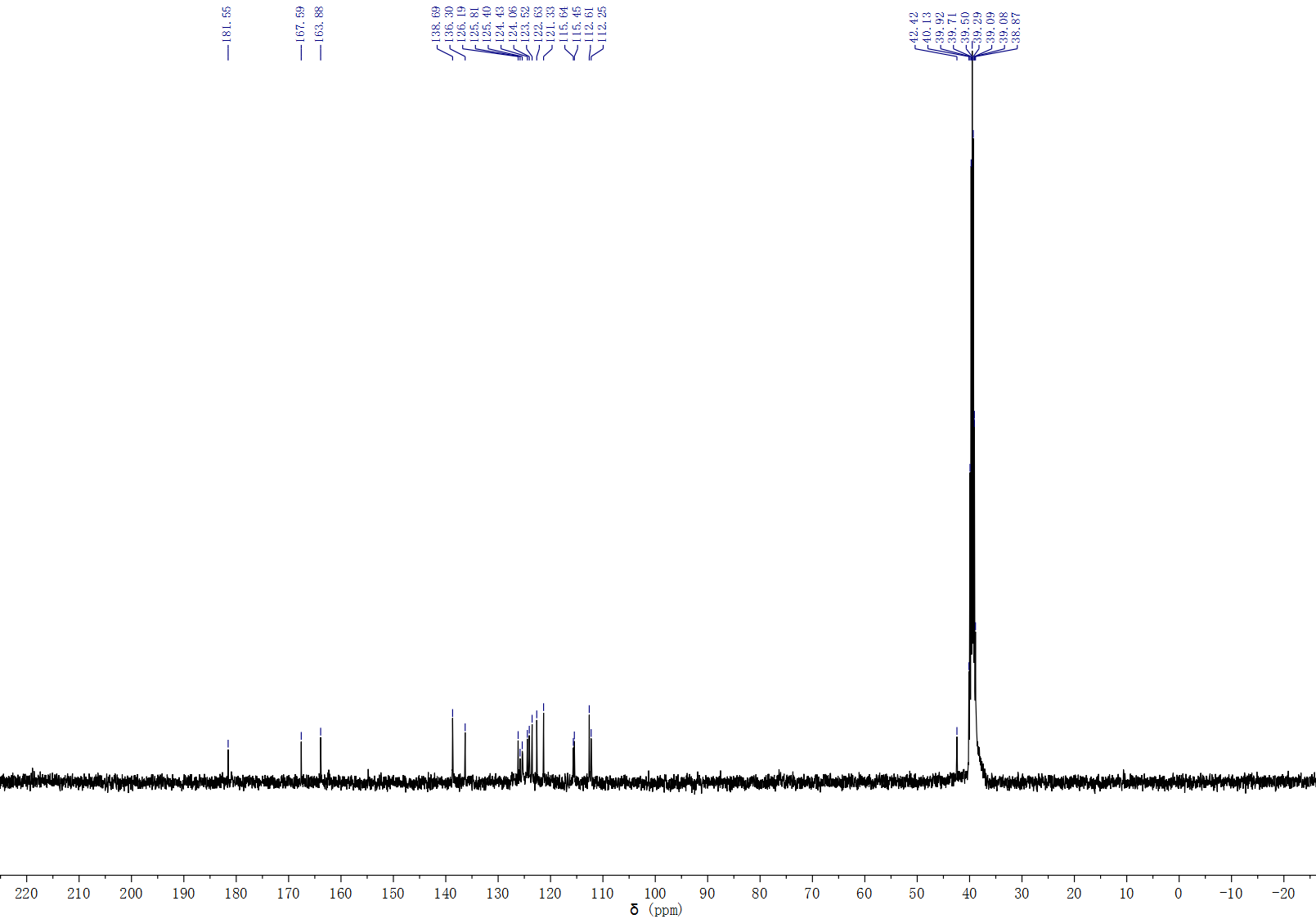


MS(ESI) spectrum of compound **13k**.

HPLC chromatogram of compound **13k**.


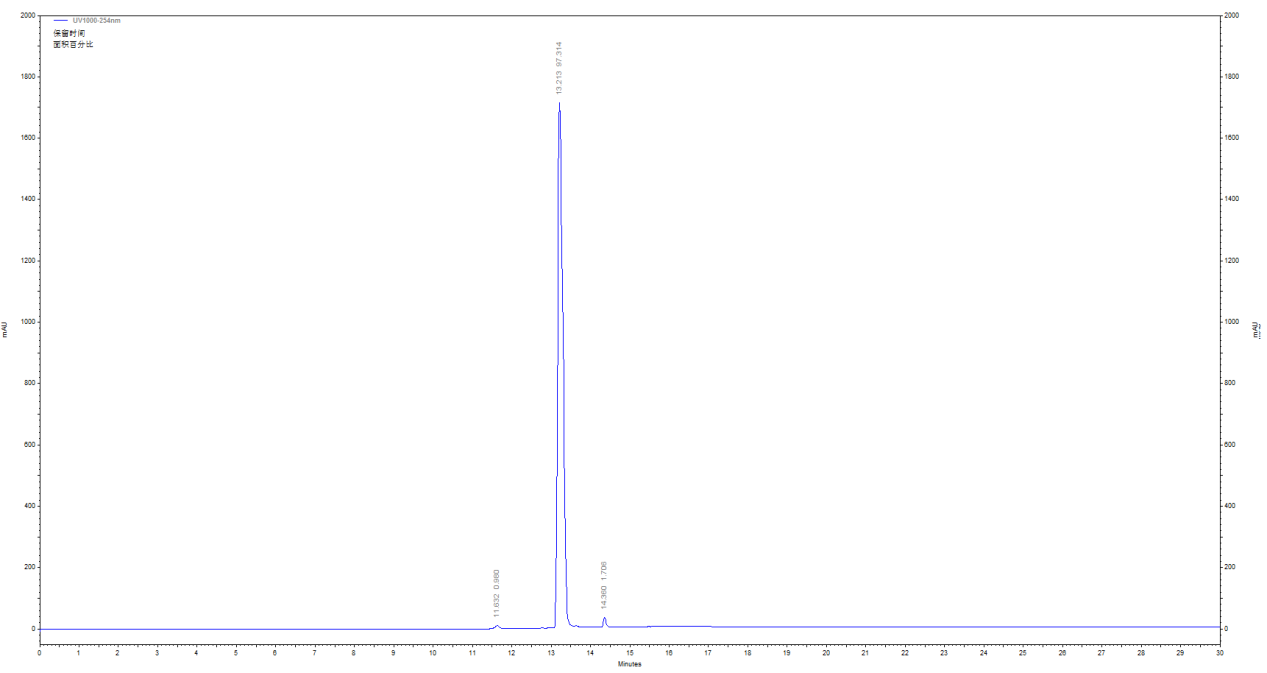


^1^H NMR spectrum of compound **17a**（400 MHz, DMSO-*d*_6_）


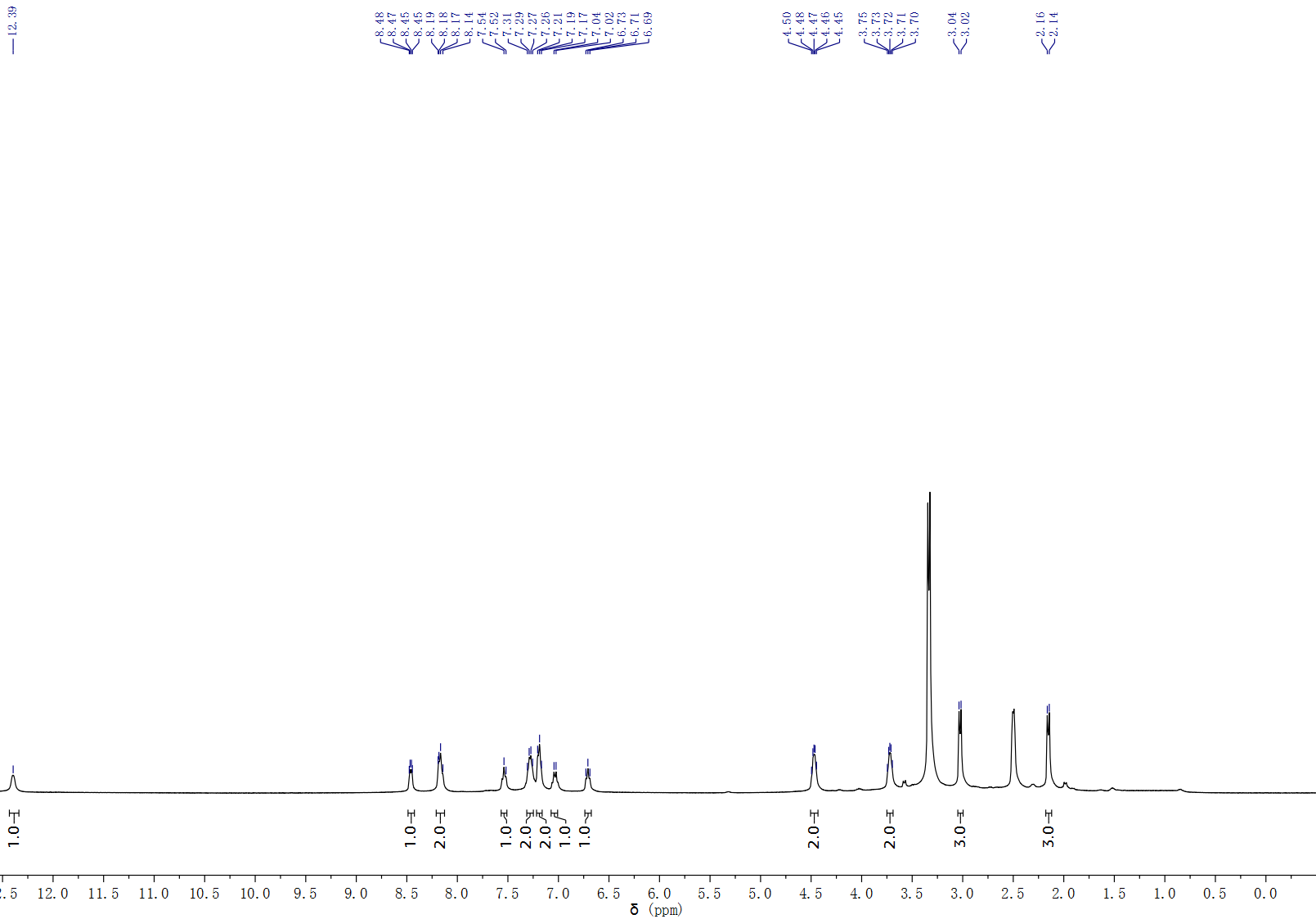


^13^C NMR spectrum of compound **17a**（100 MHz, DMSO-*d*_6_）


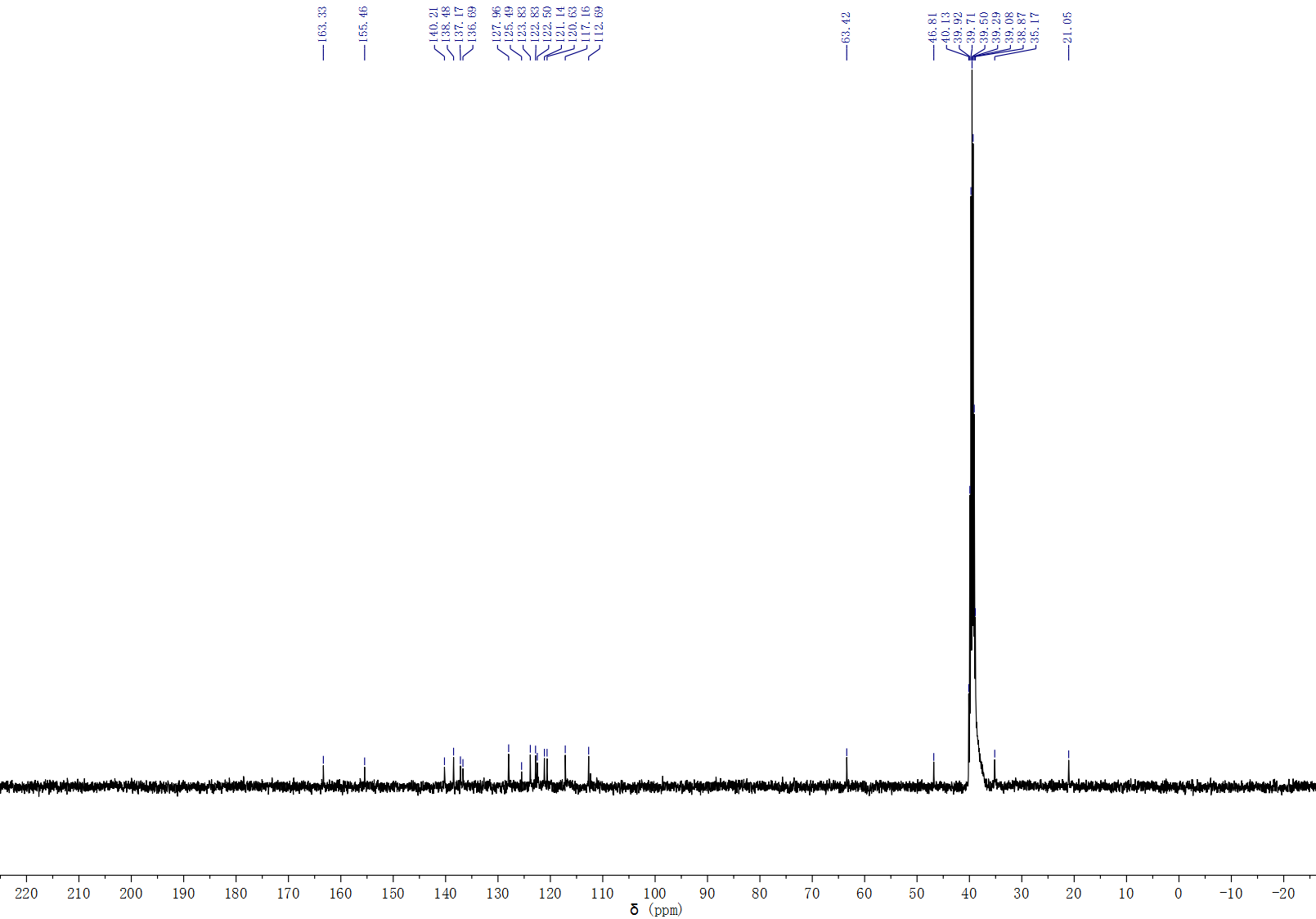


MS(ESI) spectrum of compound **17a**.

HPLC chromatogram of compound **17a**.


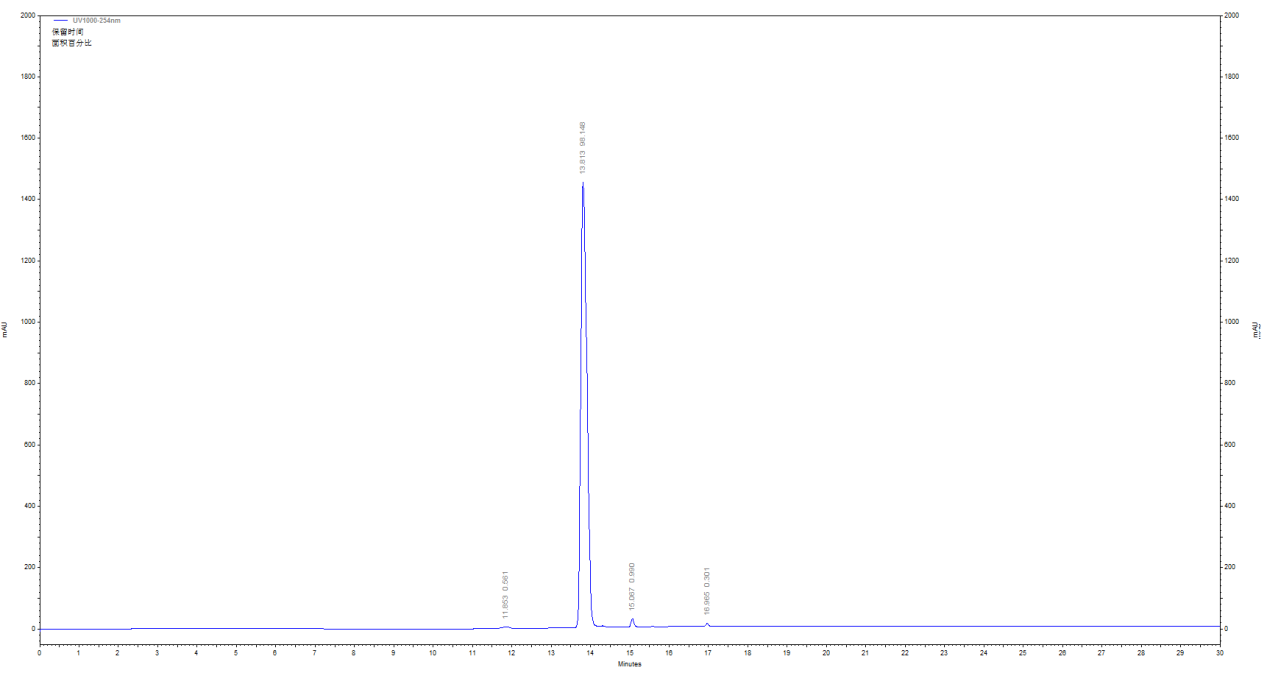


^1^H NMR spectrum of compound **17b**（400 MHz, DMSO-*d*_6_）


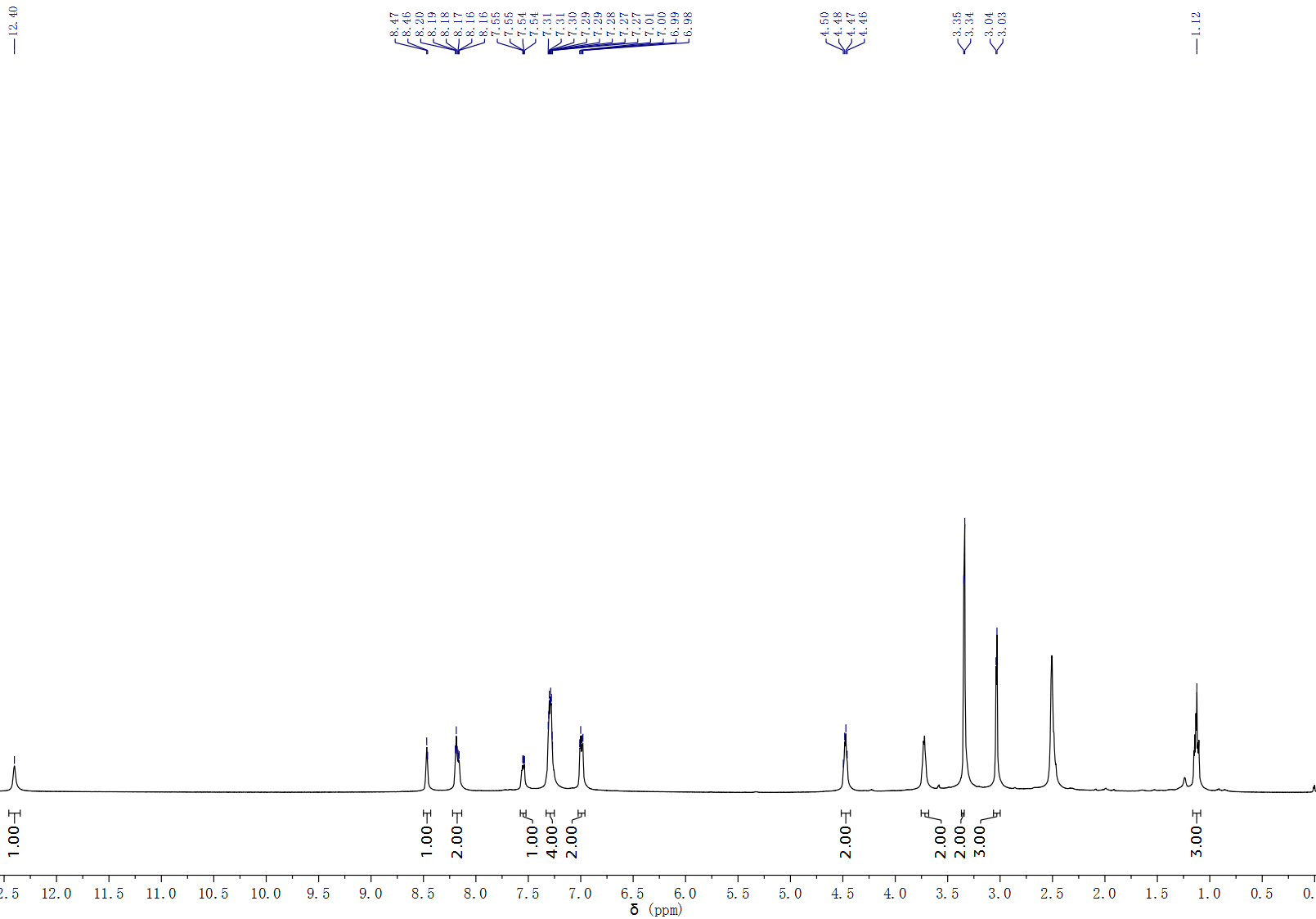


^13^C NMR spectrum of compound **17b**（100 MHz, DMSO-*d*_6_）


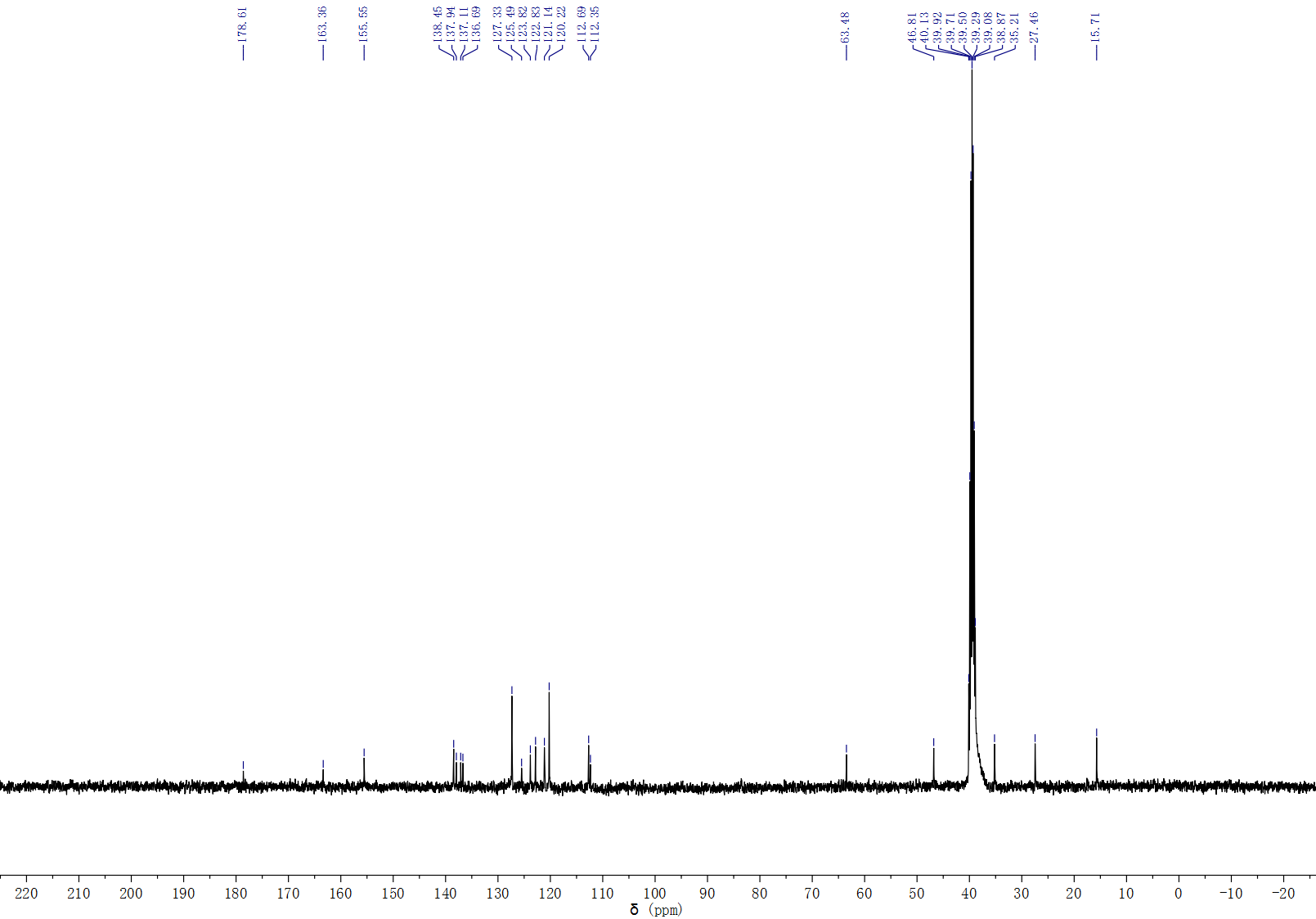


MS(ESI) spectrum of compound **17b**.

HPLC chromatogram of compound **17b**.


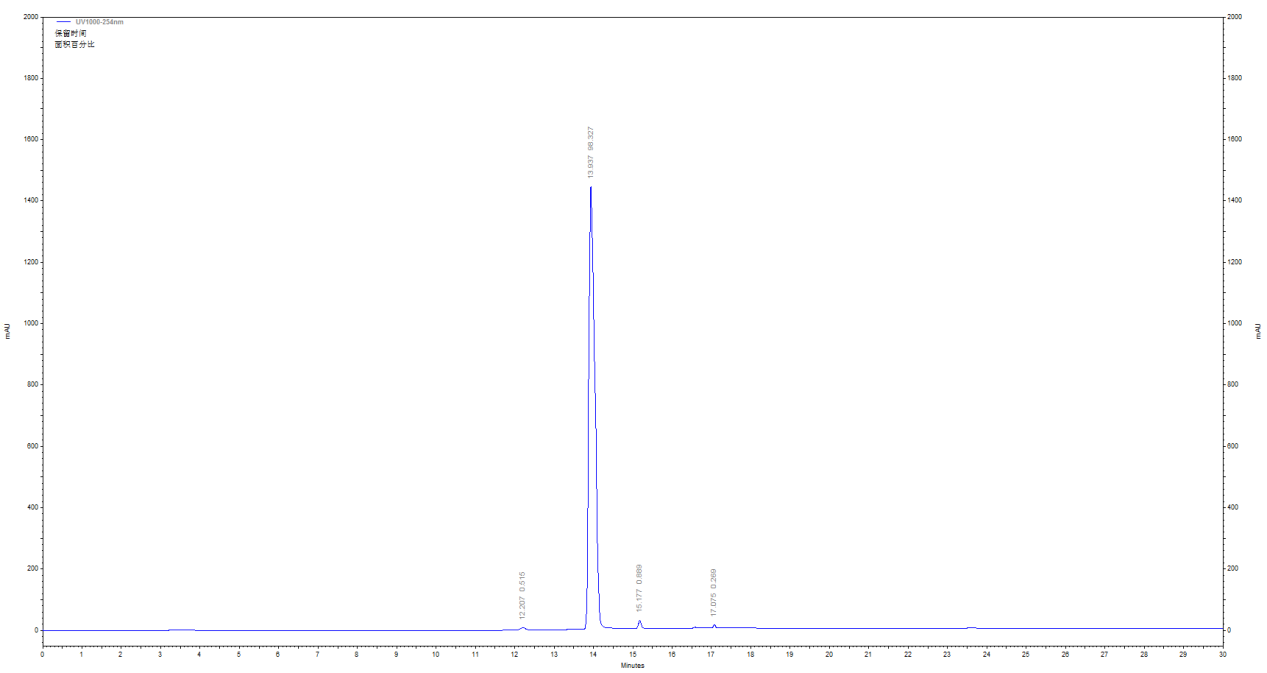


^1^H NMR spectrum of compound **17c**（400 MHz, DMSO-*d*_6_）


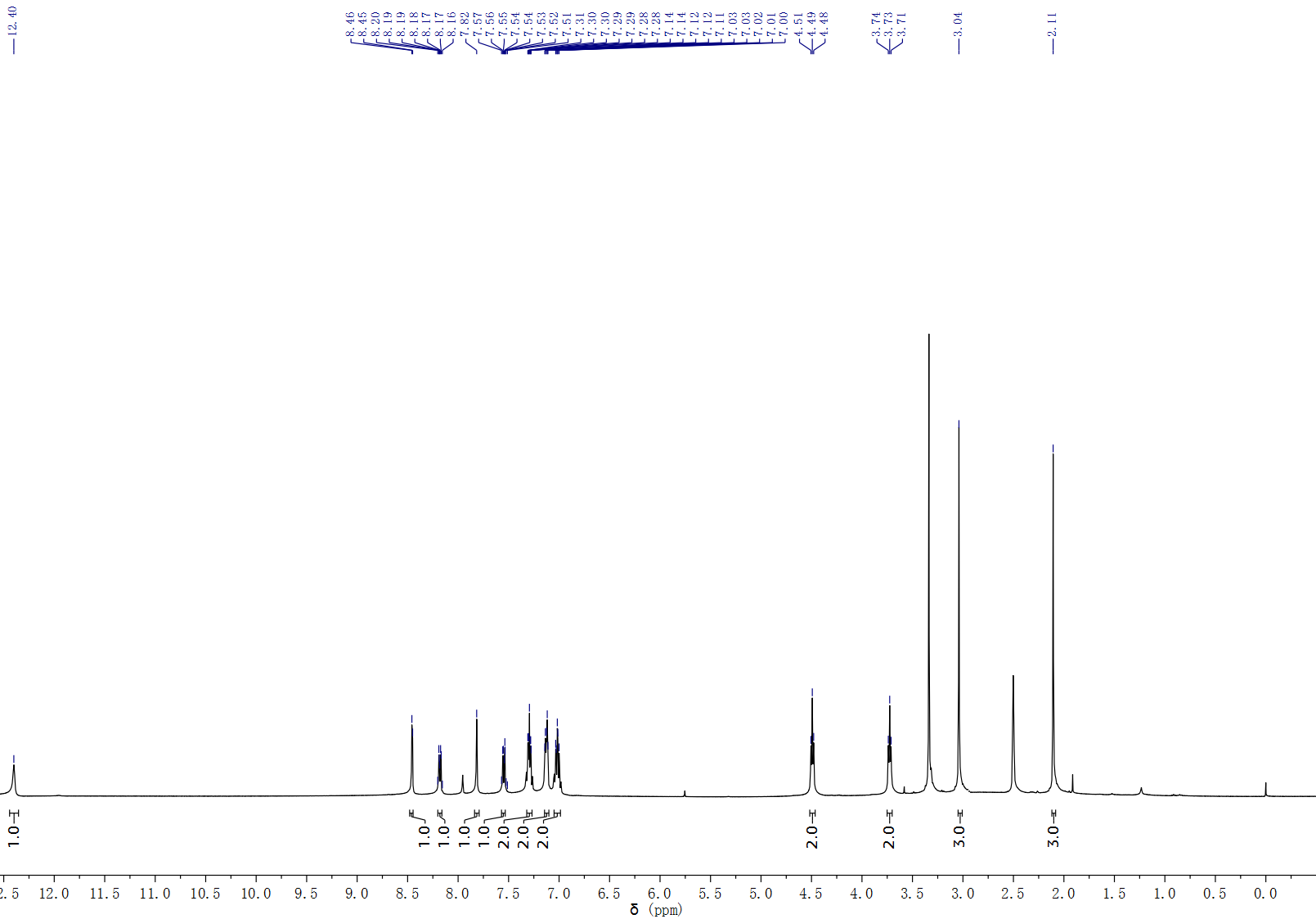


^13^C NMR spectrum of compound **17c**（100 MHz, DMSO-*d*_6_）


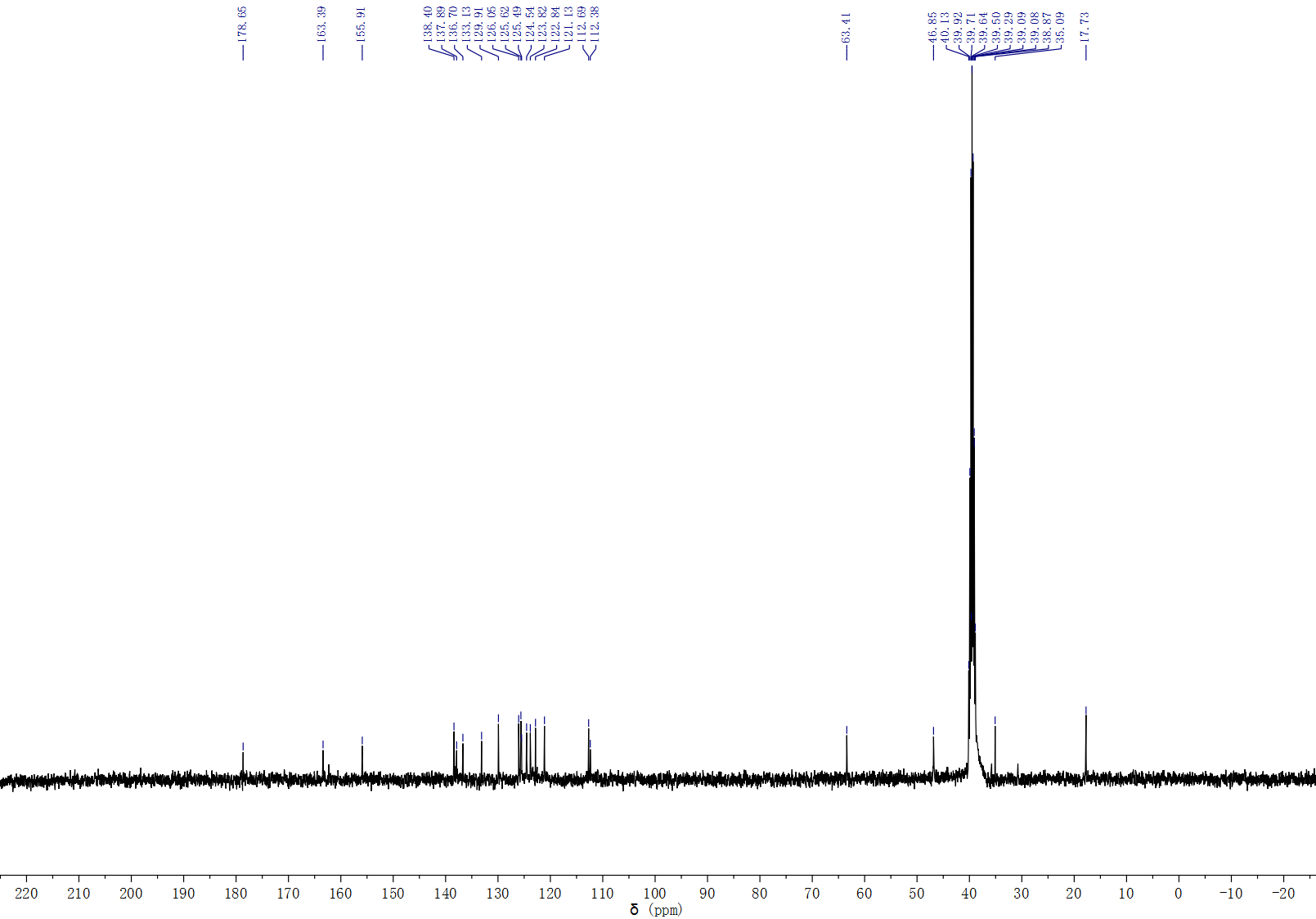


MS(ESI) spectrum of compound **17c**.

HPLC chromatogram of compound **17c**.


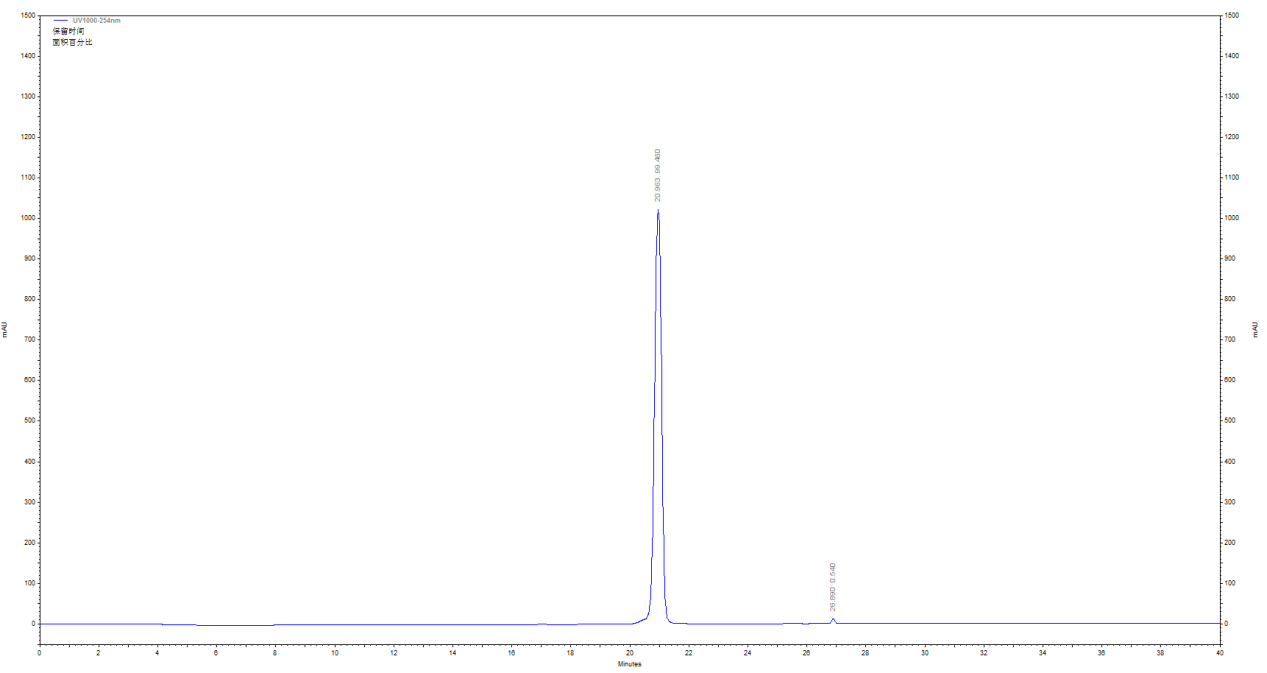


^1^H NMR spectrum of compound **17d**（400 MHz, DMSO-*d*_6_）


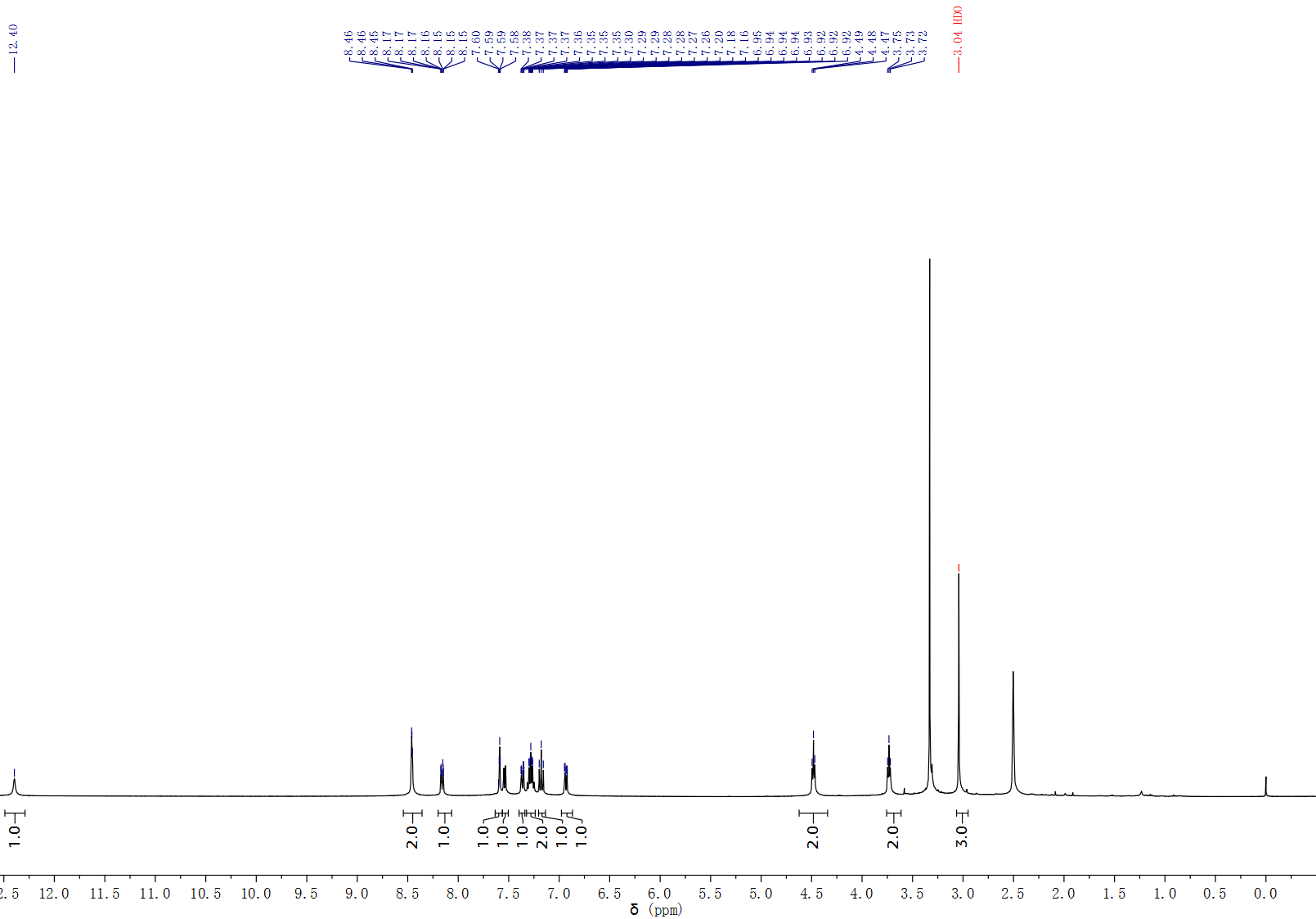


^13^C NMR spectrum of compound **17d**（100 MHz, DMSO-*d*_6_）


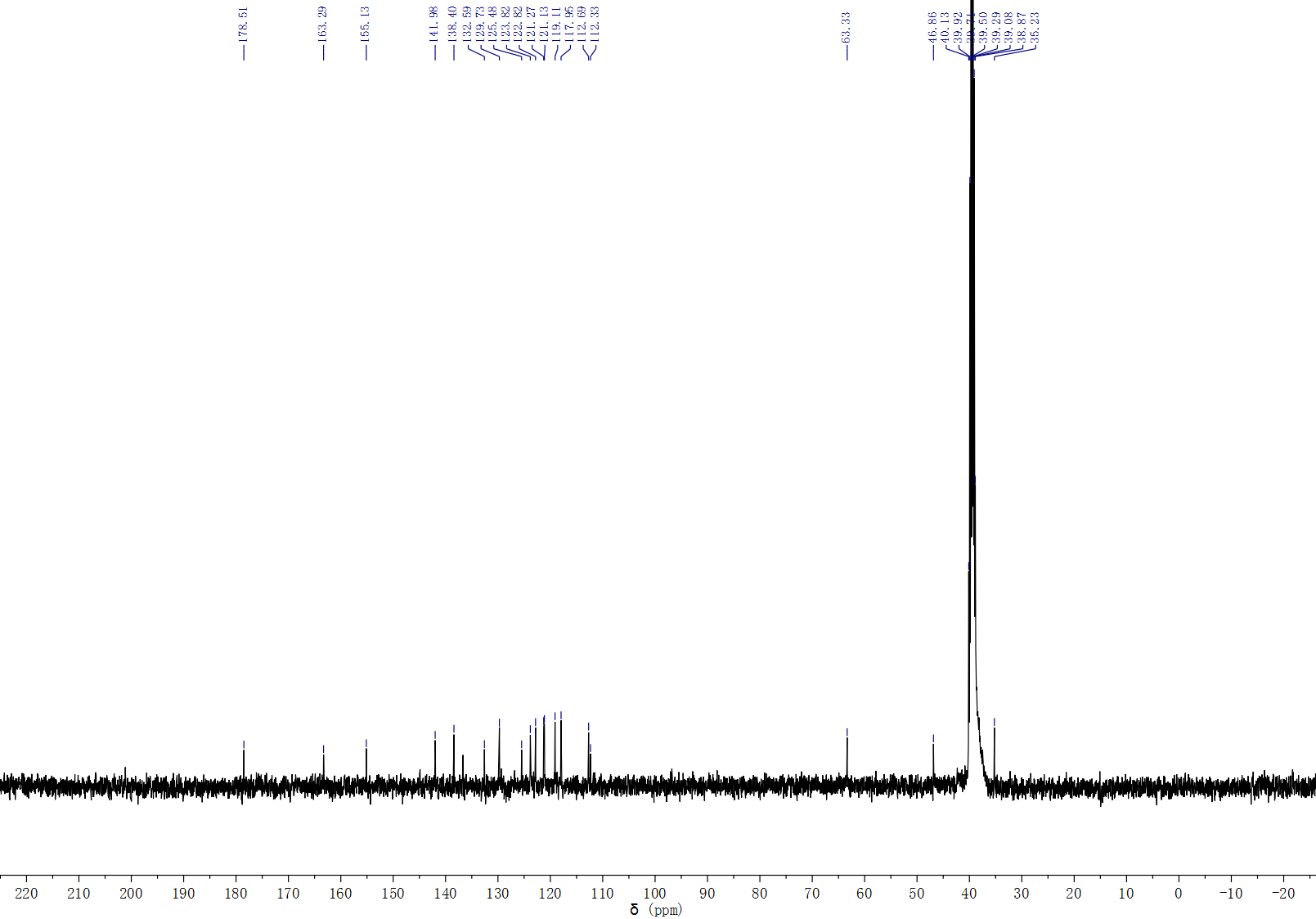


MS(ESI) spectrum of compound **17d**.

HPLC chromatogram of compound **17d**.


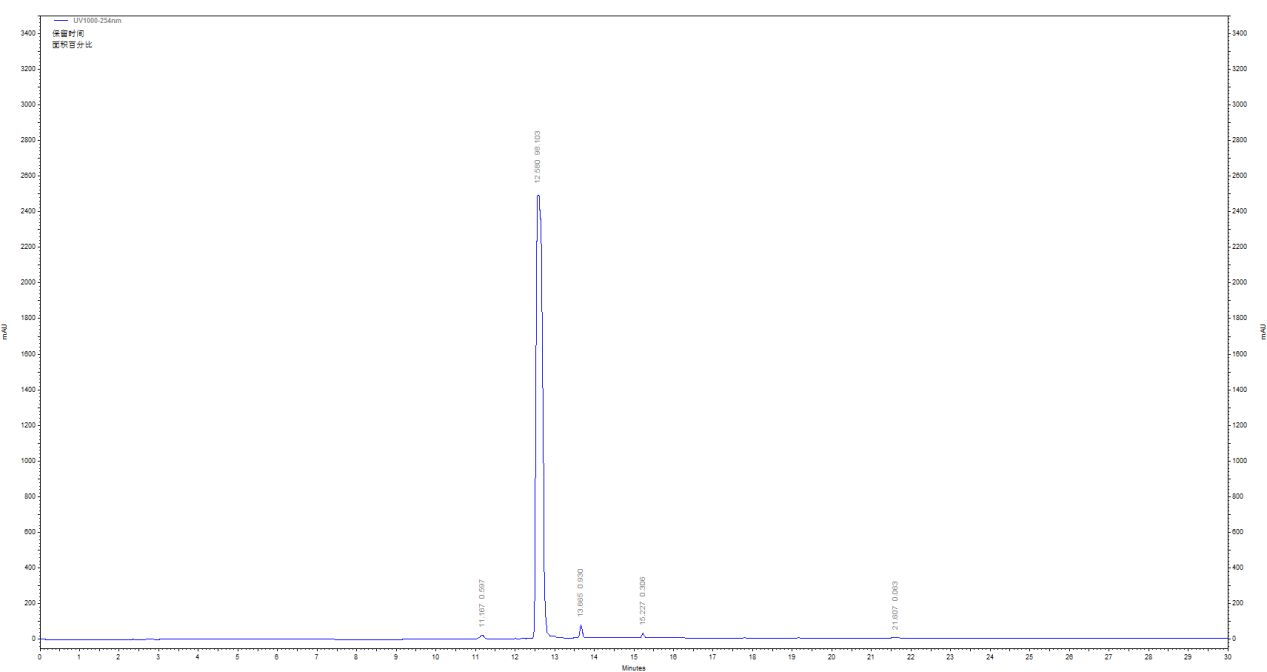

Supplement: Supplementary file 1 — Supplementary Information. [file 41598_2023_31849_MOESM1_ESM.docx]
